# Comparative Proteomics of Leaves from Phytase-Transgenic Maize and Its Non-transgenic Isogenic Variety

**DOI:** 10.3389/fpls.2016.01211

**Published:** 2016-08-17

**Authors:** Yanhua Tan, Xiaoping Yi, Limin Wang, Cunzhi Peng, Yong Sun, Dan Wang, Jiaming Zhang, Anping Guo, Xuchu Wang

**Affiliations:** ^1^College of Agriculture, Hainan UniversityHaikou, China; ^2^Key Laboratory of Biology and Genetic Resources for Tropical Crops, Institute of Tropical Biosciences and Biotechnology, Chinese Academy of Tropical Agricultural SciencesHaikou, China

**Keywords:** biosafety assessment, comparative proteomics, genetically modified crop, phytase-transgenic maize, unintended effect

## Abstract

To investigate unintended effects in genetically modified crops (GMCs), a comparative proteomic analysis between the leaves of the phytase-transgenic maize and the non-transgenic plants was performed using two-dimensional gel electrophoresis and mass spectrometry. A total of 57 differentially expressed proteins (DEPs) were successfully identified, which represents 44 unique proteins. Functional classification of the identified proteins showed that these DEPs were predominantly involved in carbohydrate transport and metabolism category, followed by post-translational modification. KEGG pathway analysis revealed that most of the DEPs participated in carbon fixation in photosynthesis. Among them, 15 proteins were found to show protein-protein interactions with each other, and these proteins were mainly participated in glycolysis and carbon fixation. Comparison of the changes in the protein and tanscript levels of the identified proteins showed that most proteins had a similar pattern of changes between proteins and transcripts. Our results suggested that although some significant differences were observed, the proteomic patterns were not substantially different between the leaves of the phytase-transgenic maize and the non-transgenic isogenic type. Moreover, none of the DEPs was identified as a new toxic protein or an allergenic protein. The differences between the leaf proteome might be attributed to both genetic modification and hybrid influence.

## Introduction

Genetically modified crops (GMCs) were first introduced to commercial agriculture in 1996, and approximately 181.5 million hectares of GMCs were grown worldwide in 2014. These GMCs have produced significant benefits over the past two decades (Clive, 2015). A recent meta-analysis by Klumper and Qaim concluded that the wide adoption of GM technology has reduced the usage of chemical pesticides, in addition to increasing crop yields to improve farmers' profits (Wilhelm and Matin, 2014). Despite the obvious positive effects of GMCs, public controversy over on the unintended, unexpected, and uncontrolled negative effects of GMCs are still ongoing. There is considerable concern that the introduction of exogenous DNA sequences and enzymes into the target plant genome in GMCs might result in unintended effects, and these negative effects may affect both human health and the environmental safety (Ioset et al., 2006). Therefore, determination of these potential unintended effects necessary and scientists should perform bio-assessment analyses to guarantee the safety of GMCs.

To detect such potential unintended effects, the recently developed global profiling technique may be a useful approach (Kuiper et al., 2001). Omics-based studies, including transcriptomics (mRNA profiling), proteomics (protein profiling) and metabolomics (metabolite profiling), have already been performed in several GMCs such as maize, barley and rice, and have been shown to be powerful techniques (Gong and Wang, 2013). Among these profiling techniques, proteomics approaches are direct methods for investigating unintended effects at the protein level. Thus, comparison of the entire proteomic profiles of GMCs and their corresponding wild-type lines can provide detailed information on DEPs (differentially expressed proteins) that are involved in metabolism and cellular development or those that play roles as toxins, antinutrients and allergens. Two-dimensional electrophoresis (2-DE) combined with mass spectrometry (MS) has been widely used in proteomics research, and this technology has been recently used to compare the protein profiles of various maize varieties, notably the MON810 maize varieties and their control lines, due to their potential commercial values (Gong and Wang, 2013). Studies have shown that there are some differences between GMCs and their control lines (Albo et al., 2007; Zolla et al., 2008; Balsamo et al., 2011; Coll et al., 2011; Vidal et al., 2015), but the observed differences are not substantial and may be caused by environmental factors (Albo et al., 2007). Many environmental factors play more important roles in shaping the proteomic profiles of transgenic crops than the transgene itself (Coll et al., 2011).

Maize is one of the most important feed crops in China, but phytase-overexpressing maize is the only GM maize that has been approved as a potential biosafety species to date in China and would be commercially planted in future. The transgenic maize line BVLA430101, which overexpresses an *Aspergillus niger* phytase (*phy*A2), was developed and recently licensed by the Chinese Academy of Agricultural Sciences (Chen et al., 2008), and is the only line that has been approved regarding crop biosafety by the Ministry of Agriculture of China since 2009. The phytase GM line carrying the *phy*A2 gene, together with a selectable *bar* marker gene at the same locus, specifically expresses the 60 kDa *phy*A2 protein of in its seeds, which exhibit higher phytase activity than non-transgenic maize seeds (Chen et al., 2008). Phytases (InsP_6_ phosphohydrolases) are a special class of phosphatase that catalyzes the sequential hydrolysis of phytic acid to produce less phosphorylated myo-inositol derivatives and inorganic phosphate (Pasamontes and Wyss, 1999). Phytase-transgenic maize can improve phosphorus availability and reduce the impact of animal production on the environment (Chen et al., 2008). Many studies had been performed to ensure the safety of phytase-transgenic maize, including evaluation of its nutritional value (Gao et al., 2012), of the effects related to its use as livestock feed (Li et al., 2013), and of the effects on arthropod communities in maize fields (Zhang Y. et al., 2010). However, most of these studies were target-oriented, and there have been no studies investigating untargeted effects through proteomics analysis.

The leaf is an important organ of green plants due to its roles in plant energy capture and carbon conversion (Baerenfaller et al., 2012), and it is the site of many important biological processes, such as photosynthesis, respiration, and transpiration (Guo et al., 2014). Since leaves are directly released into the surroundings, leaf is one of the important contents in GM plants' environmental safety assessment. Moreover, leaves are an edible part of the plant for cattle, sheep and other livestock. Thus, it would be implicated to animal health to be feed the leaves. Therefore, a leaf proteomics analysis will be useful for the assessment of health and environmental risks as well as the investigation of unintended physiological effects (Balsamo et al., 2011). In this study, we compared the protein profiles of the leaves of phytase transgenic maize and the corresponding non-transgenic isogenic type using a 2-DE and MS-based approach to investigate the unintended effects in GM maize. We found that the proteomic patterns were not substantially altered in the leaf proteome between the phytase-transgenic maize and its isogenic type.

## Materials and methods

### Plant materials and growth conditions

In this study, the transgenic maize variety 10TPY006 with overexpression of the phytase gene (hereafter referred to as PT maize), and the conventional hybrid LIYU16, as the non-genetically modified control (hereafter referred to as NT maize), were used. Seeds of PT maize and NT line were provided by Beijing Origin Seed Technology Inc. LIYU16 is a hybrid variety with high yield and strong adaptability and has been widely planted in China. This NT line was derived by crossing the LIYU91158 and LIYU953 inbred lines. Using the *phy*A2 transgenic maize line BVLA430101 as a non-recurrent parent (gene donor) that provided by the Ministry of Agriculture of China, LIYU91158 and LIYU953 as recurrent parents, the *phy*A2 insertion was introduced into the LIYU16 background through three major steps. Firstly, the *phy*A2 insertion was introduced into the LIYU91158 and LIYU953 backgrounds *via* genetic crossing with the *phy*A2 transgenic maize line BVLA430101. Then, the resulting LIYU91158 and LIYU953 transgenic lines were backcrossed with the recurrent parents six times to minimize the mixed genetic background, followed by two self-pollinations to obtain homozygous plants (OSL931 and OSL930, respectively) of each inbred lines. Finally, the PT line LIYU006 was further bred by crossing OSL931 and OSL930 as its DNA fingerprint was close to that of LIYU16.

PT maize and NT seeds were germinated on water-saturated filter paper at 25°C in the dark for 48 h. Germinated seedlings were selected and then planted in soil. A total of 100 seedlings from each variety were grown side-by-side in an environmentally controlled growth chamber for an additional 10 days (16 h light/8 h dark, 100 photon μmol m^−2^ s^−1^, 25°C). Maize seedlings were randomly divided into three groups to provide three biological replicates. The leaves were collected, frozen in liquid nitrogen, and stored at −80°C for further study.

### Determination of the event-specific sequence

Genomic DNA was isolated from the leaves *via* the CTAB method. Total RNA was isolated from the leaves using the Trizol method using TIRpure reagent (Bioteke, China). PCR was performed using specific primers as described previously (Yu et al., 2012) to confirm the presence of the exogenous phytase gene in the transgenic maize. The *phy*A2 gene fragment was amplified with the event-specific primers: P-F (5′-AATTGCGTTGC GCTCACT-3′) and P-R (5′- GCAACACATGGGC ACATACC -3′); *bar*-F (5′-GAAGGCACGCA ACGCCTACGA-3′) and *bar*-R (5′-CCAGAAAC CCACGTCATGCCA -3′) primers were used for the *bar* gene; and the *zSSIIb* gene was amplified with the primers of *zSSIIb*-F (5′-CGGTGGATGCTA AGGCTGATG-3′) and *zSSIIb*-R (5′-AAAGGGC CAGGTTC ATTATCCTC-3′) to act as an internal control. Reference materials were provided by Beijing Origin Seed Technology Inc. and were used as a control. Semi-quantitative RT-PCR was performed to examine the expression of exogenous genes in the transgenic maize. The *phy*A2 event-specific primers used for RT-PCR were P-F (5′-TCAAACCCTTCACG AAGCTATCCC-3′) and P-R (5′-TACTTTCCCGCTCAA CTCCACTCT-3′) (Zhang Q. et al., 2010).

### Protein extraction

Total leaf proteins of three groups were extracted by a modified Borax/PVPP/Phenol (BPP) protein extraction method described by Wang et al. (2007). The frozen maize leaves were ground in liquid nitrogen using a mortar and pestle. Approximately 3 g of the fine powders were resuspended in the 10 mL extraction buffer. The mixtures were then vortexed for 5 min at room temperature, and an equal volume of Tris-saturated phenol (pH 8.0) was added and vortexed further for 10 min. Then the mixtures were centrifuged (16,000 g, 15 min, 4°C), and the upper phase was transferred into a new centrifuge tube and clarified twice. After that, protein precipitates were obtained by adding five volumes of ammonium sulfate saturated-methanol and incubating at −22°C for at least 6 h. The precipitated proteins were centrifuged and air-dried, then recovered with the lysis buffer. Protein concentration was determined by the Bradford method using the UV-160 spectrophotometer (Shimadzu, Kyoto, Japan). Bovine serum albumin (BSA) was used as the protein standard (Bradford, 1976).

### 2D electrophoresis

IPG strips and IPG buffer were purchased from GE Healthcare. Chemicals for staining procedures, iodoacetamide and DTT were purchased from Sigma. 2-DE was performed according to the manufacturer's instruction (2-DE Manual, GE Healthcare) with some modifications. Protein samples about 1300 μg were diluted to 450 μl with lysis buffer (7 M urea, 2 M thiourea, 2% CHAPS, 13 mM DTT), and loaded onto a 24 cm IPG strip (immobilized pH gradient) with linear pH gradient 4–7 (GE Healthcare, Uppsala, Sweden). The strips were hydrated for 18 h at room temperature. IEF was performed at 20°C on an Ettan IPGphor isoelectric focusing system as follows: 3 h at 250 V, 2 h at 500 V, 1 h at 1000 V, a gradient to 8000 V for 3 h, and 8000 V up to 110,000 Vhr for strips. After IEF, the IPG strips were equilibrated for 15 min in equilibration solution (50 mM Tris-HCl pH 8.8, 6 M urea, 30% glycerol, 2% SDS and 0.002% bromophenol blue) containing 1% DTT for the first equilibration step and 15 min in equilibration solution with 4% iodoacetamide for the second. Then the strips were transferred to an Ettan Dalt system (GE Healthcare) to perform the SDS-PAGE. The second dimension was carried out at 5 W/gel for 1 h and then 8 W/gel for 5 h at 16°C, and was terminated when the bromophenol dye front had migrated to the lower end of the gels (Wang et al., 2007).

Gels were visualized by the GAP staining method as described (Wang et al., 2012). After staining, gels were scanned with ImageMaster Labscan V3.0 (GE Healthcare, Uppsala, Sweden) and image analysis was performed with the ImageMaster 2D Platinum software package (GE Healthcare, Uppsala, Sweden). To quantify the differential proteins in the leaves of PT maize and its NT, the Student's *t*-test was performed. In the statistical analysis only the spots presented in all three replicate gels that matched with its comparator were considered. Spots with Student's *t P* < 0.05 and at least 1.5-fold relative change in their quantities were further analyzed.

### Protein identification *via* MALDI TOF/TOF MS

The protein spots of interest were manually excised from 2-DE gels and digested in-gel with bovine trypsin (Trypsin, Roche, Cat. 11418025001) as described (Wang et al., 2009). Protein spots were first washed with MilliQ water three times for 30 min and destained three times with the destaining solution containing 50 mM NH_4_HCO_3_ and 50% ACN for 30 min each at 37°C, incubated in 100 μL of 100% ACN, and then air dried at room temperature for 1 h. After that, digestion was performed with 20 ng/μL trypsin solution, and incubated in trypsin buffer (25 mM NH4HCO3, 0.1 mM CaCl_2_, PH 8.0) for 16 h at 37°C.

The digested protein peptides were mixed with R-cyano-4-hydroxycinnamic acid (CHCA) matrix for peptide map fingerprinting (PMF), and analyzed using a AB SCIEX MALDI TOF-TOF 5800 system (AB SCIEX, Shanghai, China) equipped with a neodymium with laser wavelength 349 nm. Peptide mass fingerprints were obtained as described (Yi et al., 2014). The first full-scan mass spectrum was measured for range 800–4000 m/z and the second scan was done to measure the collision-induced MS/MS spectrum of the selected ions (range 1000–23,000 m/z).

The raw MS and MS/MS spectrum data were combined together and submitted to the database using the MASCOT software in-house for protein identification. *Zea may* (including 87,603 sequences) was chosen as the taxonomic category, and then the matched proteins specific for PT maize were searched against all entries using the MASCOT software. The search parameters were set as follows: enzyme-trypsin (cleavage at the C-term side of Lys and Arg unless the next residue was Pro); fixed modifications-carbamidomethyl (C); variable modifications-oxidation (M); no restrictions on protein mass; allow up to 1 missed cleavage. MS/MS ion tolerance was set as 0.1 Da and score was set as 62 (*p* < 0.05). If peptides matched to multiple members of a protein family, the one with the highest score was reported in this study for bioinformation analysis. Then, an in-house BLAST search using NCBI (http://www.ncbi.nlm) was performed for the unnamed proteins to find homologous proteins.

### Western blot analysis

About 20 μg of the isolated proteins were separated *via* SDS-PAGE and then transferred onto a polyvinylidene difluoride (PVDF) membrane (GE Healthcare) for Western blotting analysis. The blot was probed with a polyclonal antibody for phytase provided by the Biotechnology Institute, Chinese Academy of Agricultural Sciences (CAAS) (1:2000 dilution), and a goat anti-rabbit IgG-labeled with horseradish peroxidase (HRP) was used as the secondary antibody.

### Protein functional classification and pathway analysis

A local BLAST search against the UniProt database (http://www.uniprot.org/) was performed to map the identified proteins with functional annotations. The identified proteins were then categorized into the appropriate processes or functions by searching against the Gene Ontology database (http://www.geneontology.org) for the subsequent classification analysis using BLAST2GO software 3.0. Next, the identified proteins were clustered into different orthologous groups using UniProt and NCBI or based on the literature (Powell et al., 2014). Subcellular localization was predicted using CELLO V.2.5 (http://cello.life.nctu.edu.tw), which is based on a two-level support vector machine system (Yu et al., 2006). GO classification of the identified proteins was further performed using the WEGO software (http://wego.genomics.org.cn) by GO terms based on biological process, molecular functions, and cellular components (Ye et al., 2006). Finally, KEGG pathway analysis was conducted to determine the molecular interaction and reaction networks of the proteins using the BLAST2GO 3.0 software.

### Quantitative RT-PCR (qRT-PCR) analysis

Total RNA was isolated with the Trizol reagent, and 1 μg of RNA was used to generate cDNA with a reverse transcriptase kit (TaKaRa, Tokyo, Japan). The cDNA samples were diluted to 5–8 ng/μL. qRT-PCR reactions with a 20 μL volume were prepared in triplicate by adding 1 μL of each cDNA dilution to SYBR Green PCR Master Mix (TaKaRa) and run on an Mx3005P sequence detection system according to the manufacturer's instructions. The primer pairs used for qRT-PCR were provided in Table S2. Data were analyzed with MxPro software.

## Results

### Detection of exogenous genes and target protein in PT maize leaves

**Figure 1:**
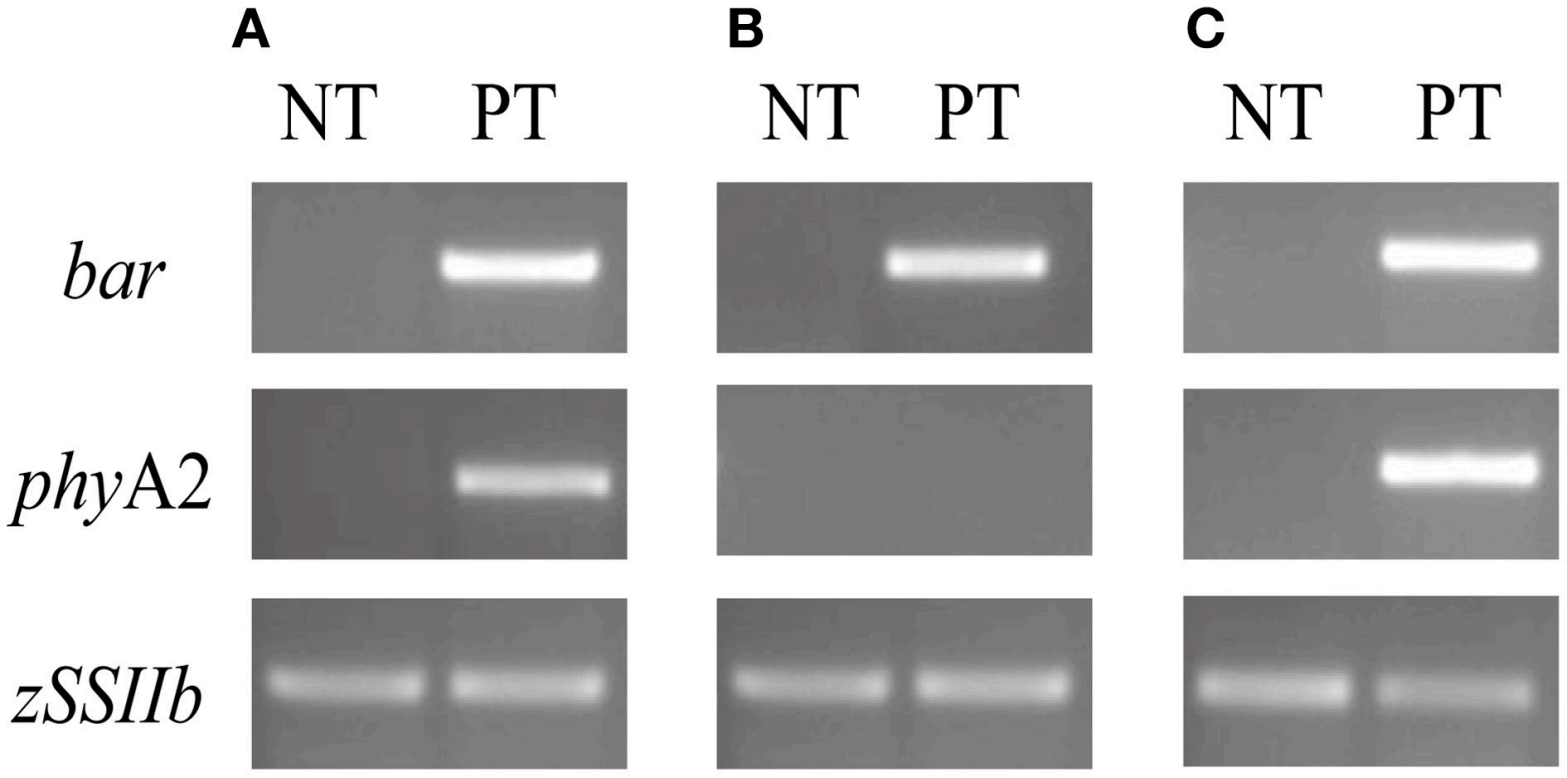
**PCR and RT-PCR analysis of the ***bar*** and ***phy***A2 transgenes. (A)** PCR results for exogenous genes in maize leaves; **(B)** RT-PCR results for exogenous genes in maize leaves; **(C)** RT-PCR results for exogenous genes in maize seeds. PT, phytase-transgenic maize 10TPY006; NT, the control variety LIYU16.

First, we analyzed the *phy*A2 and *bar* genes in the 10TPY006 transgenic maize line. As shown in Figure 1A and Figure S1, the target DNA fragments demonstrated a size of 152 bp for *phy*A2 gene and a size of 262 bp for *bar* gene, indicating that the exogenous event-specific gene sequence had been introduced into the genome of 10TPY006. However, no corresponding target genes were detected in the NT maize leaves. We further examined the expression of the exogenous genes *via* semi-quantitative RT-PCR. The results (Figure 1B, Figure S1) showed that only the *bar* gene could be detected, while the *phy*A2 transcript of was not detected in PT maize leaf tissues, which is consistent with the notion that the *phy*A2 gene under the control of the maize embryo-specific globulin-1 promoter is specifically expressed in maize seeds (Chen et al., 2008). A *phy*A2 transcript was detected in PT maize seed (Figure 1C, Figure S1). Neither *phy*A2 nor *bar*'s transcript was detected in the none-transgenic control variety LIYU16.

Western blot analysis revealed a band of approximately 60 kDa that was detected only in the PT maize seed samples (Figure S1). The band was not detected in PT maize leaf tissues and NT samples, indicating that phytase is mainly accumulated in the seeds.

### Comparison of protein profiles in leaves from PT and NT maize

Two-dimensional electrophoresis and image analysis were performed to compare protein profiles of the leaves of PT maize and the corresponding NT. The 2-DE maps of total proteins were obtained using IPG strips (pH 4–7) and 12% SDS-PAGE. For each group of protein extracts from PT maize and NT maize leaves, 2-DE gels were prepared in triplicate. More than 850 protein spots were detected in each 2-DE image after GAP staining with good reproducibility, and only the DEPs showing changes of >1. 5-fold were analyzed in detail. Analysis of the 2-DE images revealed 82 DEPs (33 with higher and 49 with lower abundance compared with NT) between the PT maize and NT maize leave samples.

### Protein identification *via* MALDI TOF/TOF MS

**Figure 2:**
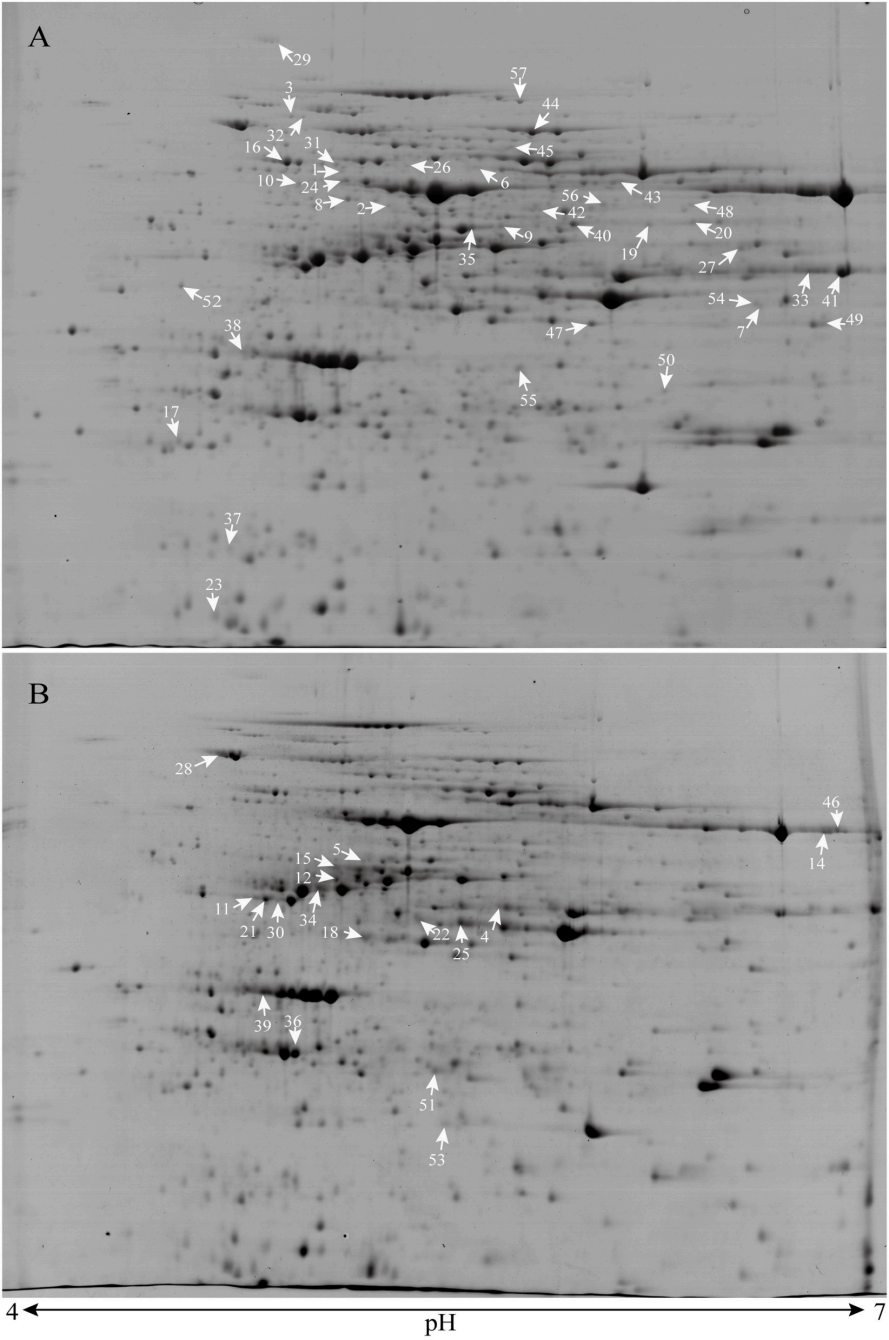
**Typical 2-DE gels of total leaf proteins from LIYU16 NT maize (A) and the 10TPY006 transgenic PT maize line (B)**. The 57 DEPs are indicated with arrows in the gel images. The numbers in the gels are proteins showing increased abundance in maize leaves.

A total of 82 DEPs were selected for MALDI TOF/TOF MS analysis after excision from the CCB-stained 2-DE gels and in-gel digestion with trypsin, and 57 protein spots were ultimately successfully identified through MS/MS analysis. Among these proteins, 20 were up-regulated, and 37 were down-regulated, as shown in Figure 2. Statistical information based on *t*-tests and the volume-averaged ratio of the identified protein spots was shown in Table 1, Figure S2. Protein identification was based on homology to *Zea mays* proteins. Several spots contained more than 1 protein identified *via* MS/MS (Table S3), and for peptides that matched several members of a protein family in maize, the one with the highest score was chosen. Among these spots, seven were termed as uncharacterized protein and were further chosen for BlastP (Protein-protein Blast) analysis in NCBI (http://blast.ncbi.nlm.nih.gov/Blast.cgi) to determine their protein identities (Table 1, Table S4).

**Table 1:** **Identification of the DEPs from maize leaves by MALDI TOF/TOF MS**.

**Spot No.^a^**	**Protein name**	**Protein Accession No.^b^**	**Theor. *p*I/M*r*^c^**	**Exper. *p*I/M*r*^d^**	**Matched peptide**	**Coverage (%)^e^**	**Mascot score**	***t*-test**	**Relative change^f^**
**CARBOHYDRATE TRANSPORT AND METABOLISM**									
1	Glucose-6-phosphate isomerase	A0A096R4M8_MAIZE	6.36/61.75	5.12/56.55	3	5	94	3.30e-05	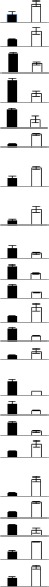
2	Phosphoglycerate kinase	K7V106_MAIZE	6.99/49.72	5.28/51.15	4	12	99	1.20e-05	
11	Sedoheptulose-1,7-bisphosphatase	B6T2L2_MAIZE	6.08/42.30	4.84/43.75	7	23	453	4.40e-41	
14	RuBisCO large chain	RBL_MAIZE	6.33/53.29	6.93/53.29	6	18	426	2.20e-38	
15	Phosphoglycerate kinase	C0PDB0_MAIZE	5.21/43.23	5.16/46.15	5	18	221	7.00e-018	
16	RuBisCO large subunit-binding protein subunit alpha	B6SXW8_MAIZE	5.20/61.42	4.95/61.42	5	12	301	7.00e-026	
19	3-phosphoadenosine 5-phosphosulfate synthetase	B6SRJ5_MAIZE	8.30/52.49	6.2/48.531	5	14	311	7.00e-27	
20	3-phosphoadenosine 5-phosphosulfate synthetase	B6SRJ5_MAIZE	8.30/52.49	6.33/48.66	6	17	234	3.50e-19	
21	Sedoheptulose-1,7-bisphosphatase	B6T2L2_MAIZE	6.08/42.30	4.90/42.67	8	26	425	2.80e-38	
22	Fructose-bisphosphate aldolase	C0PD30_MAIZE	6.37/38.41	5.48/41.48	7	34	770	8.80e-73	
25	Fructose-bisphosphate aldolase	C0PD30_MAIZE	6.37/38.41	5.62/41.32	7	34	883	4.40e-84	
27	Uncharacterized protein	B4FU39_MAIZE	7.19/43.95	6.52/44.25	6	24	688	1.40e-64	
30	Sedoheptulose-1,7-bisphosphatase	B6T2L2_MAIZE	6.08/42.30	4.95/42.56	8	26	571	7.00e-53	
33	Glyceraldehyde-3-phosphate dehydrogenase	Q6LBU9_MAIZE	7.21/41.27	6.75/41.54	4	14	408	1.40e-36	
34	Phosphoglycerate kinase	C0PDB0_MAIZE	5.21/43.23	5.08/43.24	6	29	596	2.20e-55	
36	Triosephosphate isomerase	B4FCE2_MAIZE	6.90/30.87	5.01/26.53	4	24	157	1.70e-11	
39	Oxygen-evolving enhancer protein 1	B6T3B2_MAIZE	5.59/34.78	4.88/31.24	3	16	133	1.10e-08	
41	Glyceraldehyde-3-phosphate dehydrogenase	Q6LBU9_MAIZE	7.21/41.27	6.88/41.26	5	18	481	7.00e-44	
43	RuBisCO large chain	P00874 _MAIZE	6.33/53.30	6.11/55.75	5	10	215	2.80e-17	
44	Transketolase isoform 1	K7V7B1_MAIZE	5.46/69.06	5.79/69.77	6	13	519	1.10e-47	
46	RuBisCO large chain	P00874 _MAIZE	6.33/53.30	6.95/50.13	9	18	701	7.00e-66	
52	Fructose-bisphosphate aldolase	C0PD30_MAIZE	6.37/38.41	4.53/39.92	6	32	322	5.50e-28	
56	Sucrose-phosphatase1	K7V496_MAIZE	7.04/30.03	6.05/51.45	2	7	64	3.90e-02	
**INORGANIC ION TRANSPORT AND METABOLISM**									
13	monodehydroascorbate reductase (NADH)	C4J4E4_MAIZE	5.45/46.82	5.59/46.64	3	7	113	4.40e-07	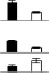
18	Ferredoxin–NADP reductase	B6TEW2_MAIZE	8.37/37.88	5.27/38.44	3	14	163	4.40e-12	
49	Ferredoxin–NADP reductase	B6TEW2_MAIZE	8.37/34.26	6.8/35.35	3	11	134	4.40e-09	
**AMINO ACID TRANSPORT AND METABOLISM**									
9	Acetylornithine deacetylase	B6TIJ2_MAIZE	5.45/49.49	5.64/48.52	5	15	198	1.40e-15	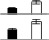
42	Histidinol dehydrogenase, chloroplastic	B8A2L1_MAIZE	5.41/47.19	5.82/50.44	2	7	102	5.50e-06	
**POSTTRANSLATIONAL MODIFICATION, PROTEIN TURNOVER, CHAPERONES**									
12	RuBisCO activase, chloroplastic	Q9ZT00_MAIZE	6.29/48.10	5.17/45.37	7	19	405	5.50e-36	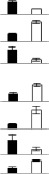
17	2-cys peroxiredoxin BAS1	C4J9M7_MAIZE	5.81/28.48	4.54/24.15	4	27	129	1.10e-08	
28	Filamentation temperature-sensitive H 2B	B1P2H4_MAIZE	5.69/72.61	4.73/75.68	4	9	203	4.40e-16	
31	Putative TCP-1/cpn60 chaperonin family protein	C0P530_MAIZE	5.42/61.99	5.1/60.76	4	8	157	1.70e-11	
38	14-3-3-like protein	B4FRG1_MAIZE	4.8/29.41	4.75/31.86	3	16	125	2.80e-08	
45	Uncharacterized protein OS	A0A096S2Q4_MAIZE	5.62/72.90	5.71/65.78	5	12	104	3.50e-06	
57	Cytokinin inducible protease1	C0PFV4_MAIZE	6.24/102.15	5.75/102.12	6	8	160	8.80e-12	
**TRANSLATION, RIBOSOMAL STRUCTURE, AND BIOGENESIS**									
29	Elongation factor Ts	C4J1J9_MAIZE	5.44/47.10	4.92/186.10	2	5	80	9.20e- 04	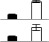
35	Elongation factor Tu	C0P7R5_MAIZE	4.91/41.47	6.00/47.79	8	28	637	1.70e-59	
**ENERGY PRODUCTION AND CONVERSION**									
6	ATP synthase subunit alpha	A0A059Q6M3_MAIZE	5.87/55.71	5.90/59.01	5	11	111	7.00e-07	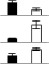
24	Putative ATPase, subunit B protein	B6UHI4_MAIZE	5.07/54.17	5.22/56.54	7	17	285	2.80e-24	
26	ATP synthase subunit alpha	A0A059Q6M3_MAIZE	5.87/55.71	5.35/59.23	4	13	127	1.70e-08	
**LIPID TRANSPORT AND METABOLISM**									
48	Uncharacterized	A0A0B4J3F5_MAIZE	6.29/56.53	6.33/51.86	7	18	192	5.50e-15	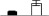
**CELL CYCLE CONTROL, CELL DIVISION, CHROMOSOME PARTITIONING**									
4	O-methyltransferase (Fragment)	Q6VWE9_MAIZE	5.48/39.17	5.79/41.77	2	7	79	1.10e-03	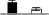
**SIGNAL TRANSDUCTION**									
37	Calmodulin	B6SLW1_MAIZE	4.44/18.88	4.68/18.88	3	23	62	5.30e-02	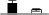
**CYTOSKELETON**									
8	Tubulin alpha-1 chain	TBA1_MAIZE	4.89/50.38	5.08/52.76	5	17	186	2.20e-14	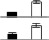
10	Tubulin beta-5 chain	TBB5_MAIZE	4.79/50.70	4.97/55.49	8	22	308	1.40e-26	
**COENZYME METABOLISM**									
5	Delta-aminolevulinic acid dehydratase	A0A096TKH4_MAIZE	5.97/46.43	5.30/47.21	4	7	123	4.40e-08	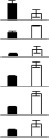
7	Pyridoxin biosynthesis protein ER1	B4FRZ2_MAIZE	6.12/33.83	6.60/37.12	4	14	296	2.20e-25	
23	oxygen evolving enhancer protein 3	B6SP64_MAIZE	7.66/25.91	4.63/14.46	1	7	95	7.30e-07	
40	S-adenosylmethionine synthase	B8A068_MAIZE	5.57/43.45	5.93/48.38	8	29	716	2.20e-67	
54	Pyridoxin biosynthesis protein ER1	B4FRZ2_MAIZE	6.12/33.83	6.59/37.52	3	11	110	8.80e-07	
55	Isomerase	B6SS56_MAIZE	7.67/33.78	5.73/29.96	2	8	62	5.70e-02	
**UNCLEAR CLASSIFICATION**									
3	Uncharacterized protein	A0A096SD19_MAIZE	4.90/67.86	4.95/85.76	7	13	349	1.10e-03	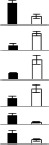
32	Uncharacterized protein	A0A096SD19_MAIZE	4.90/67.864	4.99/83.55	2	5	80	8.80e-04	
47	Uncharacterized	B4FLE1_MAIZE	5.64/33.05	5.94/34.56	3	12	131	7.00e-09	
50	Stem-specific protein TSJT1	B4FQW0_MAIZE	5.23/25.05	6.23/28.45	2	8	131	7.00e-09	
51	Uncharacterized protein	A0A096QKN7_MAIZE	9.50/32.07	5.55/25.34	3	12	309	1.10e-26	
53	Germin-like protein	Q6TM44_MAIZE	6.02/22.10	5.57/22.45	1	8	66	2.00e-02	

a *Assigned spot numbers as indicated in Figure 2*.

b *Database accession numbers according to UNIProt*.

c, d *The theoretical (c) and experimental (d) values of molecular weight (Mr, kDa) and pI for the identified proteins*.

e *Percent values of coverage (%) of the matched peptides in the whole protein sequence*.

f *Average abundance volume value of the target protein spots in the whole 2-DE gels*.

**Figure 3:**
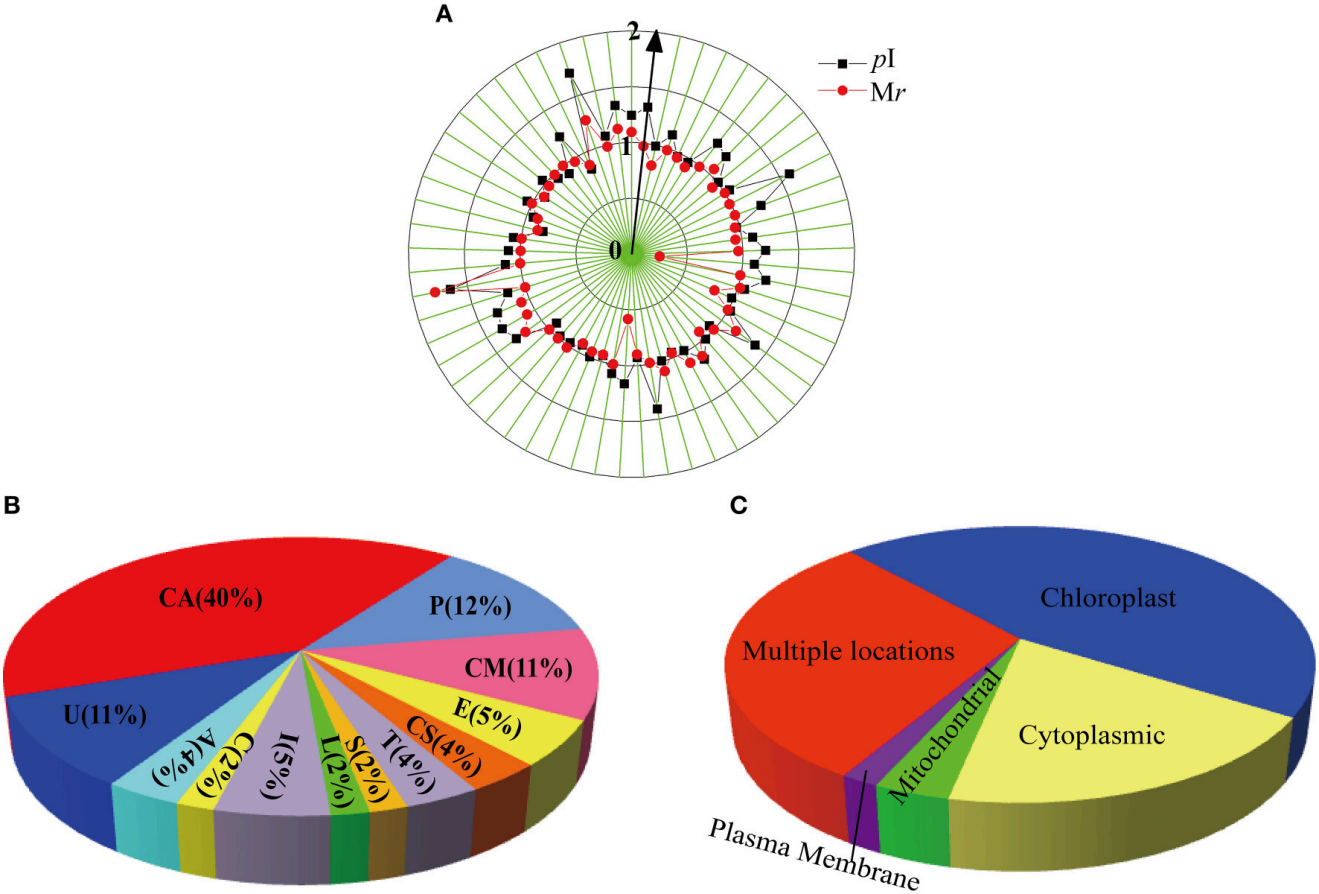
**Radial chart (A), functional classification (B) and subcellular location (C) of the identified 57 DEPs**. The theoretical and experimental ratios of the molecular mass (M*r*) and isoelectric points (*p*I) are presented in the radial chart. Functional catalogs were produced by COG, and the results are provided as the proportion of each functional category in all identities. The subcellular locations of the identified proteins are also presented. The abbreviations in the figures are as follows: CA, carbohydrate transport and metabolism; I, inorganic ion transport and metabolism; A, amino acid transport and metabolism; P, posttranslational modification, protein turnover, chaperones; T, translation, ribosomal structure and biogenesis; E, energy production and conversion; L, lipid transport and metabolism; S, signal transduction; C: cell cycle control, cell division, chromosome partitioning; CS, cytoskeleton; CM, coenzyme metabolism; U, unclear classification.

Among the 57 identified spots, 44 unique proteins were isolated (Table S1). To evaluate the quality of the identified proteins, the theoretical and experimental ratios of the molecular mass (M*r*) and isoelectric point (*p*I) were determined and presented in a radial chart as the radial and annular radar axis labels, respectively (Figure 3A). The results showed that approximately 95% of the identified proteins exhibited a relative M*r* ratio in the range of 1.0 ± 0.4 and 93% of the identified proteins exhibited a relative *p*I ratio in the range of 1.0 ± 0.4, suggesting that most of the identified proteins displayed experimental M*r* and *p*I values that were similar to their theoretical values.

### Protein function analysis

The identified proteins were classified into different categories according to their main biological activities as defined by the COG functional catalog. A total of 57 identified proteins were grouped into 11 major categories; 40% of the identified proteins were related to carbohydrate transport and metabolism, 12% (7 proteins) were related to post-translational modification and 11% were related to coenzyme metabolism. Several proteins were classified into other pathways, including energy production and conversion (3 proteins), inorganic ion transport and metabolism (3 proteins), translation, ribosomal structure and biogenesis (2 proteins), the cytoskeleton (2 proteins), amino acid transport and metabolism (2 proteins), cell cycle control, cell division, chromosome partitioning (1 protein), signal transduction (1 protein), and lipid transport and metabolism (1 protein). A large portion including 6 proteins could not be classified through COG classification (Figure 3B, Table 1, Table S4).

The subcellular locations of the 57 identified proteins were predicted, among which, the largest number of proteins (26 proteins) were located in the chloroplasts, followed by 11 proteins in the cytoplasm. There were 2 mitochondrial proteins and 1 plasma membrane protein among the identified proteins (Figure 3C; Table S4). The remaining proteins showed two or three locations or had no detailed location information. These results suggested that a large number of DEPs were located in the chloroplasts and cytoplasm.

### Pathway analysis of the identified proteins using GO and KEGG

**Figure 4:**
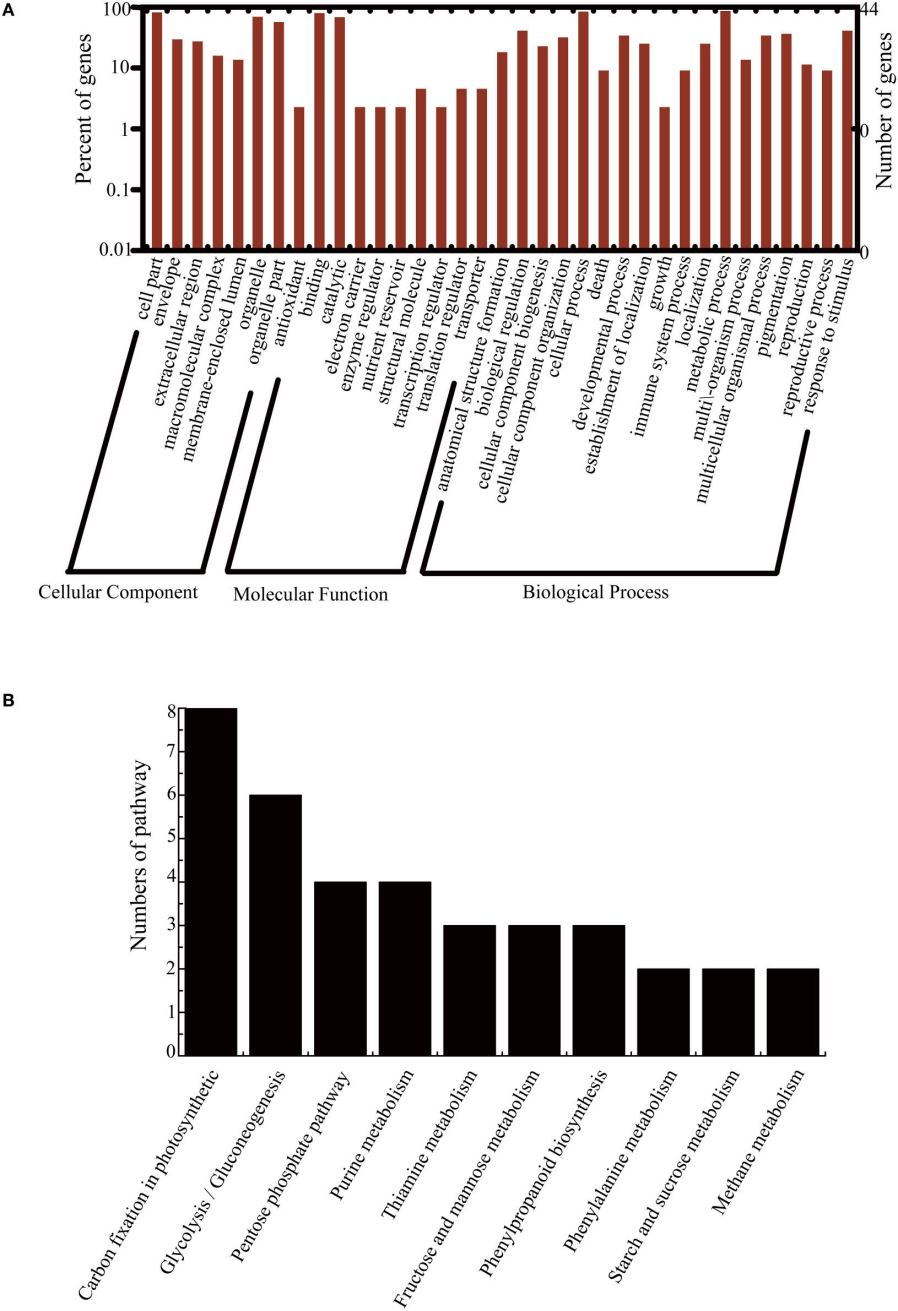
**WEGO output (A) and KEGG pathway (B) analysis of the identified 44 unique proteins**. To determine the functions of the identified differentially expressed proteins between NT and PT, GO analysis was performed using the WEGO software. A total of 41 identified proteins were available and classified into the 3 main categories of cellular components, biological processes, and molecular functions. They were then divided into 35 subgroups. To determine their molecular interaction and reaction networks, KEGG pathway analysis was also performed. The related pathways were classified into 10 main categories; one of the most important pathways was carbon fixation, which included 8 enzymes, followed by the glycolysis/gluconeogenesis pathway, with 5 enzymes.

To confirm the 44 unique DEPs between NT and PT maize in the cellular component, biological process, and molecular function categories, GO analysis was performed using the WEGO software (http://wego.genomics.org.cn/cgi-bin/wego/index.pl). GO information was obtained with BLAST2GO 3.0. Among the 44 DEPs, 41 were successfully mapped with GO annotations and classified into three ontologies that contained 35 GO terms, as shown in Figure 4A. At the cellular GO level, there were 8 total GO terms, corresponding to 36 proteins (about 81.8%) in the cells (GO: 0005623), 36 (81.8%) proteins in the cell part, and 31 proteins (70.5%) in the organelle (GO: 0043226); Regarding the molecular function ontology, 10 total GO terms were assigned, and the major functions were binding functions (GO: 0005488) with 35 proteins (79.5%), and catalytic activity (GO: 0003824) with 30 proteins (68.2%). In the biological process category, 18 GO terms were assigned; most of the DEPs were involved in metabolic processes (GO: 0008152) and cellular processes (GO: 0009987). The other important biological processes were the response to stimulus (GO: 0050896), biological regulation (GO: 0065007), pigmentation (GO: 0043473), developmental processes (GO: 0032502), multicellular organismal processes (GO: 0032501), and cellular component organization (Figure 4A, Table S5).

To determine the molecular interaction and reaction networks of the 44 DEPs, KEGG pathway analysis was performed using the BLAST2GO 3.0 program. There were 30 types of KEGG pathways in total; the most important pathway was carbon fixation in photosynthetic organisms, which contained 8 enzymes. These enzymes were SBPase (EC: 3.1.3.37, spot 21), isomerase (EC: 5.3.1.1, spot 36), carboxylase (EC: 4.1.1.39, spot 14), kinase (EC: 2.7.2.3, spots 2 and 34), hexose diphosphatase (EC: 3.1.3.11, spot 21), phosphorylating dehydrogenase (NADP^+^) (EC: 1.2.1.13, spot 41), glycolaldehydetransferase (EC: 2.2.1.1, spot 44), and aldolase (EC: 4.1.2.13, spot 22). The other major pathways were glycolysis/gluconeogenesis (5 enzymes with 6 sequences), the pentose phosphate pathway (4 enzymes), purine metabolism (2 enzymes with 4 sequences), fructose and mannose metabolism (3 enzymes), thiamine metabolism (1 enzyme), phenylpropanoid biosynthesis (3 enzymes), phenylalanine metabolism (2 enzymes), starch and sucrose metabolism (2 enzymes), and methane metabolism (2 enzymes). The remaining pathways contained 1 enzyme with 1 identified sequence (Figure 4B, Table S6).

### Comparison of protein and transcript expression patterns

**Figure 5:**
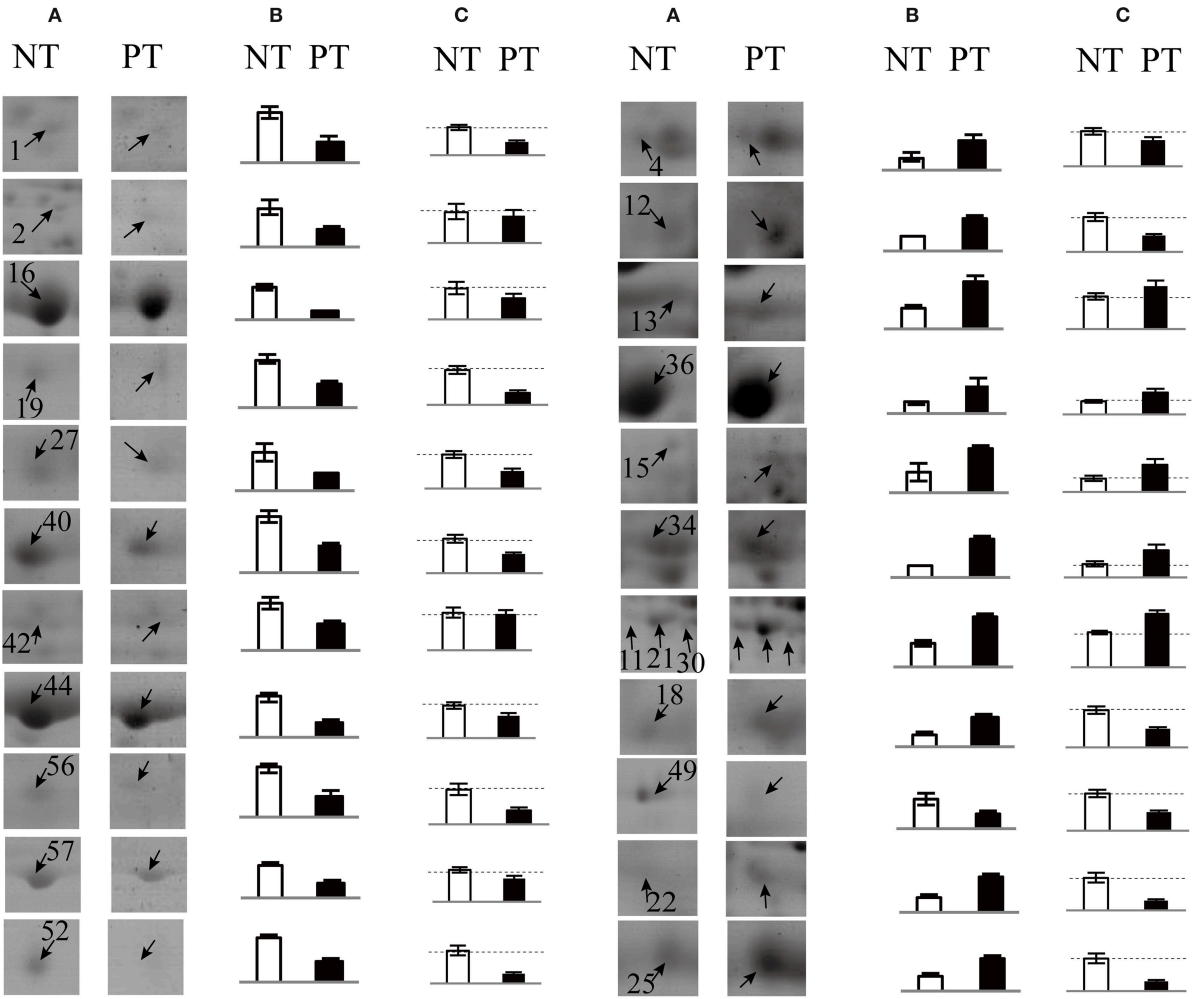
**Comparison of the expression patterns of 22 typical identified proteins at the protein and transcript levels**. The selected protein spots corresponding toDEPs in the 2-DE gels are shown **(A)**. Mean abundance values (Vol%) of the target protein spots in the 2-DE gels from PT and NT maize leaves **(B)**. qRT-PCR analysis of the gene expression patterns corresponding to the identified proteins in PT and NT maize leaves **(C)**. The gray dotted line in each qRT-PCR bar chart represents the 1.0 ratio value. Error bars represent the standard deviation (SD) of three replicates. Although several up-regulated proteins displayed a different pattern at the gene expression level, the comparisons showed that most genes and proteins exhibited a similar pattern in maize leaves.

To explore the changes in transcript levels, 22 identified proteins were chosen to conduct qRT-PCR analysis to validate the different gene expression patterns. The transcript level in the NT leaf template was set to 1.0 and the PT/NT fold-change ratios were obtained. Comparisons between the changes at the protein and mRNA expression levels of the identified proteins are shown in Figure 5. The results revealed that most proteins exhibited a similar pattern of changes at the translational and transcriptional levels, although for several up-regulated proteins decreases were observed at the transcriptional level. Several proteins were identified from 2 to 3 different protein spots at different points and their abundance was generally up-regulated; for example, sedoheptulose-1,7-bisphosphatase from spots 11, 21, and 30, and phosphoglycerate kinase from spots 15 and 34. However, several proteins identified from 2 to 3 protein spots showed different changes in protein abundance at different points, thus resulting in the inconsistency between the patterns of changes in protein and mRNA expression levels. For example, spots 22, 25, and 52 were identified as the same protein (fructose-bisphosphate aldolase), among which, spots 22 and 25 were up-regulated, but spot 52 was down-regulated in PT compared with NT. In general, the transcript level of this protein was down-regulated in NT (Figure 5).

## Discussion

### Comparative proteomics revealed many DEPs in the leaves of PT vs. NT maize

An approach for assessing the potential unintended effects of genetic modification has been proposed. The corner stone of safety assessment is the concept of *substantial equivalence* which is an internationally recognized standard (Konig et al., 2004). According to this concept, when comparing a new GM crop with a traditional counterpart that is generally accepted as safe based on the history of human food usage, the new GM crop is considered substantially equivalent to and as safe as its conventional counterpart if no sizeable differences are detected in the composition (OECD, 1993; FAO/WHO, 2000; EFSA, 2006).

In this study, 2-DE combined with MS was first employed to compare the proteomics of seedling leaves between phytase-transgenic maize and its non-transgenic isogenic counterpart, which had the closest genetic background. Our results suggested that there were detectable, but not substantial differences between PT and NT maize leaves. The 2-DE profiles revealed that approximately 82 DEPs could be detected but this number was less than 10% of the detectable protein spots in the 2-DE gels for the PT and NT maize leaf samples. These results were similar to previous studies, indicating that proteomic profiles are not dramatically altered after over-expression of target genes (Gong et al., 2012; Vidal et al., 2015). Moreover, the DEPs in the transgenic plants were not identified as new proteins, but rather as proteins showing changes in abundance, which is consistent with many other reported studies (Ruebelt et al., 2006; Ren et al., 2009). From a proteomics viewpoint, the expected difference between PT and NT lines in the ideal case is the presence of transgene-induced proteins. Random insertion of exogenous genes into the plant genome could lead to disruption of endogenous genes and rearrangement of the genome and unintended effects may occur (Gong and Wang, 2013), but a limited number of DEPs are expected to be affected by a single gene insertion (Arruda et al., 2013). Moreover, in previous studies, 11.69% of protein spots were found to show differences in accumulation in seedling leaves between a hybrid and its parental lines and a similar magnitude was observed at the transcriptional level (Swanson-Wagner et al., 2006; Guo et al., 2014). Hybridization can cause changes in the expression of a variety of proteins between hybrids and their corresponding inbred lines (Jin et al., 2013). In fact, such unintended effects are not unique to GM plants, they are also widely observed during conventional plant breeding (Ladics et al., 2015). GMCs do not show greatly altered proteomes compared with their natural genetic variats or with species obtained through conventional genetic breeding (Modirroosta et al., 2014; Vidal et al., 2015).

In this study, 57 protein spots were successfully identified representing 44 unique proteins. Several spots were identified as the same protein, these spots indicated the possible isoforms of each protein and may represent alternative splicing of transcripts or different post-translationally modified forms of the same protein (Guo et al., 2014). These spots also may be satellite spots due to artificial modifications as well as protein degradation. They may also result from experimental deviation. In plants, protein isoforms are commonly present due to post-translational modifications. These isoforms are also induced by transformation, conventional genetic breeding, and natural evolution and selection (Gong et al., 2012). Among the 44 identified unique proteins, most had a similar pattern of change at the protein and transcript levels, with several genes showed differential patterns. In the 2-DE gel patterns, most of the detected spots exhibiting a significant difference in relative abundance between the samples were low-intensity spots (Figure 2), which did not allow accurate measurement of relative protein abundance, especially for spot groups. If these low-intensity spots are not consistent with the main spots that remain unidentified, the pattern of changes at the protein and transcript levels will be different. Moreover, most of the DEPs corresponded to low-intensity spots, indicating that inserting genes would not alter high-abundance proteins but would instead only affect certain protein subunits and thus, that the transgene issue did not substantially alter the proteome.

Most of the identified proteins, even those that were unexpected, are naturally synthesized by the maize kernels. No changes in proteins known to be toxic or allergenic were detected in the present study, suggesting a lack of unintended effects under the applied testing conditions (Ren et al., 2009). According to the principle of *substantial equivalence*, the PT maize leaves of 10TPY006 can be judged to be substantially equivalent to the commercial maize variety LIYU16, and GM has not dramatically altered the proteome profiles of the maize leaves.

Phytase-transgenic maize overexpresses the *A. niger phy*A2 gene in its seeds, from a construct driven by the maize embryo-specific globulin-1 promoter. Based on the tissue specificity of *phy*A2 gene expression, we would not expect to identify the phytase protein as a DEP in this study. The selective marker protein phosphinothricin acetyl transferase (PAT), which is encoded by the *bar* gene, should theoretically be detected in the PT line but no in NT line due to the intended effects. However, in this study, we did not detect the intended protein, which was possibly due to both the low expression of the target gene and the limited accumulation of the target protein in PT leaves. In fact, in previous studies, the target protein was not detected at all (Zolla et al., 2008; Gong et al., 2012; Modirroosta et al., 2014). We had measured the Bt toxin protein content only 0.31 pg/g in cotton leaves by ELISA (Wang et al., 2015). In 2D electrophoresis experiment, gels were visualized by the GAP staining method, the detection limit of the Coomassie stain is approximately 100 ng/spot. The abundance of the target protein was below the detection limit of the Coomassie stain, which is consistent with the observations that made in other studies (Coll et al., 2011; Modirroosta et al., 2014).

### Many DEPs were involved in carbon fixation in PT maize

COG functional classification showed that approximately 40% of the DEPs were related to carbohydrate transport and metabolism, and KEGG analysis revealed that the DEPs between the NT and PT lines were predominantly involved in carbon fixation in photosynthetic organisms, glycolysis/gluconeogenesis, and pentose phosphate pathways. Our results showed that the largest group of metabolism-related DEPs, containing 8 enzymes, participated in the carbon fixation process in photosynthetic organisms. Among these enzymes, ribulose-1, 5-bisphosphate carboxylase (RuBisCO) (EC: 4.1.1.39) and sedoheptulose-1, 7-bisphosphatase (SBPase) (EC: 4.1.1.37) were two key enzymes. The photosynthetic carbon reduction (Calvin) cycle is the primary pathway for carbon fixation (Raines et al., 1999). CO_2_ fixation is performed through the Calvin cycle to drive sugar production, energy storage, and ultimately crop-yields (Wang et al., 2014). This cycle is considered to have three stages: carboxylation, reduction and regeneration. Rubisco catalyzes the first step in photosynthetic carbon fixation (Wachter and Henderson, 2015), using CO_2_ to carboxylate ribulose-1,5-bisphosphate (RuBP) to produce two molecules of 3-phosphoglycerate (3PGA) (Durall and Lindblad, 2015). This enzyme is one of the most important targets for improving the photosynthetic efficiency of vascular plants (Parry et al., 2013), and Rubisco with higher activity could increase photosynthesis in crops (Lin et al., 2014), which could ultimately enhance the crop yield (Mcgrath and Long, 2014). The enzyme SBPase participates in the final regenerative phase of the Calvin cycle by catalyzing the dephosphorylation of sedoheptulose 1,7-bisphosphate to produce the CO_2_ acceptor molecule RubP for the continued functions (Raines et al., 1999). This enzyme is unique to the Calvin cycle, and its activity affects photosynthesis, growth, and biomass allocation (Feng et al., 2009). It has been reported that decreased activity of SBPase can result in a significant reduction in the rate of light- and CO_2_-saturated photosynthesis (Harrison et al., 1998), and over-expression of SBPase can enhance carbon assimilation and crop yields (Rosenthal et al., 2011; Jessica et al., 2013). The 2-DE profiles obtained in the present study revealed that one ribulose-bisphosphate carboxylase (Rubisco) (spots 12), three ribulose bisphosphate carboxylase large chains (spots 14, 43, and 46), one Rubisco large subunit-binding protein (spots 16), and three isoforms of sedoheptulose-1, 7-bisphosphatase (SBPase) (spots 11, 21, and 30) identified as DEPs, with higher expression being observed in PT maize than in the NT line, except for spot 16 (Table 1, Figure S3, Table S4). For four Rubisco spots, they are low-intensity spots in 2D profiles (shown in Figure 2), they look like satellite spots of RuBisCO and the large spot of RuBisCO wasn't a DEP. Therefore, the quantifications of these satellite spots have been performed on a small fraction only and might not represent an accurate measurement of RuBisCO abundance. For SBPase, changes in protein and mRNA expression levels were all up-regulated. These up-regulated effects of Rubisco satellite spots and SBPase might be for generating extra energy in response to the insertion of exogenous genes (Gong et al., 2012). These findings are consistent with the results of another study on Bt-transgenic cotton leaves (Wang et al., 2015).

The main differences in the proteome profiles of PT and NT maize leaves were in functions and pathways such as carbohydrate transport and metabolism, glycolysis/gluconeogenesis, the pentose phosphate pathway, purine metabolism, and fructose and mannose metabolism. It is worth mentioning that the changes in the identified DEPs were not homogeneous. For example, although a total of 8 proteins were involved in the carbon fixation pathway, 5 of them were up-regulated, and 3 were down-regulated. Additionally, 6 proteins, including 4 up-regulated and 2 down-regulated proteins, were involved in glycolysis. These differences can be attributed to genetic modification and/or hybrid influences on the maize leaf proteome. For the carbon fixation and glycolytic pathways, the numbers of up-regulated proteins identified in PT leaves was twice that in the NT line. These variations may be due to positional effects of the gene insertion.

In conclusion, a proteomic comparison was performed for the first time to investigate DEPs to evaluate unintended effects in the leaves of phytase-transgenic maize. Proteomic analysis has provided much more information about such unintended effects than the data obtained *via* target-oriented analysis (Zolla et al., 2008), although the numbers of proteins that can be analyzed are still limited by 2-DE based proteomic analysis. It should be noted that unintended effects are not unique to GM plants, they are also widely observed during conventional plant breeding, and unintended effects do not necessarily indicate whether a plant is harmful (Ladics et al., 2015). Biosafety assessment of GM plants should be performed in a case-by-case manner. Our proteomics data for phytase-transgenic maize leaves may provide more information for the biosafety assessment of GM crops in the future.

## Author contributions

AG, XW conceived the project. YT, XY conducted research work and wrote manuscript. LW, CP, YS, and DW helped in statistical analysis. JZ reviewed the manuscript.

### Conflict of interest statement

The authors declare that the research was conducted in the absence of any commercial or financial relationships that could be construed as a potential conflict of interest.
