# Supplementary material for: Comparative Proteomics of Leaves from Phytase-Transgenic Maize and Its Non-transgenic Isogenic Variety
Source: Front Plant Sci. 2016 Aug 17;7:1211. doi: 10.3389/fpls.2016.01211 (PMC4987384; doi:10.3389/fpls.2016.01211)
Supplement: Supplementary file 1 [file DataSheet1.pdf]

Supplementary Material

Comparative Proteomics of the Leaves from Phytase-Transgenic Maize and its non-Transgenic Isogenic Variety

Yanhua Tan[1, 2 §], Xiaoping Yi[2 §], Limin Wang[2], Cunzhi Peng[2], Yong Sun[2], Dan Wang[2], Jiaming Zhang[1, 2], Anping Guo[1, 2]*, Xuchu Wang[1, 2]*

*** Correspondence:** Anping Guo, Xuchu Wang: xchwanghainan@163.com

1 Supplementary Data:

Supplementary Figure S1. Detection of the exogenous gene and target proteins in maize leaves and seeds.

Supplementary Figure S2. Identification of the differentially expressed proteins by MALDI TOF/TOF MS.

Supplementary Figure S3. Main pathways involved in transgenic maize proteins.

Supplementary Table S1. Classification of the 23 identified protein isoforms derived from 10 unique proteins.

Supplementary Table S2. Primers used in qRT-PCR.

Supplementary Table S3. MS/MS identified information.

Supplementary Table S4.MS identification and bioinformatics analysis of the DEPs.

Supplementary Table S5. GO numbers of the 44 unique proteins.

Supplementary Table S6. Blast2GO analysis of the 44 identified proteins.

2 Supplementary Figures and Tables

Supplementary Figure S1. Detection of the exogenous gene and target proteins in maize leaves and seeds.

Supplementary Figure S2. Identification of the differentially expressed proteins by MALDI TOF/TOF MS.

Supplementary Figure S3. Main pathways involved in transgenic maize proteins.

Supplementary Table S1. Classification of the 23 identified protein isoforms derived from 10 unique proteins.

Supplementary Table S2. Primers used in qRT-PCR.

Supplementary Figure S1. Detection of the exogenous gene and target proteins in maize leaves and seeds. The products of PCR (A,B,C) and RT-PCR (D) of exogenous genes expression of maize leaves were presented in agarose gel(1.5%), (E) was the RT-PCR results of maize seeds. PT: Phytase transgenes maize of 10TPY006; NT: control variety of LIYU16; K+: positive control. K-: negative control; K1: DNA isolated blank control; K2: PCR blank control; M: DNA marker. (F) was western blot analysis. Lane1, NT leaf control; lane 2, PT leaf; Lane3, NT seed control; lane 4, PT seed. A band of approximately 60 kD was detected only in lane 4.

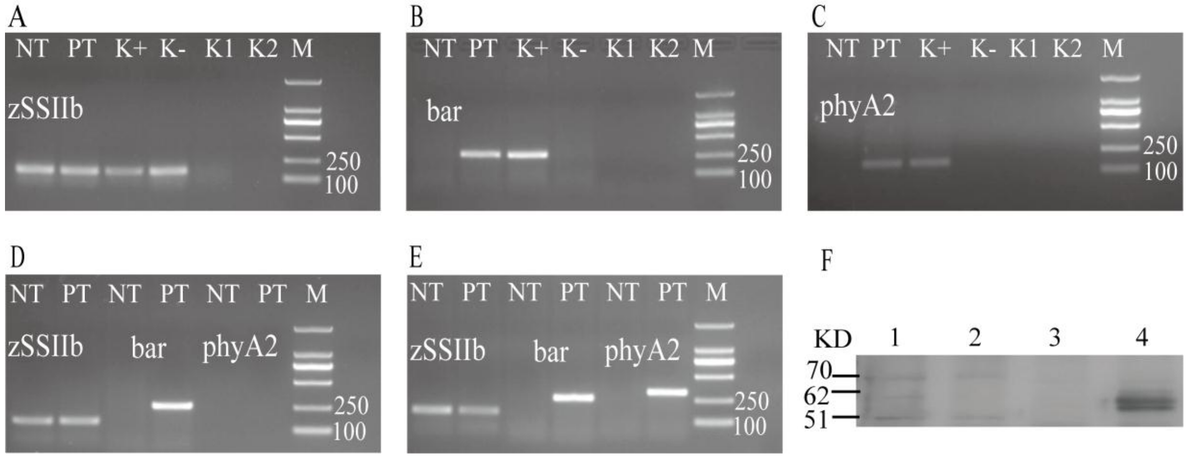

Supplementary Figure S2. Supplemental spectra and MALDI TOF/TOF MS/MS identification of the differentially expressed proteins.

Annotated spectra for Table 1: 57 proteins identified by PFF.

Spot numbers of the 57 proteins correspond to the proteins that listed in Table 1.

CID: collision induced dissociation

MALDI TOF:

matrix assisted laser desorption/ionization time of flight

MS: mass spectrometry

PFF: peptide fragment fingerprinting

Spot No.: **1**

Uniprot Protein Accession: **A0A096R4M8|A0A096R4M8_MAIZE**

Plant species: **Zea mays**

Protein name: **Glucose-6-phosphate isomerase**

Peptide sequences: **R.IEGWLAR.F; K.GSTDQHAYIQQLR.E ; R.AVGALIALYER.A**

PFF Mascot score: **[94]** Sequence coverage %: **[5]**

Matched peptides No.: **[3]** p value: **3.3e-005**

Calculated Mr: **61755** Calculated *p*I: **6.36**

PFF Searched Score:

Protein score is -10*Log(P), where P is the probability that the observed match is a random event.
Protein scores greater than 62 are significant (p<0.05).
Protein scores are derived from ions scores as a non-probabilistic basis for ranking protein hits.

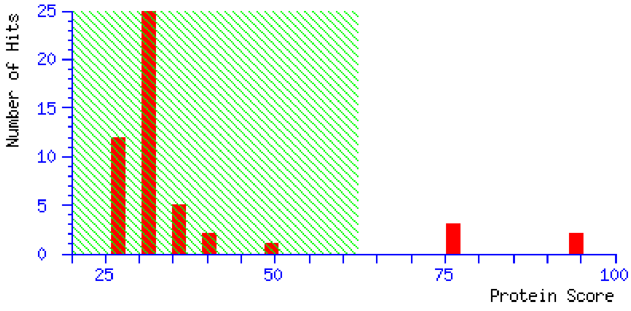

Matched peptide sequences shown in Bold Red

```
  1 MASISGAAAP PSSAVCRLRL RRHLLLRPSH LRLRAPHSIA DLSRSSNSAP
 51 SPARPALGSG PENGSGGVRA VEKDPIKLWE RYVEWLYQHK ELGIFVDVSR
101 MGFTEEFLRQ MEPRMQQAFV DMRELEKGAI ANPDEGRMVG HYWLRDPALA
151 PNSFLRNKIE TALDRILAFS QDVVSGKILS PSGRFTSILS IGIGGSALGP
201 QFVAEALAPD NPPLKIRFID NTDPAGIDHQ IAQLGPELAT TLVIVISKSG
251 GTPETRNGLL EVQKAFRDAG LQFSKQGVAI TQENSLLDNT ARIEGWLARF
301 PMFDWVGGRT SEMSAVGLLP AALQGIDIKE MLVGAALMDE ETRNTVVKAN
351 PAALLALCWY WASEGIGKKD MVVLPYKDSL LLLSRYLQQL VMESLGKEFD
401 LDGNRVNQGL TVYGNKGSTD QHAYIQQLRE GVQNFFVTFI EVLRDRPAGH
451 DWELEPGVTC GDYLFGMLQG TRSALYANDR ESISVTVQEV TPRAVGALIA
501 LYERAVGIYA SLVNINAYHQ PGVEAGKKAA GEVLALQKRV LTVLKEAMYG
551 TSDLCSFCFS VK
```

Spot No.: 2

Uniprot Protein Accession: **K7V106|K7V106_MAIZE**

Plant species: **Zea mays**

Protein name: **Phosphoglycerate kinase**

Peptide sequences: **R.ADLNVPLDENQNITDDTR.I; K.FSLAPLVAR.L ; K.LVAALPNGGVLLLENVR.F; K.LASVADLYVNDAFGTAHR.A.**

PFF Mascot score: **[99]** Sequence coverage %: **[12]**

Matched peptides No.: **[4]** p value: **1.2e-005**

Calculated Mr: **49720** Calculated *p*I: **6.99**

PFF Searched Score:

Protein score is -10*Log(P), where P is the probability that the observed match is a random event.
Protein scores greater than 62 are significant (p<0.05).
Protein scores are derived from ions scores as a non-probabilistic basis for ranking protein hits.

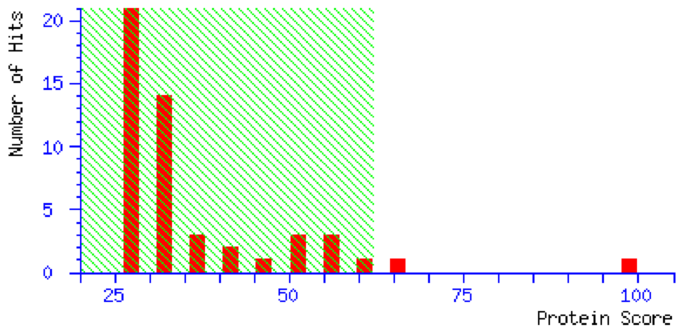

Matched peptide sequences shown in Bold Red

1 MASAAAPPTS LSLAARAASR AAAAPLRRGG LAAARQPARS LAFAAGDARL

51 AVHVASRCRQ ASSARGTRAM ATMAKKSVGD LTEADLKGKR VFVR**ADLNVP**

101 **LDENQNITDD** **TR**IRAAIPTI QYLLGKGAKV ILSSHLGRPK GFTPK**FSLAP**

151 **LVAR**LSELLG IQVQKADDVI GPEVEK**LVAA** **LPNGGVLLLE** **NVR**FYKEEEK

201 NDPEFAQK**LA** **SVADLYVNDA** **FGTAHR**AHAS TEGVTKFLKP SVAGFLLQKE

251 LDYLVGAVSS PTRPFAAIVG GSKVSSKIGV IESLLEKCDI LLLGGGMIFT

301 FYKAQGLSVG ASLVEEDKLD LATSLLAKAK EKGVSLMLPT DVVIADKFAP

351 DANSQIVPAS AIPDGWMGLD IGPDSIAAFN AALETTQTVI WNGPMGVFEY

401 DKFAIGTEAV AKKLAELSRK GVTTIIGGGD SVAAVEKVGV ADVMSHISTG

451 GGASLELLEG KELPGVTALD EAATVTV

Spot No.: **3**

Uniprot Protein Accession: **A0A096SD19|A0A096SD19_MAIZE**

Plant species: **Zea mays**

Protein name: **Uncharacterized protein**

Peptide sequences: **K.LAENEAALSLMR.D; K.TLTSLSEEFR.K ; K.GQEELEATSIELASIAEAR.D ; K.NLESTTHELVEER.K ; K.QLQVDSEAR.K ;K.ELESTHSR.S ; K.EQPVNDYNQK.T**

PFF Mascot score: **[349]** Sequence coverage %: **[13]**

Matched peptides No.: **[7]** p value: **1.1e-030**

Calculated Mr: **67864** Calculated *p*I: **4.90**

PFF Searched Score:

Protein score is -10*Log(P), where P is the probability that the observed match is a random event.
Protein scores greater than 62 are significant (p<0.05).
Protein scores are derived from ions scores as a non-probabilistic basis for ranking protein hits.

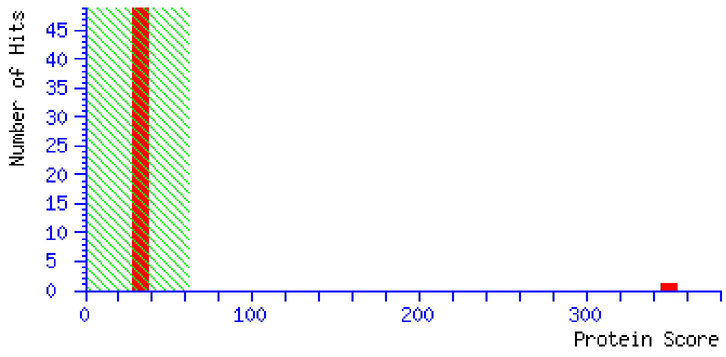

Matched peptide sequences shown in Bold Red

```
  1 MEIGDLIYSM FYLQMESKLA ENEAALSLMR DNYEKRLLEQ QAAQKKQSMK
 51 FQEQEVSLSG QLASATKTLT SLSEEFRKEK KLAEELRDEI QRLESSITQA
101 GIDNDVLETK LEEKLGEINF LQEKVSLLNQ EIDDKEKHIR ELSASLSSKE
151 VDYQKLTAFT NQTKKSLELA NSRVQQLEEE LSTTKNALVS KISSIDSLNA
201 KLETLNSEKE EADKKINELI QEYTDLKVAS ETRASHDSKL LSERDDLIKQ
251 LEEKLSVALT DSSKDQETIV ELNKELDATK MMLKNELKSM EALKDSIRSS
301 EEALKTSRSE VSKLSKELEE ANELNEDLVS QISKLREESN EMQVDLTNKL
351 GEAESLSKAL SEDLASVNEM VQKGQEELEA TSIELASIAE ARDNLKKELL
401 DAYKNLESTT HELVEERKIV TALNKELEAL AKQLQVDSEA RKSLESDLEE
451 ATKSLDEMNN SALLLSKELE STHSRSATLE SEKEMLRKAL AEQTKITTEA
501 KENTEDAQNL ITRLETEKES FELRCRHLEE ELALAKGEIL RLRRQISTNS
551 SQKPRARGPP EASETLKEQP VNDYNQKTSG VVAGTPQPVK RTVRRRKGGA
601
```

Spot No.: **4**

Uniprot Protein Accession:**Q6VWE9|Q6VWE9_MAIZE**

Plant species: **Zea mays**
Protein name: **O-methyltransferase (Fragment)**

Peptide sequences: **K.AALAPEEVVAR.M; K.AYGMTAFEYHGTDSR.F;**

PFF Mascot score: **[79]** Sequence coverage %: **[7]**

Matched peptides No.: **[2]** p value: **0.0011**
Calculated Mr:**39168** Calculated *p*I: **5.48**

PFF Searched Score:

Protein score is -10*Log(P), where P is the probability that the observed match is a random event.
Protein scores greater than 62 are significant (p<0.05).
Protein scores are derived from ions scores as a non-probabilistic basis for ranking protein hits.

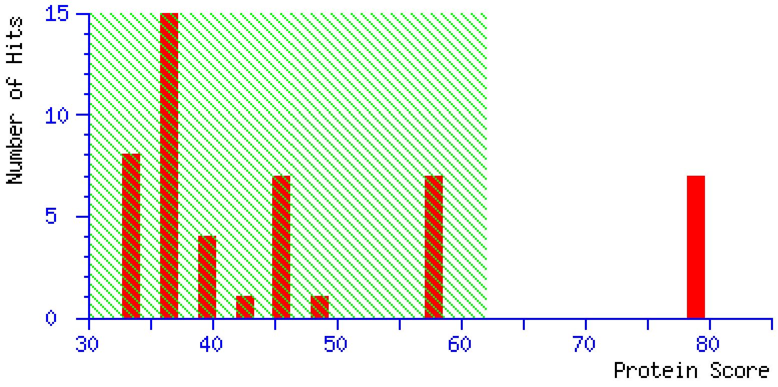

Matched peptide sequences shown in Bold Red

```
  1 MGSTAGDVAA VVDEEACMYA MQLASSSILP MTLKNAIELG LLEVLQKEAG
 51 GGKAALAPEE VVARMPAAPG DPAAAAAMVD RMLRLLASYD VVRCQMEDRD
101 GRYERRYSAA PVCKWLTPNE DGVSMAALAL MNQDKVLMES WYYLKDAVLD
151 GGIPFNKAYG MTAFEYHGTD SRFNRVFNEG MKNHSVIITK KLLDFYTGFE
201 GVSTLVDVGG GVGATLHAIT SRHPHISGVN FDLPHVISEA PPFPGVRHVG
251 GDMFASVPAG DAILMKWILH DWSDAHCATL LKNCYDALPE NGKVIVVECV
301 LPVNTEATPK AQGVFHVDMI MLAHNPGGKE RYEREFRELA KGAGFSGFKA
351 TYIYANAW
```

Spot No.: 5

Uniprot Protein Accession: **A0A096TKH4|A0A096TKH4_MAIZE**

Plant species: **Zea mays**

Protein name: **Delta-aminolevulinic acid dehydratase**

Peptide sequences: **K.SPTGDEAYNDNGLVPR.T ; K.YASSFYGPFR.E ; R.EALDSNPR.F ; R.EALDSNPR.F**

PFF Mascot score: **[123]** Sequence coverage %: **[7]**

Matched peptides No.: **[4]** p value: **4.4e-008**

Calculated Mr: **46434** Calculated *p*I: **5.97**

PFF Searched Score:

Protein score is -10*Log(P), where P is the probability that the observed match is a random event.
Protein scores greater than 62 are significant (p<0.05).
Protein scores are derived from ions scores as a non-probabilistic basis for ranking protein hits.

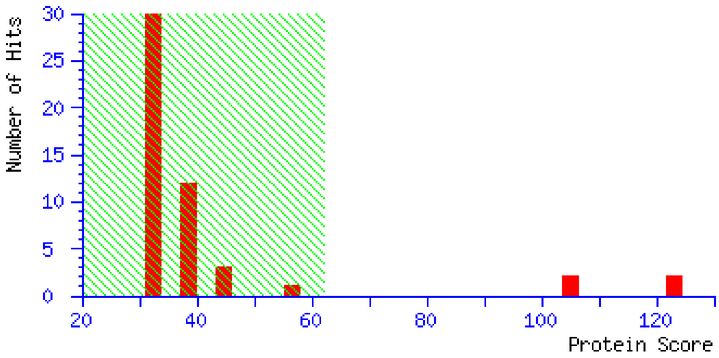

Matched peptide sequences shown in Bold Red

```
  1 MAFTVSFSPA NVQMLQARSG HGHATFGSCS AVPRAGPRLR STAVRVSSEQ
 51 EAAAAVRAPS GRTIEECEAD AVAGKFPAPP PLVRPKAPEG TPEIRPLDMA
101 KRPRRNRKSP ALRAAFQETS ISPANFVLPL FIHEGEEDAP IGAMPGCYRL
151 GWRHGLLDEV YKARDVGVNS FVLFPKVPDA LKSPTGDEAY NDNGLVPRTI
201 RLLKDKFPDI VIYTDVALDP YSSDGHDGIV REDGVIMNDE TVYQLCKQAV
251 SQARAGADVV SPSDMMDGRI GALRSALDAE GFHDVSIMSY TAKYASSFYG
301 PFREALDSNP RFGDKKTYQM NPANYREALI ETAADEAEGA DILLVKPGLP
351 YLDIIRLLRD HSALPIAAYQ VSGEYSMIKA GGALGMVDEQ KVMMESLMCL
401 RRAGADVILT YFARHAAAVL CGMGPK
```

Spot No.: **6**

Uniprot Protein Accession:**A0A059Q6M3|A0A059Q6M3_MAIZE**

Plant species: **Zea mays**
Protein name: **O-methyltransferase (Fragment)**

Peptide sequences: **R.VVQVGDGIAR.I;K.EAIQEQLER.F; R.LIESPAPGIISR.R; R.IAQIPVSEAYLGR.V;R.EAYPGDVFYLHSR.L**

PFF Mascot score: **[111]** Sequence coverage %: **[11]**

Matched peptides No.: **[2]** p value: **7e-007**
Calculated Mr:**55713** Calculated *p*I: **5.87**

PFF Searched Score:

Protein score is -10*Log(P), where P is the probability that the observed match is a random event.
Protein scores greater than 62 are significant (p<0.05).
Protein scores are derived from ions scores as a non-probabilistic basis for ranking protein hits.

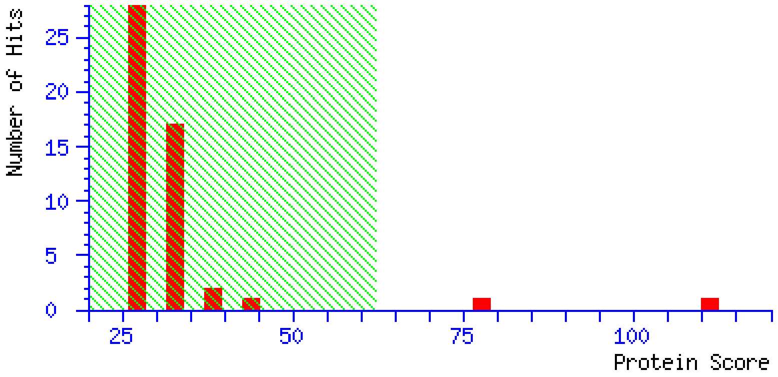

Matched peptide sequences shown in Bold Red

```
  1 MATLRVDEIN KILRERIEQY NRKVGIENIG RVVQVGDGIA RIIGLGEIMS
 51 GELVEFAEGT RGIALNLESK NVGIVLMGDG LMIQEGSFVK ATGRIAQIPV
101 SEAYLGRVIN ALAKPIDGRG EIVASESRLI ESPAPGIISR RSVYEPLQTG
151 LIAIDSMIPI GRGQRELIIG DRQTGKTAVA TDTILNQKGQ DVICVYVAIG
201 QRASSVAQVV TTFHEEGAME YTIVVAEMAD SPATLQYLAP YTGAALAEYF
251 MYRERHTLII YDDLSKQAQA YRQMSLLLRR PPGREAYPGD VFYLHSRLLE
301 RAAKLNSLLG EGSMTALPIV ETQSGDVSAY IPTNVISITD GQIFLSADLF
351 NAGIRPAINV GISVSRVGSA AQIKAMKQVA GKSKLELAQF AELQAFAQFA
401 SALDKTSQNQ LARGRRLREL LKQSQSNPLP VEEQVATIYT GTRGYLDSLE
451 IEQVKKFLDE LRKHLKDTKP QFQEIISSSK TFTEQAETLL KEAIQEQLER
501 FSLQEQT
```

Spot No.: **7**

Uniprot Protein Accession:**B4FRZ2|B4FRZ2_MAIZE**

Plant species: **Zea mays**

Protein name: **Pyridoxin biosynthesis protein ER1**

Peptide sequences: **K.VGLAQMLR.G; R.GGVIMDVVTPEQAR.L; R.LAEEAGACAVMALER.V; R.VPFVCGCR.D**

PFF Mascot score: **[296]** Sequence coverage %: **[14]**

Matched peptides No.: **[4]** p value: **2.2e-025**

Calculated Mr:**33832** Calculated pI: **6.12**

PFF Searched Score:

Protein score is -10*Log(P), where P is the probability that the observed match is a random event.
Protein scores greater than 62 are significant (p<0.05).
Protein scores are derived from ions scores as a non-probabilistic basis for ranking protein hits.

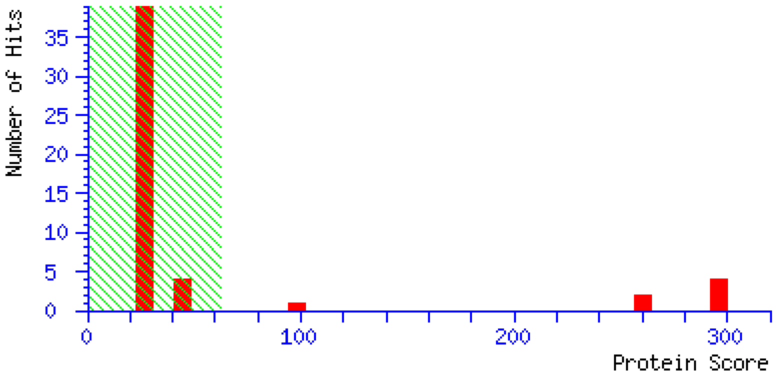

Matched peptide sequences shown in Bold Red

```
  1 MASDGSGVVT VYGSNGAELL EPSKQPKSAT FSVKVGLAQM LRGGVIMDVV
 51 TPEQARLAEE AGACAVMALE RVPADIRAQG GVARMSDPGL IRDIKRAVTI
101 PVMAKARIGH FVEAQILEAV GVDYVDESEV LTPADDAHHI NKHNFRVPFV
151 CGCRDLGEAL RRVREGAAMI RTKGEAGTGN VVEAVRHVRS VMGDVRALRS
201 MDDDEVFAYA KRIAAPYDLV MQTKQLGRLP VVQFAAGGVA TPADAALMMQ
251 LGCDGVFVGS GIFKSGDPAR RARAIVQAVT HYSDPTILAD VSTGLGEAMV
301 GINLNDPKVE RYAARSE
```

Spot No.: **8**

Uniprot Protein Accession: **P14640|TBA1_MAIZE**

Plant species: **Zea mays**

Protein name: **Tubulin alpha-1 chain**

Peptide sequences: **R.AVFVDLEPTVIDEVR.T; R.QLFHPEQLISGK.E; K.EDAANNFAR.G ; R.FDGALNVDVNEFQTNLVPYPR.I; R.AFVHWYVGEGMEEGEFSEAR.E**

PFF Mascot score: **[186]** Sequence coverage %: **[17]**

Matched peptides No.: **[5]** p value: **2.2e-014**

Calculated Mr: **50384** Calculated *p*I: **4.89**

PFF Searched Score:

Protein score is -10*Log(P), where P is the probability that the observed match is a random event.
Protein scores greater than 62 are significant (p<0.05).
Protein scores are derived from ions scores as a non-probabilistic basis for ranking protein hits.

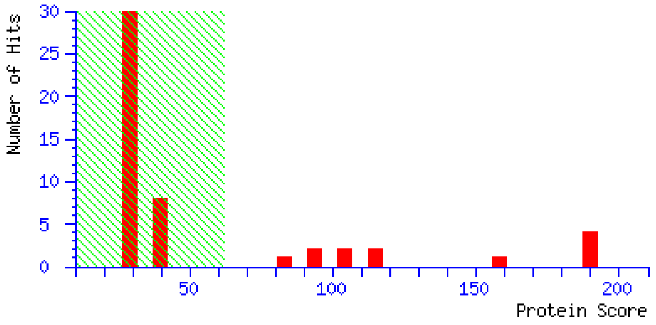

Matched peptide sequences shown in Bold Red

1 MRECISIHIG QAGIQVGNAC WELYCLEHGI QADGQMPGDK TIGGGDDAFN
51 TFFSETGAGK HVPR**AVFVDL** **EPTVIDEVR**T GTYR**QLFHPE** **QLISGKEDAA**
101 **NNFAR**GHYTI GKEIVDLCLD RIRKLADNCT GLQGFLVFNA VGGGTGSGLG
151 SLLLERLSVD YGKKSKLGFT VYPSPQVSTS VVEPYNSVLS THSLLEHTDV
201 AILLDNEAIY DICRRSLDIE RPTYTNLNRL VSQVISSLTA SLR**FDGALNV**
251 **DVNEFQTNLV** **PYPR**IHFMLS SYAPVISAEK AYHEQLSVAE ITNSAFEPSS
301 MMAKCDPRHG KYMACCLMYR GDVVPKDVNA AVATIKTKRT IQFVDWCPTG
351 FKCGINYQPP SVVPGGDLAK VQRAVCMISN STSVVEVFSR IDHKFDLMYA
401 KR**AFVHWYVG** **EGMEEGEFSE** **AR**EDLAALEK DYEEVGAEFD EGEDGDEGDE
451 Y

Spot No.: **9**

Uniprot Protein Accession:**B6TIJ2|B6TIJ2_MAIZE**

Plant species: **Zea mays**

Protein name： **Acetylornithine deacetylase**

Peptide sequences: **K.LFHSGLAHK.A; K.YILPDENLQGR.L; R.SNVIVEYPGTVPGR.V; R.LTPFYSTSHVMEK.L; K.WSYPGGGLNQIPGECTISGDVR.L**

PFF Mascot score: **[198]**   Sequence coverage %: **[15]**

Matched peptides No.: **[5]**   p value: **1.4e-015**

Calculated Mr:**49490**   Calculated pI: **5.45**

PFF Searched Score:

Protein score is -10*Log(P), where P is the probability that the observed match is a random event.
Protein scores greater than 62 are significant ($p<0.05$).
Protein scores are derived from ions scores as a non-probabilistic basis for ranking protein hits.

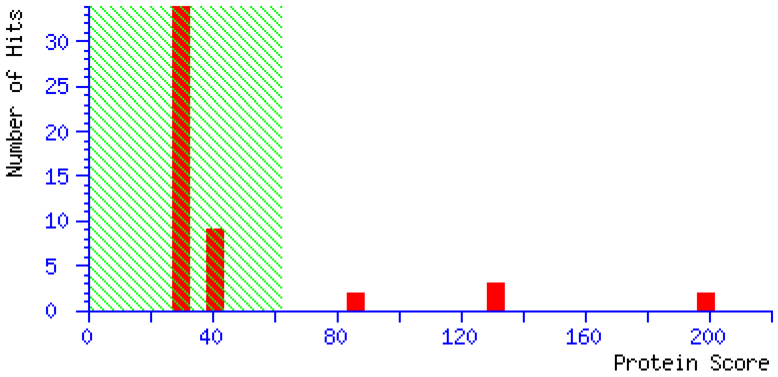

Matched peptide sequences shown in Bold Red

1 MASPASASLK EAVGSLDRDG FVGLLSNLIG ETAHLQNDPP TYRPQEERVA
51 QHVVDVLRPV SADTGGGPLL VRKISYAEGR **SNVIVEYPGT** **VPGR**VVSFVG
101 MHMDVVPANG SEWDFDPFSL TFDSEDKDKL RGRGTTDCLG HVALVAQLMR
151 RLGEVKPPLK HSVIAVFIAN EENSSVTGIG VDGLVKDGLL DKLKTGPLFW
201 IDTADKQPCI GTGGMIPWHL KATGK**LFHSG** **LAHK**AINAME MNMEALKVIQ
251 KRFYTDFPPH EKEKVYKFAT PSTMKPTK**WS** **YPGGGLNQIP** **GECTISGDVR**
301 **LTPFYSTSHV** **MEK**LKEYVED INERFETVLD TRGPVSK**YIL** **PDENLQGR**LE
351 ITFDGDVMNG VACNLESRGY HALCKATKEI VGHVEPYSIT GSLPLIRELQ
401 DEGFDVQTAG YGLLKTYHAK NEYCLFSDMA QGFQVFLSII SQLEEEV

Spot No.: **10**

Uniprot Protein Accession: **Q43697|TBB5_MAIZE**

Plant species: **Zea mays**

Protein name: **Tubulin beta-5 chain**

Peptide sequences: **K.FWEVVCDEHGIDPTGR.Y; R.YVGTSDLQLER.V; R.VNVYYNEASCGR.F; R.AVLMDLEPGTMDSVR.T; K.GHYTEGAELIDSVLDVVR.K; R.FPGQLNSDLR.K; K.SSVCDIPPR.G; R.VSEQFTAMFR.R**

PFF Mascot score: **[308]** Sequence coverage %: **[22]**

Matched peptides No.: **[8]** p value: **1.4e-026**

Calculated Mr: **50695** Calculated *p*I: **4.79**

PFF Searched Score:

Protein score is -10*Log(P), where P is the probability that the observed match is a random event.
Protein scores greater than 62 are significant (p<0.05).
Protein scores are derived from ions scores as a non-probabilistic basis for ranking protein hits.

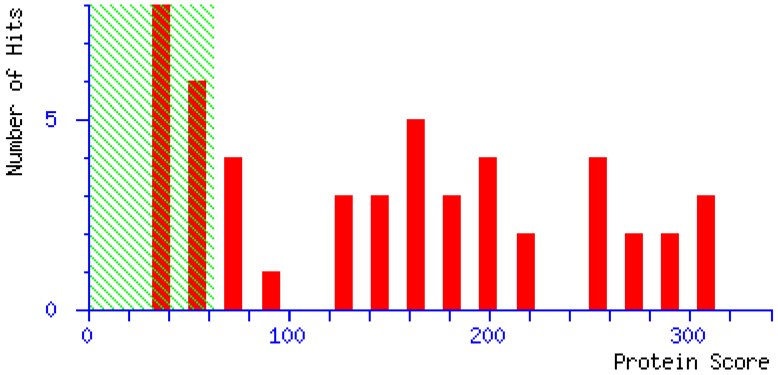

Matched peptide sequences shown in Bold Red

```
  1 MREILHIQGG QCGNQIGSKF WEVVCDEHGI DPTGRYVGTS DLQLERVNVY
 51 YNEASCGRFV PRAVLMDLEP GTMDSVRTGP YGQIFRPDNF VFGQSGAGNN
101 WAKGHYTEGA ELIDSVLDVV RKEAENCDCL QGFQVCHSLG GGTGSGMGTL
151 LISKIREEYP DRMMLTFSVF PSPKVSDTVV EPYNATLSVH QLVENADECM
201 VLDNEALYDI CFRTLKLTTP SFGDLNHLIS ATMSGVTCCL RFPGQLNSDL
251 RKLAVNLIPF PRLHFFMVGF APLTSRGSQQ YRALTVPELT QQMWDAKNMM
301 CAADPRHGRY LTASAMFRGK MSTKEVDEQM INVQNKNSSY FVEWIPNNVK
351 SSVCDIPPRG LSMASTFIGN STSIQEMFRR VSEQFTAMFR RKAFLHWYTG
401 EGMDEMEFTE AESNMNDLVS EYQQYQDATA DEEAEYEDEE AIQDE
```

Spot No.: **11**

Uniprot Protein Accession: **B6T2L2|B6T2L2_MAIZE**

Plant species: **Zea mays**

Protein name: **Sedoheptulose-1,7-bisphosphatase**

Peptide sequences: **K.LLFEALEYSHVCK.Y; K.LTGVTGGDQVAAAMGIYGPR.T; K.DCPGTHEFLLLDEGK.W; K.DTTTIGEGK.M; R.YTGGMVPDVNQIIVK.E; R.VINELDER.T; R.FEETLYGSSR.L**

PFF Mascot score: **[453]** Sequence coverage %: **[23]**

Matched peptides No.: **[7]** p value: **1.4e-041**

Calculated Mr:**42303** Calculated *p*I: **6.08**

PFF Searched Score:

Protein score is -10*Log(P), where P is the probability that the observed match is a random event.
Protein scores greater than 62 are significant ($p<0.05$).
Protein scores are derived from ions scores as a non-probabilistic basis for ranking protein hits.

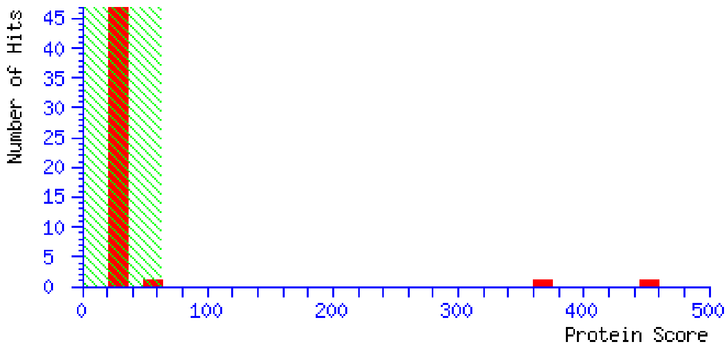

Matched peptide sequences shown in Bold Red

1 MEIVATRSPA CCAAVSFSQS YRPKASRPPT TFYGESVRVN TARPLSARRQ
51 SKAASRAALS ARCEIGDSLE EFLTKATPDK NLIRLLICMG EAMRTIAFKV
101 RTASCGGTAC VNSFGDEQLA VDMLANK**LLF** **EALEYSHVCK** YACSEEVPEL
151 QDMGGPVEGG FSVAFDPLDG SSIVDTNFTV GTIFGVWPGD K**LTGVTGGDQ**
201 **VAAAMGIYGP** **R**TTYIVALK**D** **CPGTHEFLLL** **DEGK**WQHVK**D** **TTTIGEGK**MF
251 SPGNLRATFD NPEYDKLINY YVKEKYTLR**Y** **TGGMVPDVNQ** **IIVK**EKGIFT
301 NVTSPTAKAK LRLLFEVAPL GFLMEKAGGY SSDGKQSVLD R**VINELDER**T
351 QVAYGSKNEI IR**FEETLYGS** **SR**LAASATAT ARALI

Spot No.: **12**

Uniprot Protein Accession: **Q9ZT00|RCA_MAIZE**

Plant species: **Zea mays**

Protein name: **Ribulose bisphosphate carboxylase/oxygenase activase**

Peptide sequences: **K.GLAYDISDDQQDITR.G; K.NFMTLPNIK.V; K.MSCLFINDLDAGAGR.M; R.VPIIVTGNDFSTLYAPLIR.D; K.FYWAPTR.E; R.VYDDEVR.R; R.WVSETGVENIAR.K**

PFF Mascot score: **[402]** Sequence coverage %: **[19]**

Matched peptides No.: **[7]** p value: **5.5e-036**

Calculated Mr: **48079** Calculated *p*I: **6.29**

PFF Searched Score:

Protein score is -10*Log(P), where P is the probability that the observed match is a random event.
Protein scores greater than 62 are significant (p<0.05).
Protein scores are derived from ions scores as a non-probabilistic basis for ranking protein hits.

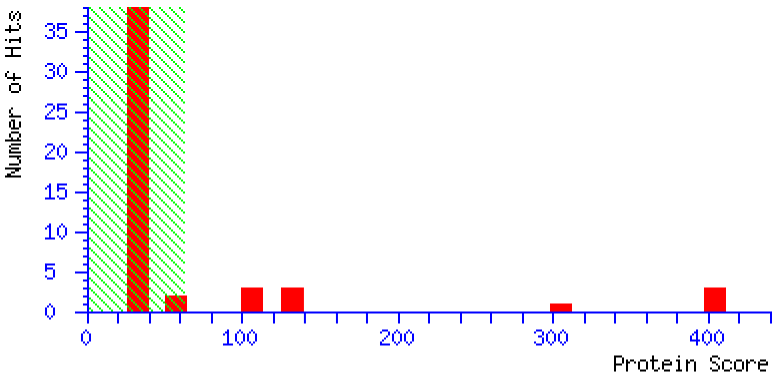

Matched peptide sequences shown in Bold Red

1 MAAAFSSTVG APASTPTRSS FLGKKLNKPQ VSAAVTYHGK SSSSNSRFKA
51 MAAKEVDETK QTDEDRWK**GL** **AYDISDDQQD** **ITR**GKGLVDN LFQAPMGDGT
101 HVAVLSSYDY ISQGQKSYNF DNMMDGFYIA KGFMDKLVVH LSK**NFMTLPN**
151 **IK**VPLILGIW GGKGQGKSFQ CELVFAKMGI TPIMMSAGEL ESGNAGEPAK
201 LIRQRYREAS DLIKKGK**MSC** **LFINDLDAGA** **GR**MGGTTQYT VNNQMVNATL
251 MNIADNPTNV QLPGMYNKED NPR**VPIIVTG** **NDFSTLYAPL** **IR**DGRMEK**FY**
301 **WAPTR**EDRIG VCKGIFRTDG VDEEHVVQLV DTFPGQSIDF FGALRAR**VYD**
351 **DEVR**R**WVSET** **GVENIAR**KLV NSKEGPPTFE QPKITIEKLL EYGHMLVAEQ
401 ENVKRVQLAD KYLNEAALGE ANEDAMKTGS FFK

Spot No.: **13**

Uniprot Protein Accession: **C4J4E4|C4J4E4_MAIZE**

Plant species: **Zea mays**

Protein name: **Uncharacterized protein**

Peptide sequences: **K.GYLFPTNAAR.L; K.LTDFGTQGADSNNILYLR.E; K.MYNELR.R**

PFF Mascot score: **[113]** Sequence coverage %: **[7]**

Matched peptides No.: **[3]** p value: **4.4e-007**

Calculated Mr:**46817** Calculated pI: **5.45**

PFF Searched Score:

Protein score is -10*Log(P), where P is the probability that the observed match is a random event.
Protein scores greater than 62 are significant (p<0.05).
Protein scores are derived from ions scores as a non-probabilistic basis for ranking protein hits.

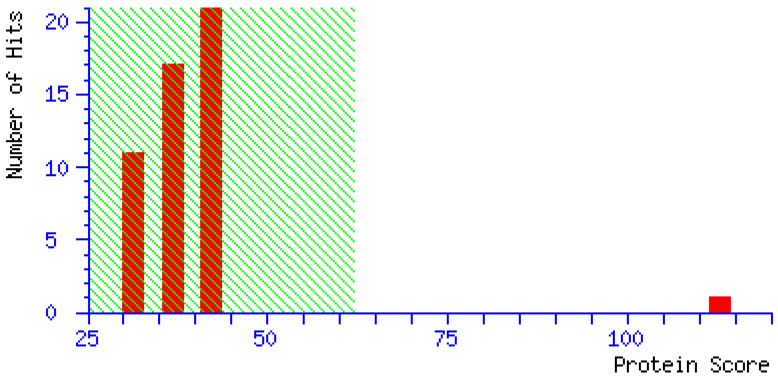

Matched peptide sequences shown in Bold Red

```
  1 MASEKHFKYV ILGGGVAAGY AAREFAKQGV KPGELAIISK EAVAPYERPA
 51 LSKGYLFPTN AARLPGFYVC VGSGGEKLLP EWYSEKGIEL ILSTEIVKAD
101 LSTKTLTSAA GANFTYEILL IATGSSVIKL TDFGTQGADS NNILYLREID
151 DADKLVAAIQ AKKGGKAVVV GGGYIGLELS AALKINDFDV TMVFPEPWCM
201 PRLFTADIAA FYEAYYTNKG VKILKGTLAV GFDADANGDV TAVKLKDGTV
251 LEADIVVVGV GGRPLTTLFK GQVAEEKGGI KTDASFETSV PGVYAIGDVA
301 TFPLKMYNEL RRVEHVDHSR KSAEQAVKAI KGKESGEPVP EYDYLPYFYS
351 RSFDLAWQFY GDNVGETILF GDSDPTSSKP KFGSYWIKDG KVLGAFLEGG
401 SPDENKVIAK VAKTQPPVAN LEELKKDGLQ FASKI
```

Spot No.: **14**

Uniprot Protein Accession: **P00874|RBL_MAIZE**

Plant species: **Zea mays**

Protein name: **Ribulose bisphosphate carboxylase large chain**

Peptide sequences: **K.NHGMHFR.V; R.ACYECLR.G; R.DNGLLLHIHR.A; R.EITLGFVDLLR.D; R.EITLGFVDLLR.D; R.VTPQLGVPPEEAGAAVAAESSTGTWTTVWTDGLTSLDR.Y**

PFF Mascot score: **[426]** Sequence coverage %: **[18]**

Matched peptides No.: **[6]** p value: **2.2e-038**

Calculated Mr: **53295** Calculated pI: **6.33**

PFF Searched Score:

Protein score is -10*Log(P), where P is the probability that the observed match is a random event.
Protein scores greater than 62 are significant (p<0.05).
Protein scores are derived from ions scores as a non-probabilistic basis for ranking protein hits.

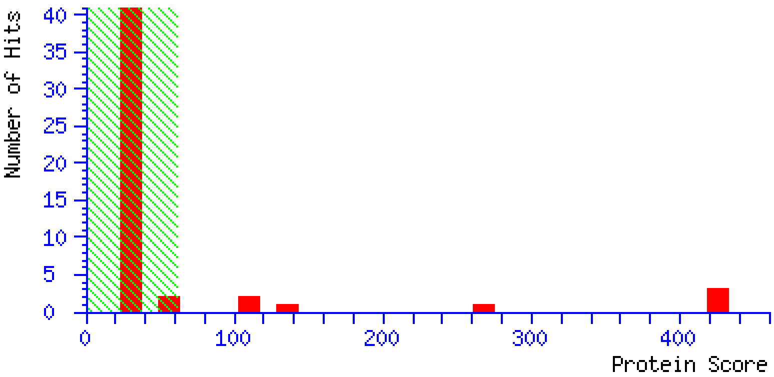

Matched peptide sequences shown in Bold Red

1 MSPQTETKAS VGFKAGVKDY KLTYYTPEYE TKDTDILAAF R**VTPQLGVPP**
51 **EEAGAAVAAE SSTGTWTTVW TDGLTSLDR**Y KGRCYHIEPV PGDPDQYICY
101 VAYPLDLFEE GSVTNMFTSI VGNVFGFKAL RALRLEDLRI PPAYSK**TFQG**
151 **PPHGIQVER**D KLNKYGRPLL GCTIKPKLGL SAKNYGR**ACY ECLR**GGLDFT
201 KDDENVNSQP FMRWRDRFVF CAEAIYKAQA ETGEIKGHYL NATAGTCEEM
251 IKRAVFAREL GVPIVMHDYL TGGFTANTTL SHYCR**DNGLL LHIHR**AMHAV
301 IDRQK**NHGMH FR**VLAKALRM SGGDHIHSGT VVGKLEGER**E ITLGFVDLLR**
351 DDFIEKDRSR GIFFTQDWVS MPGVIPVASG GIHVWHMPAL TEIFGDDSVL
401 QFGGGTLGHP WGNAPGAAAN RVALEACVQA RNEGRDLARE GNEIIKAACK
451 WSAELAAACE IWKEIKFDGF KAMDTI

Spot No.: **15**

Uniprot Protein Accession: **C0PDB0|C0PDB0_MAIZE**

Plant species: **Zea mays**

Protein name: **Phosphoglycerate kinase**

Peptide sequences: **R.ADLNVPLDENQNITDDTR.I; K.FSLAPLVGR.L; K.LVAALPNGGVLLLENVR.F; K.LASLADLYVNDAFGTAHR.A; K.FLQPSVAGFLLQK.E**

PFF Mascot score: **[221]** Sequence coverage %: **[18]**

Matched peptides No.: **[5]** p value: **7e-018**

Calculated Mr: **43227** Calculated *p*I: **6.21**

PFF Searched Score:

Protein score is -10*Log(P), where P is the probability that the observed match is a random event.
Protein scores greater than 62 are significant ($p<0.05$).
Protein scores are derived from ions scores as a non-probabilistic basis for ranking protein hits.

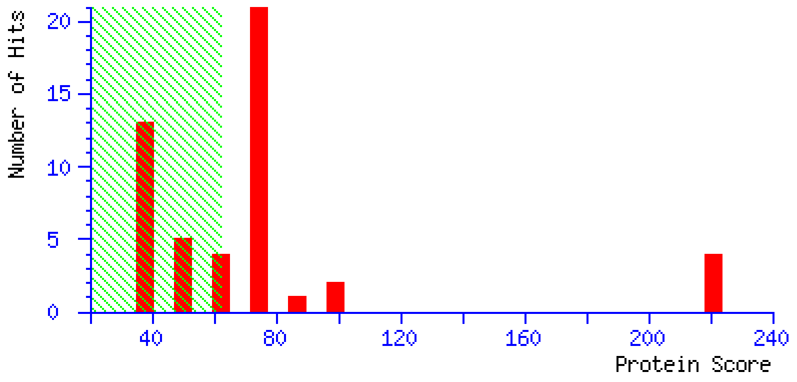

Matched peptide sequences shown in Bold Red

1 MATMAKKSVG ELTEADLEGK RVFVR**ADLNV** **PLDENQNITD** **DTR**IRAAIPT
51 IQYILSKGAK VILSSHLGRP KGFTPK**FSLA** **PLVGR**LSELL GIQVQKADDV
101 IGPEVEK**LVA** **ALPNGGVLLL** **ENVR**FYKEEE KNDPEFAQK**L** **ASLADLYVND**
151 **AFGTAHR**AHA STEGVTK**FLQ** **PSVAGFLLQK** ELDYLVGAVS SPKRPFAAIV
201 GGSKVSSKIG VIESLLEKCD ILLLGGGMIF TFYKAQGLPV GASLVEEDKL
251 ELATSLLAKA KEKGVSLMLP TDVVIADKFA PDANSQIVPA SAIPDGWMGL
301 DIGPDSVASF NAALDTTKTV IWNGPMGVFE FDKFAVGTEA VAKKLAELSG
351 KGVTTIIGGG DSVAAVEKVG VADVMSHIST GGGASLELLE GKELPGVVAL
401 NEAATVTRSK L

Spot No.: **16**

Uniprot Protein Accession: **B6SXW8|B6SXW8_MAIZE**

Plant species: **Zea mays**

Protein name: **RuBisCO large subunit-binding protein subunit alpha**
Peptide sequences: **K.EIAFDQGSR.A; K.LAAAVGVTLGPR.G; K.VVNDGVTIAR.A; K.VGPDGVLSIESSSSFETTVEVEEGMELDR.G; K.EIIPLLEQTTQLR.A**

PFF Mascot score: **[301]**   Sequence coverage %: **[12]**

Matched peptides No.: **[5]**   p value: **7e-026**

Calculated Mr: **61419**   Calculated *p*I: **5.20**

PFF Searched Score:

Protein score is -10*Log(P), where P is the probability that the observed match is a random event.
Protein scores greater than 62 are significant (p<0.05).
Protein scores are derived from ions scores as a non-probabilistic basis for ranking protein hits.

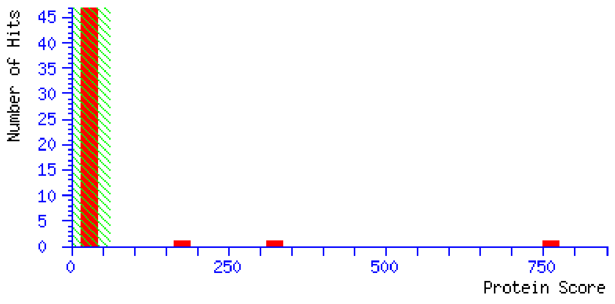

Matched peptide sequences shown in Bold Red

1 MATIPTTDSG LVLSSSALLR RTRRAASSVT ARLPAVARRR PQLLVRASAK
51 **EIAFDQGSR**A ALQAGVEK**LA** **AAVGVTLGPR** GRNVVLDEFG TPK**VVNDGVT**
101 **IAR**AIELADP MENAGASLIR EVASKTNDSA GDGTTTASVL AREIIKLGML
151 SITSGANPVS VKKGIDKTVQ KLVEELEKKS RPVKGSGDIK AVAAISAGND
201 EFVGTMIAEA IDK**VGPDGVL** **SIESSSSFET** **TVEVEEGMEL** **DR**GYISPQFV
251 TNLEKSIVEF ENARILVTDQ KISSIK**EIIP** **LLEQTTQLR**A PLLIIAEDVS
301 GEALATLVIN KLRGILNVAA IKAPGFGERR KALLQDIAIV TGAEYQSKDL
351 GLLVEDTTVE QLGIARKVTI SSSSTTIIAD AASKDDIQAR IAQLKRELSQ
401 TDSTYDSEKL AERIAKLSGG VAVVKVGAST EAELEDRKLR IEDAKNATFA
451 AIEEGIVPGG GAAYVHLSTF VPAIKETLDD PEERLGADII QKALVAPAAL
501 IAHNAGVEGE VIVDKIRESE WEFGYNAMAD KHENLVEAGV IDPAKVTRCA
551 LQNAASVAGM VLTTQAIVVE KPKKAPAAAA AAAP

Spot No.: **17**

Uniprot Protein Accession: **C4J9M7|C4J9M7_MAIZE**

Plant species: **Zea mays**

Protein name: **Post-translational modification, protein turnover, and chaperones**
Peptide sequences: **K.APDFEAEAVFDQEFINVK.L; K.LNTEVLGVSIDSVFSHLAWVQTDR.K; K.AFGVLIPDQGIALR.G; R.GLFIIDKEGVIQHSTINNLAIGR.S**

PFF Mascot score: **[129]** Sequence coverage %: **[27]**

Matched peptides No.: **[4]** p value: **1.10e-8**

Calculated M*r*: **28483** Calculated *p*I: **5.81**

PFF Searched Score:

Protein score is -10*Log(P), where P is the probability that the observed match is a random event.
Protein scores greater than 62 are significant ($p<0.05$).
Protein scores are derived from ions scores as a non-probabilistic basis for ranking protein hits.

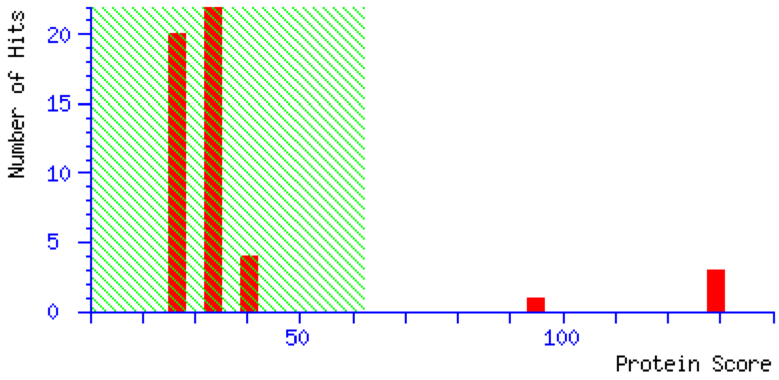

Matched peptide sequences shown in Bold Red

```
  1 MACSFSAAIT VSSAPTPAAR PLAAATQSVC IARSAVATTA RPLRLAASRS
 51 ARATRLVARA SVVDDLPLVG NKAPDFEAEA VFDQEFINVK LSDYIGKKYV
101 ILFFYPLDFT FVCPTEITAF SDRYEEFEKL NTEVLGVSID SVFSHLAWVQ
151 TDRKSGGLGD LKYPLVSDVT KSISKAFGVL IPDQGIALRG LFIIDKEGVI
201 QHSTINNLAI GRSVDETMRT LQALQYVQEN PDEVCPAGWK PGERSMKPDP
251 KGSKEYFAAI
```

Spot No.: **18**

Uniprot Protein Accession: **B6TEW2|B6TEW2_MAIZE**

Plant species: **Zea mays**

Protein name: **Ferredoxin--NADP reductase, leaf isozyme**

Peptide sequences: **R.ITGDQAPGETWHMVFSTEGEVPYR.E; K.DPNATIIMLATGTGIAPFR.S; K.MYIQTR.M**

PFF Mascot score: **[163]** Sequence coverage %: **[14]**

Matched peptides No.: **[3]** p value: **4.4e-012**

Calculated Mr: **37878** Calculated *p*I: **8.37**

PFF Searched Score:

Protein score is -10*Log(P), where P is the probability that the observed match is a random event.
Protein scores greater than 62 are significant (p<0.05).
Protein scores are derived from ions scores as a non-probabilistic basis for ranking protein hits.

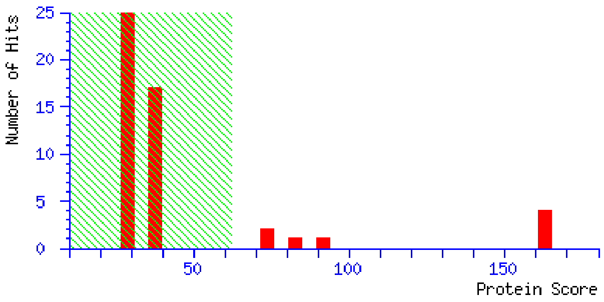

Matched peptide sequences shown in Bold Red

1 MRPPPVPAAA RRPARGAAAR AGVHHRDRGG GAGXKKLEKV SKKQEEGLVT
51 NKYKPKEPYV GRCLLNTR**IT** **GDQAPGETWH** **MVFSTEGEVP** **YR**EGQSIGVI
101 ADGEDKNGKP HKLRLYSIAS SALGDFGDSK TVSLCVKRLV YTNDQGEVVK
151 GVCSNFLCDL KPGAEVKITG PVGKEMLMPK **DPNATIIMLA** **TGTGIAPFR**S
201 FLWKMFFEEH EDYKYTGLAW LFLGVPTSDT LLYKEELEKM KEMAPDNFRL
251 DFAVSREQTN AAGEK**MYIQT** **R**MAEYKEELW ELLKKDNTYV YMCGLKGMEK
301 GIDDIMLDLA AKDGINWLDY KKQLKKSEQW NVEVY

Spot No.: **19**

Uniprot Protein Accession: **B6SRJ5|B6SRJ5_MAIZE**

Plant species: **Zea mays**

Protein name: **Bifunctional 3-phosphoadenosine 5-phosphosulfate synthetase**

Peptide sequences: **K.YNDGLDHYR.L; R.GADAVFAFQLR.N; R.INAGANFYIVGR.D; R.EHEYLQSLHFNCVR.L; R.LAPVDLQWAHVLAEGWASPLR.G**

PFF Mascot score: **[311]** Sequence coverage %: **[14]**

Matched peptides No.: **[5]** p value: **7e-027**

Calculated Mr: **52493** Calculated pI: **8.30**

PFF Searched Score:

Protein score is -10*Log(P), where P is the probability that the observed match is a random event.
Protein scores greater than 62 are significant ($p<0.05$).
Protein scores are derived from ions scores as a non-probabilistic basis for ranking protein hits.

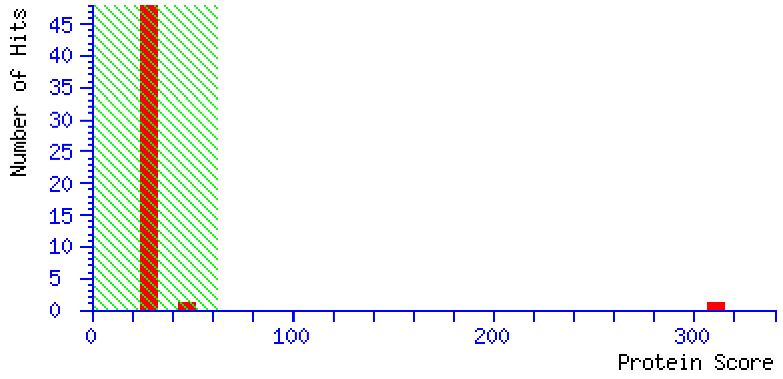

Matched peptide sequences shown in Bold Red

```
  1 MATTHLLTPP RVHHPSPSAS SSVARVRATA SLAHPLHLCR LRLAAPRSRS
 51 PSPRHGRRAM SVRSSLIDPD GGALVELVAP PDRLPALRAE AEALPRVRLA
101 PVDLQWAHVL AEGWASPLRG FMREHEYLQS LHFNCVRLPD GGLVNMSLPI
151 VLAIGDADKE QIGGKPDVAL QGPDGGVVAI LRRVEIYPHN KEERIARTWG
201 TTAPGLPYVD EAIASAGNWL IGGDLEVLEP IKYNDGLDHY RLSPRQLRKE
251 FDKRGADAVF AFQLRNPVHN GHALLMNDTR RRLLEMGYKN PILLLHPLGG
301 YTKADDVPLP VRMEQHSKVL EDGVLDPETT IVSIFPSPMH YAGPTEVQWH
351 AKARINAGAN FYIVGRDPAG MGHPTEKRDL YNPDHGKKVL SMAPGLEKLN
401 ILPFKVAAYD TVAKEMAFFD PSRSQDFLFI SGTKMRTYAK TGENPPDGFM
451 CPGGWKVLVD YYNSLQAEEA TPVPV
```

Spot No.: **20**

Uniprot Protein Accession: **B6SRJ5|B6SRJ5_MAIZE**

Plant species: **Zea mays**

Protein name: **Bifunctional 3-phosphoadenosine 5-phosphosulfate synthetase**

Peptide sequences: **K.YNDGLDHYR.L; R.GADAVFAFQLR.N; R.INAGANFYIVGR.D; R.EHEYLQSLHFNCVR.L; K.TGENPPDGFMCPGGWK.V; R.LAPVDLQWAHVLAEGWASPLR.G**

PFF Mascot score: **[234]** Sequence coverage %: **[17]**

Matched peptides No.: **[6]** p value: **3.5e-019**

Calculated Mr: **52493** Calculated pI: **8.30**

PFF Searched Score:

Protein score is -10*Log(P), where P is the probability that the observed match is a random event.
Protein scores greater than 62 are significant ($p<0.05$).
Protein scores are derived from ions scores as a non-probabilistic basis for ranking protein hits.

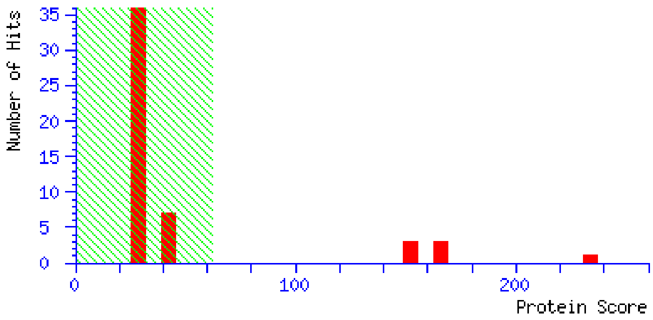

Matched peptide sequences shown in Bold Red

```
  1 MATTHLLTPP RVHHPSPSAS SSVARVRATA SLAHPLHLCR LRLAAPRSRS
 51 PSPRHGRRAM SVRSSLIDPD GGALVELVAP PDRLPALRAE AEALPRVRLA
101 PVDLQWAHVL AEGWASPLRG FMREHEYLQS LHFNCVRLPD GGLVNMSLPI
151 VLAIGDADKE QIGGKPDVAL QGPDGGVVAI LRRVEIYPHN KEERIARTWG
201 TTAPGLPYVD EAIASAGNWL IGGDLEVLEP IKYNDGLDHY RLSPRQLRKE
251 FDKRGADAVF AFQLRNPVHN GHALLMNDTR RRLLEMGYKN PILLLHPLGG
301 YTKADDVPLP VRMEQHSKVL EDGVLDPETT IVSIFPSPMH YAGPTEVQWH
351 AKARINAGAN FYIVGRDPAG MGHPTEKRDL YNPDHGKKVL SMAPGLEKLN
401 ILPFKVAAYD TVAKEMAFFD PSRSQDFLFI SGTKMRTYAK TGENPPDGFM
451 CPGGWKVLVD YYNSLQAEEA TPVPV
```

Spot No.: **21**

Uniprot Protein Accession: **B6T2L2|B6T2L2_MAIZE**

Plant species: **Zea mays**

Protein name: **Sedoheptulose-1,7-bisphosphatase**
Peptide sequences: **K.LLFEALEYSHVCK.Y; K.LTGVTGGDQVAAAMGIYGPR.T; K.DCPGTHEFLLLDEGK.W; K.MFSPGNLR.A; R.YTGGMVPDVNQIIVK.E; K.GIFTNVTSPTAK.A; R.VINELDER.T; R.FEETLYGSSR.L**

PFF Mascot score: **[425]** Sequence coverage %: **[26]**

Matched peptides No.: **[8]** p value: **2.8e-038**

Calculated Mr: **42303** Calculated *p*I: **6.08**

PFF Searched Score:

```
Protein score is -10*Log(P), where P is the probability that the observed match is a random event.
Protein scores greater than 62 are significant (p<0.05).
Protein scores are derived from ions scores as a non-probabilistic basis for ranking protein hits.
```

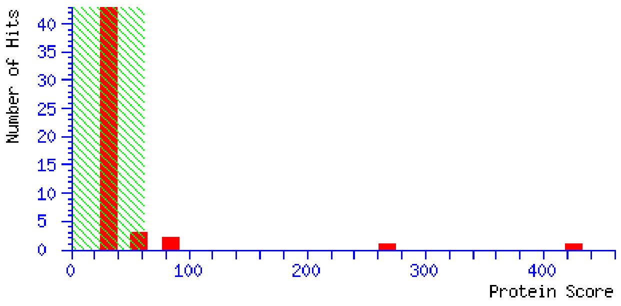

Matched peptide sequences shown in Bold Red

```
  1 MEIVATRSPA CCAAVSFSQS YRPKASRPPT TFYGESVRVN TARPLSARRQ
 51 SKAASRAALS ARCEIGDSLE EFLTKATPDK NLIRLLICMG EAMRTIAFKV
101 RTASCGGTAC VNSFGDEQLA VDMLANKLLF EALEYSHVCK YACSEEVPEL
151 QDMGGPVEGG FSVAFDPLDG SSIVDTNFTV GTIFGVWPGD KLTGVTGGDQ
201 VAAAMGIYGP RTTYIVALKD CPGTHEFLLL DEGKWQHVKD TTTIGEGKMF
251 SPGNLRATFD NPEYDKLINY YVKEKYTLRY TGGMVPDVNQ IIVKEKGIFT
301 NVTSPTAKAK LRLLFEVAPL GFLMEKAGGY SSDGKQSVLD RVINELDERT
351 QVAYGSKNEI IRFEETLYGS SRLAASATAT ARALI
```

Spot No.: **22**

Uniprot Protein Accession: **C0PD30|C0PD30_MAIZE**

Plant species: **Zea mays**

Protein name: **Fructose-bisphosphate aldolase**

Peptide sequences: **K.EAAWGLAR.Y; K.AAQDALLLR.A; R.EAAYYQQGAR.F; R.EAAYYQQGAR.F; K.GLVPLAGSNNESWCQGLDGLASR.E; K.GLVPLAGSNNESWCQGLDGLASR.E; R.TLLVTAPGLGQYISGAILFEETLYQSAVDGR.K**

PFF Mascot score: **[770]** Sequence coverage %: **[34]**

Matched peptides No.: **[7]** p value: **8.8e-073**

Calculated Mr:**38408** Calculated pI: **6.37**

PFF Searched Score:

Protein score is -10*Log(P), where P is the probability that the observed match is a random event.
Protein scores greater than 62 are significant (p<0.05).
Protein scores are derived from ions scores as a non-probabilistic basis for ranking protein hits.

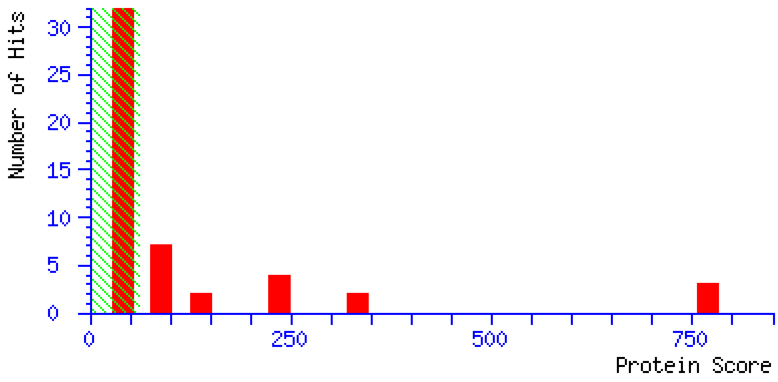

Matched peptide sequences shown in Bold Red

```
  1 MLCIRTYVRM CGQKTIASPG RGILAMDESN ATCGKRLASI GLENTEANRQ
 51 AYRTLLVTAP GLGQYISGAI LFEETLYQSA VDGRKIVDIL AEQGIVPGIK
101 VDKGLVPLAG SNNESWCQGL DGLASREAAY YQQGARFAKW RTVVSIPNGP
151 SELAVKEAAW GLARYAAISQ DNGLVPIVEP EILLDGEHGI ERTFEVAQKV
201 WAETFYAMAE NNVMFEGILL KPSMVTPGAE AKDRATPEQV AAYTLKLLHR
251 RIPPSVPGIM FLSGGQSEVE ATQNLNAMNQ GPNPWHVSFS YARALQNTCL
301 KTWGGQPDKV KAAQDALLLR AKANSLAQLG KYTSDGEAAE AKEGMFVKNY
351 SY
```

Spot No.: **23**

Uniprot Protein Accession: **B6SP64|B6SP64_MAIZE**

Plant species: **Zea mays**

Protein name: **oxygen evolving enhancer protein 3 containing protein**
Peptide sequences: **K.LISAAEDKPPFVDLANR.L**

PFF Mascot score: **[95]** Sequence coverage %: **[7]**

Matched peptides No.: **[1]** p value: **7.30e-07**

Calculated Mr: **25914** Calculated *p*I: **7.66**

PFF Searched Score:

Ions score is -10*Log(P), where P is the probability that the observed match is a random event.
Individual ions scores > 46 indicate identity or extensive homology ($p<0.05$).
Protein scores are derived from ions scores as a non-probabilistic basis for ranking protein hits.

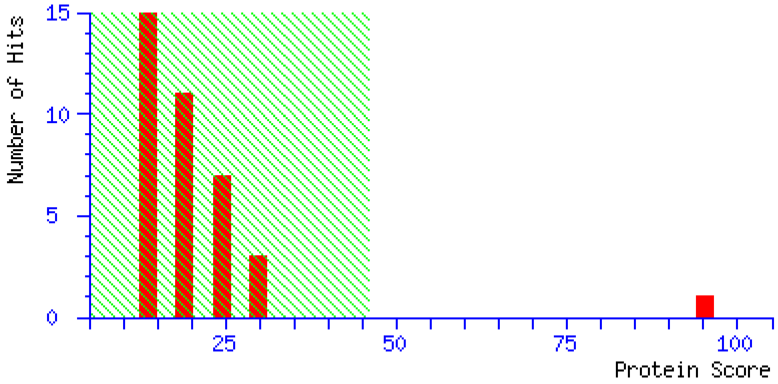

Matched peptide sequences shown in Bold Red

```
  1  MATYLNAPPA ATTTTASCRH AFSGGSLRLL RPSRPTLHVT CQAAVSSSSS
 51  SQPPSSPSRR SACLLGIACA VLLRPRAASA AETDEEPANN GWWLTEFPLP
101  VPKIVNKEIN NAETGTRSFL KNGIYMADIG PSFAAHAYRL RSTAFDLLAL
151  EDLLGKDASN YVNKYLRLKS TFMYYDFDKL ISAAEDKPPF VDLANRLFDS
201  FETLQEAVTA KDDARIGDRY AETKLILQEL MAKMA
```

Spot No.: **24**

Uniprot Protein Accession: **B6UHI4|B6UHI4_MAIZE**

Plant species: **Zea mays**

Protein name: **Putative ATPase, V1 complex, subunit B protein**
Peptide sequences: **K.YQEIVNIR.L; K.TPVSLDMLGR.I; K.IPLFSAAGLPHNEIAAQICR.Q; R.DFEENGSMER.V; R.VTLFLNLANDPTIER.I; R.QIYPPINVLPSLSR.L; K.FVTQGAYDTR.N**

PFF Mascot score: **[285]** Sequence coverage %: **[17]**

Matched peptides No.: **[7]** p value: **2.8e-024**

Calculated Mr: **54169** Calculated *p*I: **5.07**

PFF Searched Score:

Protein score is -10*Log(P), where P is the probability that the observed match is a random event.
Protein scores greater than 62 are significant (p<0.05).
Protein scores are derived from ions scores as a non-probabilistic basis for ranking protein hits.

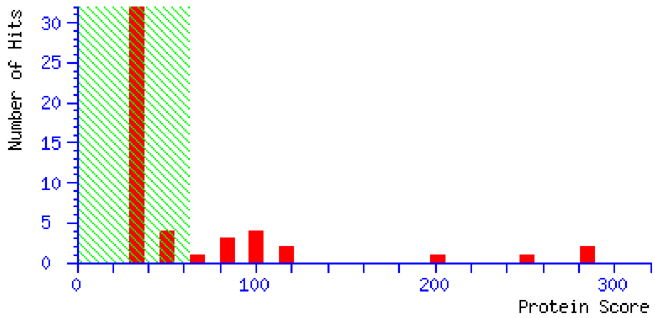

Matched peptide sequences shown in Bold Red

1 MGLVKEGIDM EEGTLEIGME YRTVSGVAGP LVILDKVKGP K**YQEIVNIR**L
51 GDGTNRRGQV LEVDGEKAVV QVFEGTSGID NKYTTVQFTG EVLK**TPVSLD**
101 **MLGR**IFNGSG KPIDNGPPIL PEAYLDISGS SINPSERTYP EEMIQTGIST
151 IDVMNSIARG QK**IPLFSAAG LPHNEIAAQI CR**QAGLVKTL EKGKHAEGGE
201 DDNFAIVFAA MGVNMETAQF FKR**DFEENGS MERVTLFLNL ANDPTIER**II
251 TPRIALTTAE YLAYECGKHV LVILTDMSSY ADALREVSAA REEVPGRRGY
301 PGYMYTDLAT IYERAGRIEG RTGSITQIPI LTMPNDDITH PTPDLTGYIT
351 EGQIYIDRQL HNR**QIYPPIN VLPSLSR**LMK SAIGEGMTRR DHSDVSNQLY
401 ANYAIGKDVQ AMKAVVGEEA LSSEDLLYLE FLDKFERK**FV TQGAYDTR**NI
451 FQSLDLAWTL LRIFPRELLH RIPAKTLDQY YSRDASH

Spot No.: 25

Uniprot Protein Accession: **C0PD30|C0PD30_MAIZE**

Plant species: **Zea mays**

Protein name: **Fructose-bisphosphate aldolase**

Peptide sequences: **K.EAAWGLAR.Y; K.AAQDALLLR.A; R.EAAYYQQGAR.F; R.LASIGLENTEANR.Q; K.GLVPLAGSNNESWCQGLDGLASR.E; R.YAAISQDNGLVPIVEPEILLDGEHGIER.T;R.TLLVTAPGLGQYISGAILFEETLYQSAVDGR.K**

PFF Mascot score: **[883]** Sequence coverage %: **[34]**

Matched peptides No.: **[7]** p value: **4.4e-084**

Calculated Mr:**38408** Calculated pI: **6.37**

PFF Searched Score:

Protein score is -10*Log(P), where P is the probability that the observed match is a random event.
Protein scores greater than 62 are significant ($p<0.05$).
Protein scores are derived from ions scores as a non-probabilistic basis for ranking protein hits.

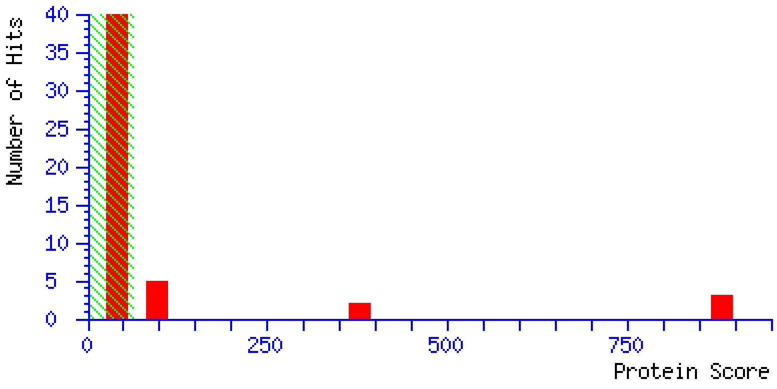

Matched peptide sequences shown in Bold Red

```
  1 MLCIRTYVRM CGQKTIASPG RGILAMDESN ATCGKRLASI GLENTEANRQ
 51 AYRTLLVTAP GLGQYISGAI LFEETLYQSA VDGRKIVDIL AEQGIVPGIK
101 VDKGLVPLAG SNNESWCQGL DGLASREAAY YQQGARFAKW RTVVSIPNGP
151 SELAVKEAAW GLARYAAISQ DNGLVPIVEP EILLDGEHGI ERTFEVAQKV
201 WAETFYAMAE NNVMFEGILL KPSMVTPGAE AKDRATPEQV AAYTLKLLHR
251 RIPPSVPGIM FLSGGQSEVE ATQNLNAMNQ GPNPWHVSFS YARALQNTCL
301 KTWGGQPDKV KAAQDALLLR AKANSLAQLG KYTSDGEAAE AKEGMFVKNY
351 SY
```

Spot No.: **26**

Uniprot Protein Accession: **A0A059Q6M3|A0A059Q6M3_MAIZE**

Plant species: **Zea mays**

Protein name: **ATP synthase subunit alpha, chloroplastic**
Peptide sequences: **R.IIGLGEIMSGELVEFAEGTR.G; R.IAQIPVSEAYLGR.V; R.EAYPGDVFYLHSR.L; K.QSQSNPLPVEEQVATIYTGTR.G**

PFF Mascot score: **[127]** Sequence coverage %: **[13]**

Matched peptides No.: **[4]** p value: **1.7e-008**

Calculated Mr: **55713** Calculated *p*I: **5.87**

PFF Searched Score:

Protein score is -10*Log(P), where P is the probability that the observed match is a random event.
Protein scores greater than 62 are significant (p<0.05).
Protein scores are derived from ions scores as a non-probabilistic basis for ranking protein hits.

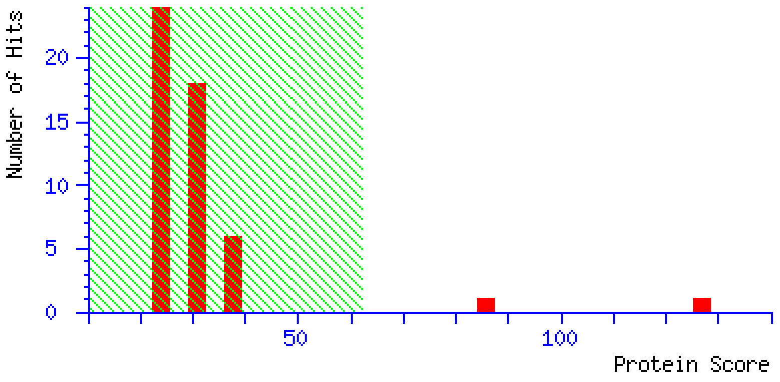

Matched peptide sequences shown in Bold Red

```
  1 MATLRVDEIN KILRERIEQY NRKVGIENIG RVVQVGDGIA RIIGLGEIMS
 51 GELVEFAEGT RGIALNLESK NVGIVLMGDG LMIQEGSFVK ATGRIAQIPV
101 SEAYLGRVIN ALAKPIDGRG EIVASESRLI ESPAPGIISR RSVYEPLQTG
151 LIAIDSMIPI GRGQRELIIG DRQTGKTAVA TDTILNQKGQ DVICVYVAIG
201 QRASSVAQVV TTFHEEGAME YTIVVAEMAD SPATLQYLAP YTGAALAEYF
251 MYRERHTLII YDDLSKQAQA YRQMSLLLRR PPGREAYPGD VFYLHSRLLE
301 RAAKLNSLLG EGSMTALPIV ETQSGDVSAY IPTNVISITD GQIFLSADLF
351 NAGIRPAINV GISVSRVGSA AQIKAMKQVA GKSKLELAQF AELQAFAQFA
401 SALDKTSQNQ LARGRRLREL LKQSQSNPLP VEEQVATIYT GTRGYLDSLE
451 IEQVKKFLDE LRKHLKDTKP QFQEIISSSK TFTEQAETLL KEAIQEQLER
501 FSLQEQT
```

Spot No.: **27**

Uniprot Protein Accession: **B4FU39|B4FU39_MAIZE**

Plant species: **Zea mays**

Protein name: **Uncharacterized protein**

Peptide sequences: **R.DDSGVLSPYSFSR.R; K.HLGVVGLGGLGHVAVK.F; K.AGDTVGVGYFVGSCR.S; R.GGFSDVLVASEHYVVR.V; R.QEAIENLGADEFLISR.D; R.NAMYPVVPGHEIVGVVTGVGGGVTR.F**

PFF Mascot score: **[688]** Sequence coverage %: **[24]**

Matched peptides No.: **[6]** p value: **1.4e-064**

Calculated Mr:**43946** Calculated pI: **7.19**

PFF Searched Score:

Protein score is -10*Log(P), where P is the probability that the observed match is a random event.
Protein scores greater than 62 are significant (p<0.05).
Protein scores are derived from ions scores as a non-probabilistic basis for ranking protein hits.

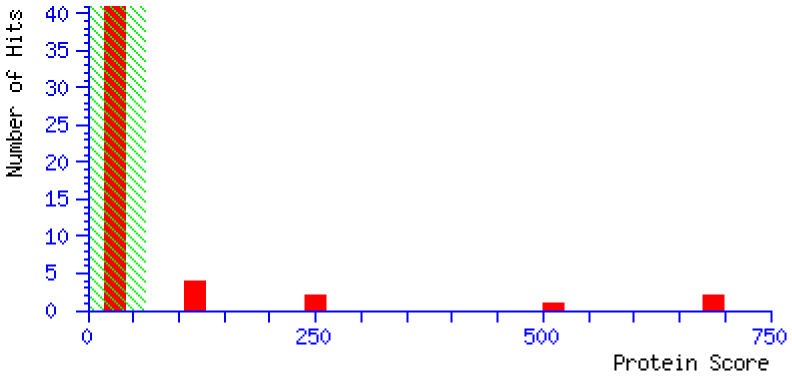

Matched peptide sequences shown in Bold Red

```
  1 MSYHCRVLVP VPFHYPPGAG ARVGAGAGSP FPSASRALRL PRPRASVEKR
 51 EQVAAMEEQG GQAALGWAAR DDSGVLSPYS FSRRVPKDDD VTIKVLYCGI
101 CHTDLHVIKN DWRNAMYPVV PGHEIVGVVT GVGGGVTRFK AGDTVGVGYF
151 VGSCRSCDSC GKGDDNYCAG IVLTSNGVDH AHGGAPTRGG FSDVLVASEH
201 YVVRVPDGLA LDRTAPLLCA GVTVYSPMMR HGLNEPGKHL GVVGLGGLGH
251 VAVKFGKAFG MKVTVISTSA SKRQEAIENL GADEFLISRD EDQMKAATGT
301 MDGIIDTVSA WHPITPLLAL LKPLGQMVVV GAPSKPLELP AYAIVPGGKG
351 VAGNNVGSVR DCQAMLEFAG KHGIGAEVEV IKMDYVNTAM ERLEKNDVRY
401 RFVIDVAGSL GSAA
```

Spot No.: **28**

Uniprot Protein Accession: **B6UHI4|B6UHI4_MAIZE**

Plant species: **Zea mays**

Protein name: **Filamentation temperature-sensitive H 2B**
Peptide sequences: **R.AQGGLGGPNGPGFPLGFGQSR.A; K.ENAPCIVFVDEIDAVGR.Q; K.QLSDEAYEIALR.H; R.AILSEFAEIPVENR.V.**

PFF Mascot score: **[203]** Sequence coverage %: **[9]**

Matched peptides No.: **[4]** p value: **4.4e-016**

Calculated Mr: **72611** Calculated *p*I: **5.69**

PFF Searched Score:

Protein score is -10*Log(P), where P is the probability that the observed match is a random event.
Protein scores greater than 62 are significant (p<0.05).
Protein scores are derived from ions scores as a non-probabilistic basis for ranking protein hits.

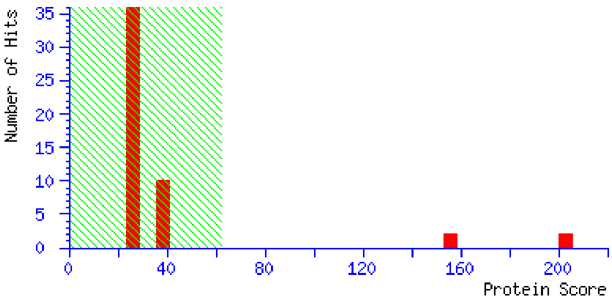

Matched peptide sequences shown in Bold Red

1 MALPSMSLVA KGVLPFSART SSGVTQRPVS VTASLEHKTS DARRKFLKLA
51 LGNLGVGLPT LLGAKKALAD EQGVSSSRMS YSRFLEYLDK DRVKKVDLFE
101 NGTIAIVEAI SPELGNRVQR VRVQLPGLSQ ELLQKLREKN IDFAAHSNQE
151 DSGSLLFNLI GNLAFPLILI GGLFLLSRR**A** **QGGLGGPNGP** **GFPLGFGQSR**
201 AKFQMEPNTG VTFDDVAGVD EAKQDFMEVV EFLKKPERFT AVGARIPKGV
251 LLVGPPGTGK TLLAKAIAGE AGVPFFSISG SEFVEMFVGV GASRVRDLFK
301 KAK**ENAPCIV** **FVDEIDAVGR** QRGTGIGGGN DEREQTLNQL LTEMDGFEGN
351 TGIIVIAATN RADILDSALL RPGRFDRQVS VDVPDVRGRT EILKVHGSNK
401 KFDSDVSLDV IAMRTPGFSG ADLANLLNEA AILAGRRGRT AISSKEIDDS
451 IDRIVAGMEG TVMTDGKSKS LVAYHEVGHA ICGTLTPGHD PVQKVTLVPR
501 GQARGLTWFI PMDDPTLISR QQLFARIVGG LGGRAAEEVI FGEPEVTTGA
551 AGDLQQITGL AKQMVVTFGM SEIGPWSLME GGAQSGDVIM RMMARNSMSE
601 KLAEDIDSAV K**QLSDEAYEI** **ALR**HIRNNRE AIDKIVEVLI EKETVTGDEF
651 R**AILSEFAEI** **PVENR**VPPAT PAAALPA

Spot No.: **29**

Uniprot Protein Accession: **C4J1J9|C4J1J9_MAIZE**

Plant species: **Zea mays**

Protein name: **Elongation factor Ts**

Peptide sequences: **R.VGSYIHDSR.I; R.LAAEGLVGSYIHDSR.I**

PFF Mascot score: **[80]** Sequence coverage %: **[5]**

Matched peptides No.: **[2]** p value: **0.00092**

Calculated Mr: **47103** Calculated *p*I: **5.44**

PFF Searched Score:

Protein score is -10*Log(P), where P is the probability that the observed match is a random event.
Protein scores greater than 62 are significant (p<0.05).
Protein scores are derived from ions scores as a non-probabilistic basis for ranking protein hits.

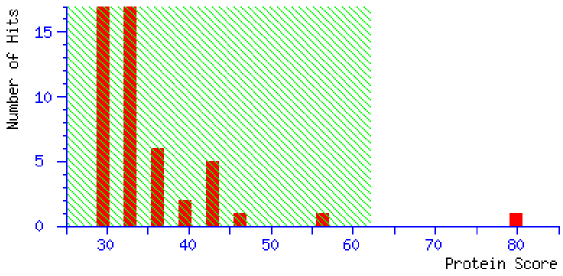

Matched peptide sequences shown in Bold Red

```
  1 MMDCKKALAE TGGDIDKAQE FLRKKGLAAA DKRAGRATAE GRVGSYIHDS
 51 RIGVLIEVNC ETDFVSRGDI FKELVDDLAM QIAACPQVQY ISIDDVPEEL
101 VKKETELEMQ REDLLSKPEQ IRAKIVEGRV KKRLGEFALF EQPFIKNDKV
151 TISEWLKQTI ATTGENMKVR RFARYNLGEG LEKKSQDFAA EVAAQTAAKP
201 PPSAPPKEDK PAETTESAEK KPAVAVSASL VKQLRDETGA GMMDCKKALA
251 ESDGDLQKAQ EFLRKKGLSS ADKKSSRLAA EGLVGSYIHD SRIGCMIEVN
301 SETDFVARND KFRELVNDLA MQVVACPQVD YVSVDDIPES VVGKEKEIEM
351 QREDLQSKPE SIREKIVEGR IAKRLGVMAL LEQPYIKDDS KTVKDLVKET
401 VASLGENIKV RRFVRYTLGE D
```

Spot No.: **30**

Uniprot Protein Accession: **B6T2L2|B6T2L2_MAIZE**

Plant species: **Zea mays**

Protein name： **Sedoheptulose-1,7-bisphosphatase**

Peptide sequences: **K.LLFEALEYSHVCK.Y； K.LTGVTGGDQVAAAMGIYGPR.T； K.DCPGTHEFLLLDEGK.W； K.MFSPGNLR.A； R.YTGGMVPDVNQIIVK.E； K.GIFTNVTSPTAK.A； R.VINELDER.T； R.FEETLYGSSR.L**

PFF Mascot score: **[571]** Sequence coverage %: **[26]**

Matched peptides No.: **[8]** p value: **7e-053**

Calculated Mr: **42303** Calculated *p*I: **6.08**

PFF Searched Score:

Protein score is -10*Log(P), where P is the probability that the observed match is a random event.
Protein scores greater than 62 are significant (p<0.05).
Protein scores are derived from ions scores as a non-probabilistic basis for ranking protein hits.

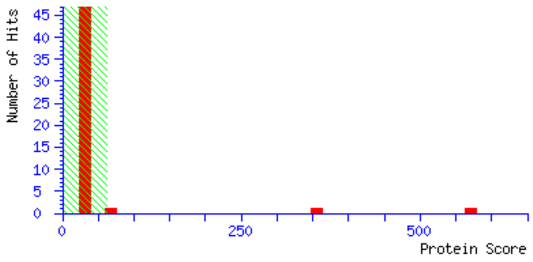

Matched peptide sequences shown in Bold Red

```
  1 MEIVATRSPA CCAAVSFSQS YRPKASRPPT TFYGESVRVN TARPLSARRQ
 51 SKAASRAALS ARCEIGDSLE EFLTKATPDK NLIRLLICMG EAMRTIAFKV
101 RTASCGGTAC VNSFGDEQLA VDMLANKLLF EALEYSHVCK YACSEEVPEL
151 QDMGGPVEGG FSVAFDPLDG SSIVDTNFTV GTIFGVWPGD KLTGVTGGDQ
201 VAAAMGIYGP RTTYIVALKD CPGTHEFLLL DEGKWQHVKD TTTIGEGKMF
251 SPGNLRATFD NPEYDKLINY YVKEKYTLRY TGGMVPDVNQ IIVKEKGIFT
301 NVTSPTAKAK LRLLFEVAPL GFLMEKAGGY SSDGKQSVLD RVINELDERT
351 QVAYGSKNEI IRFEETLYGS SRLAASATAT ARALI
```

Spot No.: 31

Uniprot Protein Accession: **C0P530|C0P530_MAIZE**

Plant species: **Zea mays**

Protein name: **Putative TCP-1/cpn60 chaperonin family protein isoform 1**

Peptide sequences: **K.IVNDGVTVAR.E; K.VVAAGANPVQITR.G; K.ALVEELR.K; K.AAVEEGIVVGGGCTLLR.L**

PFF Mascot score: **[157]** Sequence coverage %: **[8]**

Matched peptides No.: **[4]** p value: 1.**7e-011**

Calculated Mr: **61969** Calculated *p*I: **5.42**

PFF Searched Score:

Protein score is -10*Log(P), where P is the probability that the observed match is a random event.
Protein scores greater than 62 are significant (p<0.05).
Protein scores are derived from ions scores as a non-probabilistic basis for ranking protein hits.

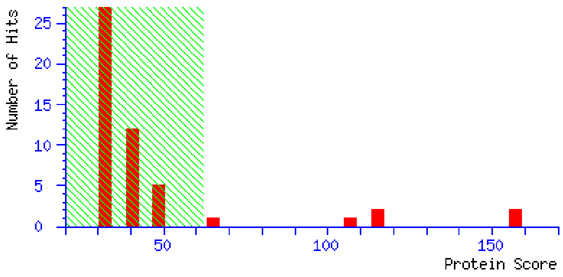

Matched peptide sequences shown in Bold Red

1 MASTFGATST VGLMAAPTGK NVRLQRRANF RVKAAKELYF NKDGSAIKKL
51 QTGVNKLADL VGVTLGPKGR NVVLESKYGS PK**IVNDGVTV** **AR**EVELEDPV
101 ENIGAKLVRQ AAAKTNDLAG DGTTTSVVLA QGLIAEGVK**V** **VAAGANPVQI**
151 **TR**GIEKTAK**A** **LVEELR**KLSK EVEDSELADV AAVSAGNNYE IGNMIAEAMS
201 KVGRKGVVTL EEGKSSENFL YVVEGMQFDR GYISPYFVTD SEKMTAEYEN
251 CKLLLVDKKI TNARDLINVL EEAIRGAYPI LIIAEDIEQE ALATLVVNKL
301 RGSLKIAAIK APGFGERKTQ YLDDIAILTG ATVIRDEVGL SLDKADKSVL
351 GTAAKVVLTK ESTTIVGDGS TQEEVTKRVA QIKNLIEAAE QEYEKEKLNE
401 RIAKLAGGVA VIQVGAQTET ELKEKKLRVE DALNATK**AAV** **EEGIVVGGGC**
451 **TLLR**LAAKVD AIKDTLENDE QKVGAEIVRR ALSYPLKLIA KNAGVNGSVV
501 TEKVLSNDNF KYGYNAATGQ YEDLMAAGII DPTKVVRCCL EHAASVAKTF
551 LTSDVVVVDI KEPEAAPVAN PMDNSGYGY

Spot No.: **32**

Uniprot Protein Accession: **A0A096SD19|A0A096SD19_MAIZE**

Plant species: **Zea mays**

Protein name: **Uncharacterized protein**

Peptide sequences: **K.GQEELEATSIELASIAEAR.D; K.NLESTTHELVEER.K.**

PFF Mascot score: **[80]** Sequence coverage %: **[5]**

Matched peptides No.: **[2]** p value: **0.00088**

Calculated Mr: **67864** Calculated *p*I: **4.90**

PFF Searched Score:

Protein score is -10*Log(P), where P is the probability that the observed match is a random event.
Protein scores greater than 62 are significant (p<0.05).
Protein scores are derived from ions scores as a non-probabilistic basis for ranking protein hits.

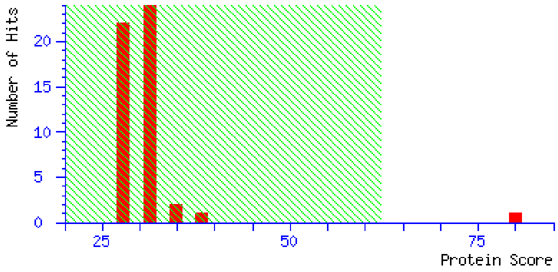

Matched peptide sequences shown in Bold Red

```
  1 MEIGDLIYSM FYLQMESKLA ENEAALSLMR DNYEKRLLEQ QAAQKKQSMK
 51 FQEQEVSLSG QLASATKTLT SLSEEFRKEK KLAEELRDEI QRLESSITQA
101 GIDNDVLETK LEEKLGEINF LQEKVSLLNQ EIDDKEKHIR ELSASLSSKE
151 VDYQKLTAFT NQTKKSLELA NSRVQQLEEE LSTTKNALVS KISSIDSLNA
201 KLETLNSEKE EADKKINELI QEYTDLKVAS ETRASHDSKL LSERDDLIKQ
251 LEEKLSVALT DSSKDQETIV ELNKELDATK MMLKNELKSM EALKDSIRSS
301 EEALKTSRSE VSKLSKELEE ANELNEDLVS QISKLREESN EMQVDLTNKL
351 GEAESLSKAL SEDLASVNEM VQKGQEELEA TSIELASIAE ARDNLKKELL
401 DAYKNLESTT HELVEERKIV TALNKELEAL AKQLQVDSEA RKSLESDLEE
451 ATKSLDEMNN SALLSKELE STHSRSATLE SEKEMLRKAL AEQTKITTEA
501 KENTEDAQNL ITRLETEKES FELRCRHLEE ELALAKGEIL RLRRQISTNS
551 SQKPRARGPP EASETLKEQP VNDYNQKTSG VVAGTPQPVK RTVRRRKGGA
601
```

Spot No.: **33**

Uniprot Protein Accession: **Q6LBU9|Q6LBU9_MAIZE**

Plant species: **Zea mays**

Protein name: **Glyceraldehyde-3-phosphate dehydrogenase (Fragment)**

Peptide sequences: **R.LLDASHR.D; K.TLAEEVNQAFR.D; K.VISWYDNEWGYSQR.V; R.DAAANELTGILEVCDVPLVSVDFR.C;**

PFF Mascot score: **[408]** Sequence coverage %: **[14]**

Matched peptides No.: **[4]** p value: **1.4e-036**

Calculated Mr:**41265** Calculated pI: **7.21**

PFF Searched Score:

Protein score is -10*Log(P), where P is the probability that the observed match is a random event.
Protein scores greater than 62 are significant (p<0.05).
Protein scores are derived from ions scores as a non-probabilistic basis for ranking protein hits.

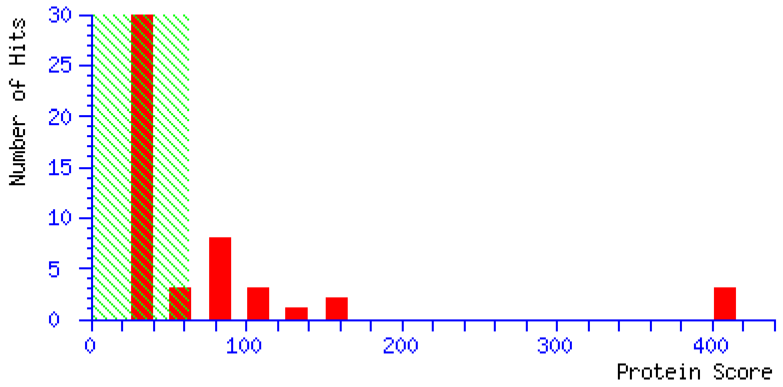

Matched peptide sequences shown in Bold Red

```
  1 EFSGLRSSAS LPMRRNATSD DFMSAVSFRT HAVGTSGGPR RAPTEAKLKV
 51 AINGFGRIGR NFLRCWHGRG DASPLDVIAI NDTGGVKQAS HLLKYDSTLG
101 IFDADVKPVG DNAISVDGKV IKVVSDRNPS NLPWGELGID LVIEGTGVFV
151 DREGAGKHIQ AGAKKVLITA PGKGDIPTYV VGVNADQYNP DEPIISNASC
201 TTNCLAPFVK VLDQKFGIIK GTMTTTHSYT GDQRLLDASH RDLRRARAAA
251 LNIVPTSTGA AKAVSLVLPN LKGKLNGIAL RVPTPNVSVV DLVVQVSKKT
301 LAEEVNQAFR DAAANELTGI LEVCDVPLVS VDFRCSDVSS TIDASLTMVM
351 GDDMVKVISW YDNEWGYSQR VVDLADICAN QWK
```

Spot No.: **34**

Uniprot Protein Accession: **C0PDB0|C0PDB0_MAIZE**

Plant species: **Zea mays**

Protein name: **Phosphoglycerate kinase**

Peptide sequences: **R.ADLNVPLDENQNITDDTR.I ; K.VILSSHLGR.P; K.FSLAPLVGR.L; K.LVAALPNGGVLLLENVR.F; K.LASLADLYVNDAFGTAHR.A; K.FLQPSVAGFLLQK.E.**

PFF Mascot score: **[596]** Sequence coverage %: **[20]**

Matched peptides No.: **[6]** p value: **2.2e-055**

Calculated Mr: **43227** Calculated *p*I: **5.21**

PFF Searched Score:

Protein score is -10*Log(P), where P is the probability that the observed match is a random event.
Protein scores greater than 62 are significant (p<0.05).
Protein scores are derived from ions scores as a non-probabilistic basis for ranking protein hits.

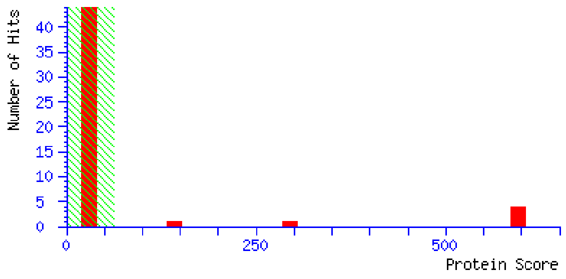

Matched peptide sequences shown in Bold Red

1 MATMAKKSVG ELTEADLEGK RVFVR**ADLNV PLDENQNITD DTR**IRAAIPT

51 IQYILSKGAK **VILSSHLGR**P KGFTPK**FSLA PLVGR**LSELL GIQVQKADDV

101 IGPEVEK**LVA ALPNGGVLLL ENVR**FYKEEE KNDPEFAQK**L ASLADLYVND**

151 **AFGTAHR**AHA STEGVTK**FLQ PSVAGFLLQK** ELDYLVGAVS SPKRPFAAIV

201 GGSKVSSKIG VIESLLEKCD ILLLGGGMIF TFYKAQGLPV GASLVEEDKL

251 ELATSLLAKA KEKGVSLMLP TDVVIADKFA PDANSQIVPA SAIPDGWMGL

301 DIGPDSVASF NAALDTTKTV IWNGPMGVFE FDKFAVGTEA VAKKLAELSG

351 KGVTTIIGGG DSVAAVEKVG VADVMSHIST GGGASLELLE GKELPGVVAL

401 NEAATVTRSK L

Spot No.: **35**

Uniprot Protein Accession: **C0P7R5|C0P7R5_MAIZE**

Plant species: **Zea mays**

Protein name: **Elongation factor Tu**

Peptide sequences: **R.PQFYMR.T; R.HSPFFPGYR.P; K.IGDTVDIVGIR.D; K.LVDSVDSYIPVPQR.Q; R.HYAHVDCPGHADYVK.N; K.MVVQLIQPVACEQGMR.F; K.DMVDDEELLELVELEVR.E; R.QTDLPFLLAVEDVFSITGR.G**

PFF Mascot score: **[637]** Sequence coverage %: **[28]**

Matched peptides No.: **[8]** p value: **1.7e-059**

Calculated Mr: **41470** Calculated pI: **4.91**

PFF Searched Score:

Protein score is -10*Log(P), where P is the probability that the observed match is a random event.
Protein scores greater than 62 are significant ($p<0.05$).
Protein scores are derived from ions scores as a non-probabilistic basis for ranking protein hits.

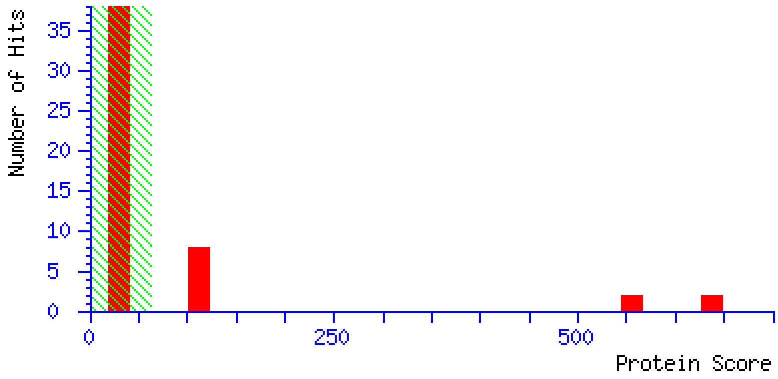

Matched peptide sequences shown in Bold Red

```
  1 MVLASVGGSA PKKYDEIDAA PEERARGITI NTATVEYETE TRHYAHVDCP
 51 GHADYVKNMI TGAAQMDGAI LVVSGADGPM PQTKEHILLA KQVGVPKIVV
101 FLNKKDMVDD EELLELVELE VRELLSNYEY DGDEVPIVAG SALKALEALM
151 GNPTLKRGDD EWVDCIFKLV DSVDSYIPVP QRQTDLPFLL AVEDVFSITG
201 RGTVATGRIE RGTVKIGDTV DIVGIRDTRN CTVTGVEMFQ KTMDDAMAGD
251 NVGLLLRGMQ KDDIERGMVL AKPGSITPHT KFEAVVYVLK KEEGGRHSPF
301 FPGYRPQFYM RTTDVTGNVT VIMNDKDEEA KMCMPGDRIK MVVQLIQPVA
351 CEQGMRFAIR EGGKTVGAGV INKIIE
```

Spot No.: **36**

Uniprot Protein Accession: **B4FCE2|B4FCE2_MAIZE**

Plant species: **Zea mays**

Protein name： **Triosephosphate isomerase**

Peptide sequences: **K.GGAYTGEISAEQLVDIGCQWVILGHSER.R; K.TFEVCFEQMK.A; K.VATPEQAQEVHAAVR.D; R.DWLTTNISPDVASSTR.I**

PFF Mascot score: **[157]** Sequence coverage %: **[24]**

Matched peptides No.: **[4]** p value: **1.7e-011**

Calculated Mr: **30866** Calculated *p*I: **6.91**

PFF Searched Score:

Protein score is -10*Log(P), where P is the probability that the observed match is a random event.
Protein scores greater than 62 are significant (p<0.05).
Protein scores are derived from ions scores as a non-probabilistic basis for ranking protein hits.

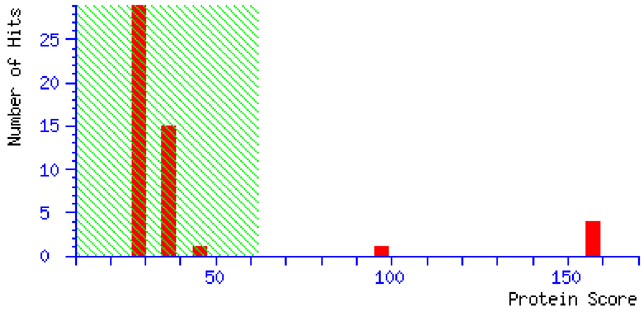

Matched peptide sequences shown in Bold Red

1 MAAAPSSLVS SHLSRLADLR RAAAPATPTV PQQLRVGFSR RRAQRVVAMA
51 GSGKFFVGGN WKCNGTKDSV SKLVSELNAA TLETDVDVVV APPFIYIDQV
101 KNSLTGRIEV SAQNVWIGK**G GAYTGEISAE QLVDIGCQWV ILGHSER**RHI
151 IGENDEFIGK KAAYALSQNV KVIACIGELL EEREAGK**TFE VCFEQMK**AFA
201 DSISNWADVV IAYEPVWAIG TGK**VATPEQA QEVHAAVRDW LTTNISPDVA**
251 **SSTR**IIYGGS VNAANCAELA RKRISMVFLL VVPP

Spot No.: **37**

Uniprot Protein Accession: **B6SLW1|B6SLW1_MAIZE**

Plant species: **Zea mays**

Protein name: **Calmodulin**

Peptide sequences: **-.MATVPTWPPPFSSLLGSLISR.P; K.ELGTVMR.S; K.LTDEEVDEMIR.E**

PFF Mascot score: **[62]** Sequence coverage %: **[23]**

Matched peptides No.: **[3]** p value: **0.053**

Calculated Mr:**18877** Calculated *p*I: **4.44**

PFF Searched Score:

Protein score is -10*Log(P), where P is the probability that the observed match is a random event.
Protein scores greater than 62 are significant (p<0.05).
Protein scores are derived from ions scores as a non-probabilistic basis for ranking protein hits.

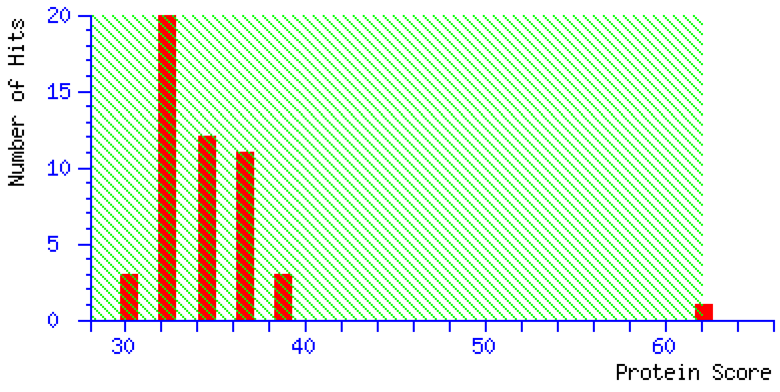

Matched peptide sequences shown in Bold Red

```
  1 MATVPTWPPP FSSLLGSLIS RPDRDRAPVP PRYSTNCGVR VGAVAGCITT
 51 KELGTVMRSL GQNPTEAELQ DMINEVDADG NGTIDFPEFL NLMARKMKDT
101 DSEEELKEAF RVFDKDQNGF ISAAELRHVM TNLGEKLTDE EVDEMIREAD
151 VDGDGQINYE EFVKVMMAK
```

Spot No.: **38**

Uniprot Protein Accession: **B4FRG1|B4FRG1_MAIZE**

Plant species: **Zea mays**

Protein name: **14-3-3-like protein**

Peptide sequences: **R.SAGGAGGGEELSVEER.N; R.GNEAHAASIR.A; K.AAQDIALADLAPTHPIR.L**

PFF Mascot score: **[125]**   Sequence coverage %: **[16]**

Matched peptides No.: **[3]**   p value: **2.8e-008**

Calculated Mr:**29411**   Calculated *p*I: **4.80**

PFF Searched Score:

Protein score is -10*Log(P), where P is the probability that the observed match is a random event.
Protein scores greater than 62 are significant (p<0.05).
Protein scores are derived from ions scores as a non-probabilistic basis for ranking protein hits.

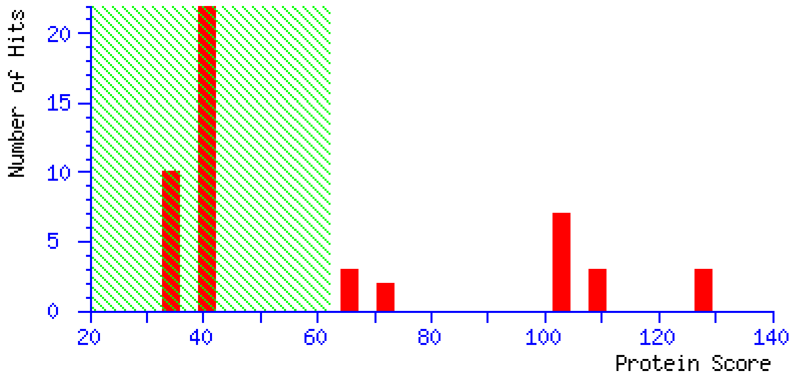

Matched peptide sequences shown in Bold Red

```
  1 MSPSEPTREE SVYMAKLAEQ AERYEEMVEF MERVARSAGG AGGGEELSVE
 51 ERNLLSVAYK NVIGARRASW RIISSIEQKE EGRGNEAHAA SIRAYRSKIE
101 TELARICDGI LALLDSHLVP SAGGAESKVF YLKMKGDYHR YLAEFKSGAE
151 RKDAAESTMN AYKAAQDIAL ADLAPTHPIR LGLALNFSVF YYEILNSPDR
201 ACNLAKQAFD EAISELDSLG EESYKDSTLI MQLLRDNLTL WTSDTNEDGG
251 DEIKDAAAPK ESAEGQ
```

Spot No.: **39**

Uniprot Protein Accession: **B6T3B2|B6T3B2_MAIZE**

Plant species: **Zea mays**

Protein name: **Oxygen-evolving enhancer protein 1**

Peptide sequences: **R.LTYTLDEIEGPLEVGSDGTLK.F; K.DGIDYAAVTVQLPGGER.V; R.GGSTGYDNAVALPAGGR.G**

PFF Mascot score: **[129]** Sequence coverage %: **[16]**

Matched peptides No.: **[3]** p value: **1.1e-008**

Calculated Mr: **34783** Calculated *p*I: **5.59**

PFF Searched Score:

Protein score is -10*Log(P), where P is the probability that the observed match is a random event.
Protein scores greater than 62 are significant (p<0.05).
Protein scores are derived from ions scores as a non-probabilistic basis for ranking protein hits.

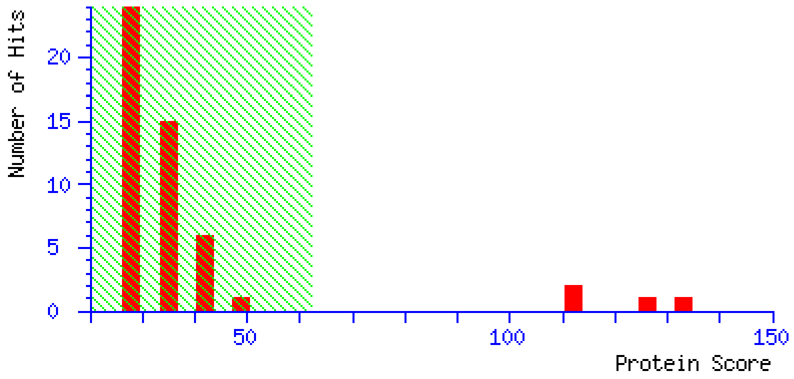

Matched peptide sequences shown in Bold Red

```
  1 MAASLQAAAT LMQPAKIGGR APFASLPSRP SSQLACRAFG VDAGAARITC
 51 SLQDVASRCV DAAKLAGFAL ATSALLVSGA SAEGTPKRLT YDEIQSKTYM
101 EVKGTGTANQ CPTIDGGVES FPFKAGKYQM KKLCLEPTSF TVKAEGIAKN
151 APPEFQKTKL MTRLTYTLDE IEGPLEVGSD GTLKFEEKDG IDYAAVTVQL
201 PGGERVPFLF TVKQLVATGK PESFGGPFLV PSYRGSSFLD PKGRGGSTGY
251 DNAVALPAGG RGDEEELQKE NIKNAASSTG NITLSVTKSN PETGEVIGVF
301 ESVQPSDTDL GAKAPKDVKI QGVWYAQLE
```

Spot No.: **40**

Uniprot Protein Accession: **B8A068|B8A068_MAIZE**

Plant species: **Zea mays**

Protein name: **S-adenosylmethionine synthase**

Peptide sequences: **K.SIVAAGLAR.R; K.TAAYGHFGR.D; R.NEGGAMVPIR.V; K.TIFHLNPSGR.F; R.FVIGGPHGDAGLTGR.K; R.NIGFVSNDVGLDADHCK.V; K.VLVNIEQQSPDIAQGVHGHFTK.R; R.VHTVLISTQHDETVTNDEIAADLK.E**

PFF Mascot score: **[716]**   Sequence coverage %: **[29]**

Matched peptides No.: **[8]**   p value: **2.7e-067**

Calculated Mr:**43445**   Calculated pI: **5.57**

PFF Searched Score:

Protein score is -10*Log(P), where P is the probability that the observed match is a random event.
Protein scores greater than 62 are significant (p<0.05).
Protein scores are derived from ions scores as a non-probabilistic basis for ranking protein hits.

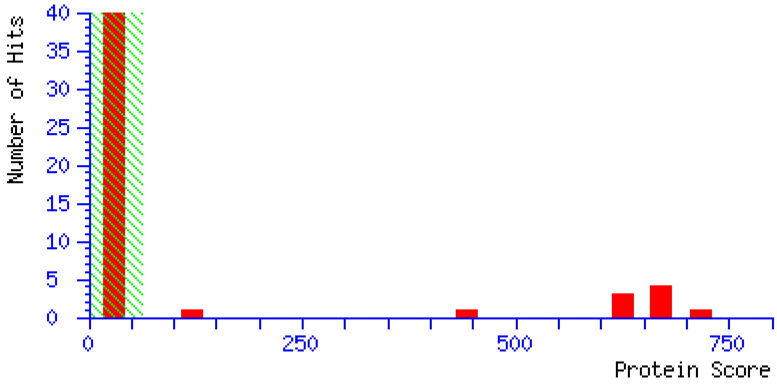

Matched peptide sequences shown in Bold Red

```
  1 MAGLDTFLFT SESVNEGHPD KLCDQVSDAV LDACLAEDPD SKVACETCTK
 51 TNMVMVFGEI TTKANVDYEK IVRETCRNIG FVSNDVGLDA DHCKVLVNIE
101 QQSPDIAQGV HGHFTKRPEE IGAGDQGHMF GYATDETPEL MPLSHVLATK
151 LGARLTEVRK NGTCPWLRPD GKTQVTVEYR NEGGAMVPIR VHTVLISTQH
201 DETVTNDEIA ADLKEHVIKP VIPEQYLDEK TIFHLNPSGR FVIGGPHGDA
251 GLTGRKIIID TYGGWGAHGG GAFSGKDPTK VDRSGAYVAR QAAKSIVAAG
301 LARRAIVQVS YAIGVPEPLS VFVDTYGTGA IPDKEILKIV KENFDFRPGM
351 IIINLDLKKG GNGRYLKTAA YGHFGRDDPD FTWEVVKPLK AEKPSSA
```

Spot No.: **41**

Uniprot Protein Accession: **Q6LBU9|Q6LBU9_MAIZE**

Plant species: **Zea mays**

Protein name: **Glyceraldehyde-3-phosphate dehydrogenase (Fragment)**

Peptide sequences: **R.LLDASHR.D; K.TLAEEVNQAFR.D; K.GTMTTTHSYTGDQR.L; K.VISWYDNEWGYSQR.V; R.DAAANELTGILEVCDVPLVSVDFR.C**

PFF Mascot score: **[481]** Sequence coverage %: **[18]**

Matched peptides No.: **[5]** p value: **7e-044**

Calculated Mr:**41265** Calculated pI: **7.21**

PFF Searched Score:

Protein score is -10*Log(P), where P is the probability that the observed match is a random event.
Protein scores greater than 62 are significant (p<0.05).
Protein scores are derived from ions scores as a non-probabilistic basis for ranking protein hits.

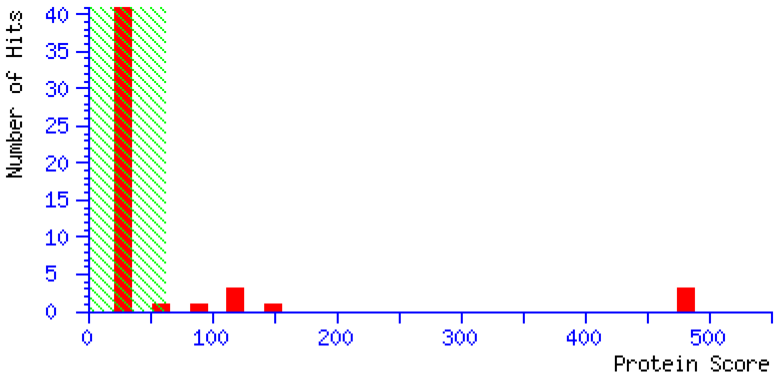

Matched peptide sequences shown in Bold Red

```
  1 EFSGLRSSAS LPMRRNATSD DFMSAVSFRT HAVGTSGGPR RAPTEAKLKV
 51 AINGFGRIGR NFLRCWHGRG DASPLDVIAI NDTGGVKQAS HLLKYDSTLG
101 IFDADVKPVG DNAISVDGKV IKVVSDRNPS NLPWGELGID LVIEGTGVFV
151 DREGAGKHIQ AGAKKVLITA PGKGDIPTYV VGVNADQYNP DEPIISNASC
201 TTNCLAPFVK VLDQKFGIIK GTMTTTHSYT GDQRLLDASH RDLRRARAAA
251 LNIVPTSTGA AKAVSLVLPN LKGKLNGIAL RVPTPNVSVV DLVVQVSKKT
301 LAEEVNQAFR DAAANELTGI LEVCDVPLVS VDFRCSDVSS TIDASLTMVM
351 GDDMVKVISW YDNEWGYSQR VVDLADICAN QWK
```

Spot No.: **42**

Uniprot Protein Accession: **B8A2L1|B8A2L1_MAIZE**

Plant species: **Zea mays**

Protein name: **Histidinol dehydrogenase, chloroplastic**

Peptide sequences: **K.YITVQSLTEEGLR.K; R.IDFSSIFGTVNPIVEDVR.V;**

PFF Mascot score: **[102]** Sequence coverage %: **[7]**

Matched peptides No.: **[2]** p value: **5.5e-006**

Calculated Mr:**47192** Calculated pI: **5.41**

PFF Searched Score:

Protein score is -10*Log(P), where P is the probability that the observed match is a random event.
Protein scores greater than 62 are significant (p<0.05).
Protein scores are derived from ions scores as a non-probabilistic basis for ranking protein hits.

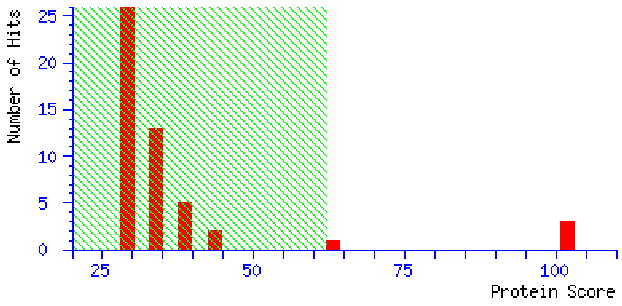

Matched peptide sequences shown in Bold Red

```
  1 MKSYRLSELS DTEVSGLKAR PRIDFSSIFG TVNPIVEDVR VRGDAAVKHY
 51 TEKFDKVMLD DVVVCVSDLP DAELDPAVKE AFDVAYDNIY AFHVSQKIPE
101 KTVENMKGVR CKRITRCIGS VGLYVPGGTA VLPSTALMLA VPAQIAGCKT
151 IVLATPPSRD GSICKEVLYC AKKAGVTHIL KAGGAQAISA MAWGTASCPK
201 AEKIFGPGNQ YVTAAKMILQ NSEAMVSIDM PAGPSEVLVI ADKYANPVHV
251 AADLLSQAEH GPDSQVVLVV AGDGVDLGAI EAEVSKQCNA LPRGEFASKA
301 LSHSFTVFAK DMVEAISFSN LYAPEHLIIN VKDAEQWEEF IENAGSVFLG
351 QWTPESVGDY ASGTNHVLPT YGYARMYSGV SLNSFLKYIT VQSLTEEGLR
401 KLGPYVAKMA EVEGLEAHKR AVTLRLQEVE ANVTV
```

Spot No.: **43**

Uniprot Protein Accession: **P00874|RBL_MAIZE**

Plant species: **Zea mays**

Protein name: **Ribulose bisphosphate carboxylase large chain**

Peptide sequences: **K.NHGMHFR.V; R.ACYECLR.G; R.DNGLLLHIHR.A; R.DNGLLLHIHR.A; K.TFQGPPHGIQVER.D**

PFF Mascot score: **[215]** Sequence coverage %: **[10]**

Matched peptides No.: **[5]** p value: **2.8e-017**

Calculated Mr:**53295** Calculated pI: **6.33**

PFF Searched Score:

Protein score is -10*Log(P), where P is the probability that the observed match is a random event.
Protein scores greater than 62 are significant (p<0.05).
Protein scores are derived from ions scores as a non-probabilistic basis for ranking protein hits.

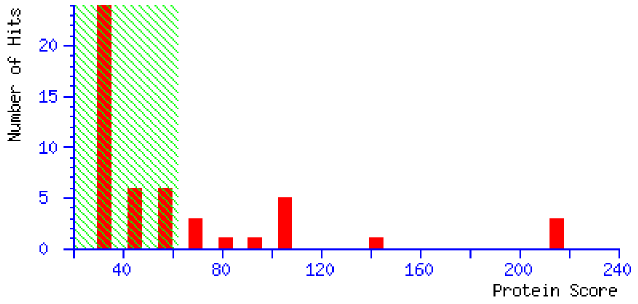

Matched peptide sequences shown in Bold Red

```
  1 MSPQTETKAS VGFKAGVKDY KLTYYTPEYE TKDTDILAAF RVTPQLGVPP
 51 EEAGAAVAAE SSTGTWTTVW TDGLTSLDRY KGRCYHIEPV PGDPDQYICY
101 VAYPLDLFEE GSVTNMFTSI VGNVFGFKAL RALRLEDLRI PPAYSKTFQG
151 PPHGIQVERD KLNKYGRPLL GCTIKPKLGL SAKNYGRACY ECLRGGLDFT
201 KDDENVNSQP FMRWRDRFVF CAEAIYKAQA ETGEIKGHYL NATAGTCEEM
251 IKRAVFAREL GVPIVMHDYL TGGFTANTTL SHYCRDNGLL LHIHRAMHAV
301 IDRQKNHGMH FRVLAKALRM SGGDHIHSGT VVGKLEGERE ITLGFVDLLR
351 DDFIEKDRSR GIFFTQDWVS MPGVIPVASG GIHVWHMPAL TEIFGDDSVL
401 QFGGGTLGHP WGNAPGAAAN RVALEACVQA RNEGRDLARE GNEIIKAACK
451 WSAELAAACE IWKEIKFDGF KAMDTI
```

Spot No.: **44**

Uniprot Protein Accession: **K7V7B1|K7V7B1_MAIZE**

Plant species: **Zea mays**

Protein name： **Transketolase isoform 1**

Peptide sequences: **K.NPYWFNR.D; K.YTPESPGDATR.N; R.ISIEAGSTLGWQK.Y; R.FEALGWHTIWVK.N; R.HTPEGAALEADWNAK.F; K.LIAFYDDNHISIDGDTEIAFTEDVSTR.F**

PFF Mascot score: **[519]** Sequence coverage %: **[13]**

Matched peptides No.: **[6]** p value: **1.1e-047**

Calculated Mr:**69063** Calculated pI: **5.46**

PFF Searched Score:

Protein score is -10*Log(P), where P is the probability that the observed match is a random event.
Protein scores greater than 62 are significant (p<0.05).
Protein scores are derived from ions scores as a non-probabilistic basis for ranking protein hits.

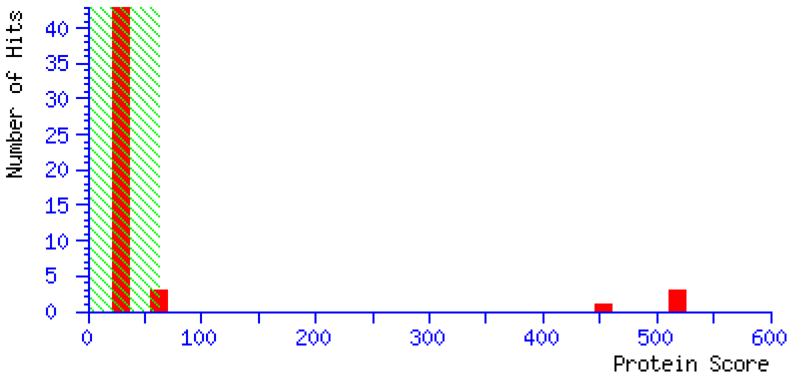

Matched peptide sequences shown in Bold Red

```
  1 MGCAPMGHVL YDEVMRYNPK NPYWFNRDRF VLSAGHGCML QYALLHLAGY
 51 DSVKEEDLKQ FRQWGSRTPG HPENFETPGV EVTTGPLGQG IANAVGLALA
101 EKHLAARFNK PDSEIVDHYT YVILGDGCQM EGIANEACSL AGHWGLGKLI
151 AFYDDNHISI DGDTEIAFTE DVSTRFEALG WHTIWVKNGN TGYDDIRAAI
201 KEAKAVTDKP TLIKVTTTIG FGSPNKANSY SVHGSALGAK EVEATRQNLG
251 WPYDTFFVPE DVKSHWSRHT PEGAALEADW NAKFAEYEKK YADDAATLKS
301 IITGELPTGW VDALPKYTPE SPGDATRNLS QQCLNALANV VPGLIGGSAD
351 LASSNMTLLK MFGDFQKDTA EERNVRFGVR EHGMGAICNG IALHSPGFVP
401 YCATFFVFTD YMRGAMRISA LSEAGVIYVM THDSIGLGED GPTHQPIEHL
451 VSFRAMPNIL MLRPADGNET AGAYKVAVLN RKRPSILALS RQKLPHLPGT
501 SIEGVEKGGY TISDNSTGNK PDIIVMGTGS ELEIAAKAAD ELRKEGKTVR
551 VVSFVSWELF DEQSDEYKES VLPAAVTARI SIEAGSTLGW QKYVGAQGKA
601 IGIDKFGASA PAGTIYKEYG ITVESIIAAA KSF
```

Spot No.: **45**

Uniprot Protein Accession: **A0A096S2Q4|A0A096S2Q4_MAIZE**

Plant species: **Zea mays**

Protein name: **Uncharacterized protein**

Peptide sequences: **R.AQGGPGAGPGGLGGPMDFGR.S; K.APCIVFIDEIDAVGR.Q; R.GQAGGLTFFAPSEER.L; R.SYLENQMAVALGGR.V; R.VAEEVIFGQDNVTTGASNDFMQVSR.V**

PFF Mascot score: **[104]** Sequence coverage %: **[12]**

Matched peptides No.: **[5]** p value: **3.5e-006**

Calculated Mr: **72889** Calculated *p*I: **5.62**

PFF Searched Score:

Protein score is -10*Log(P), where P is the probability that the observed match is a random event.
Protein scores greater than 62 are significant (p<0.05).
Protein scores are derived from ions scores as a non-probabilistic basis for ranking protein hits.

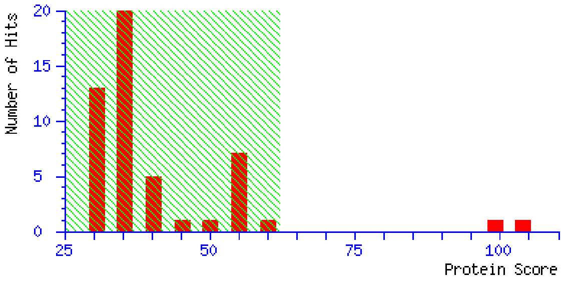

Matched peptide sequences shown in Bold Red

1 MAPPSVSASH LLITASLPKP KPSSLRPPRL PLAKPLPASA ALLALAASPA
51 LAADAPAPAP APTPAPAPEL QAEAPTPTVN PFSSSLLTAP KPSAAASDLP
101 EGAQWRYSEF LSAVKRGKVE RVRFSKDGGL LQLTAVDGRR ATVVVPNDPD
151 LIDILATNGV DISVSEGESA GPGGFVAFVG NLLFPFIAFA GLFFLFRR**AQ**
201 **GGPGAGPGGL** **GGPMDFGR**SK SKFQEVPETG VTFLDVAGAD QAKLELQEVV
251 DFLKNPDKYT ALGAKIPKGC LLVGPPGTGK TLLARAVAGE AGVPFFSCAA
301 SEFVELFVGV GASRVRDLFE KAKAK**APCIV** **FIDEIDAVGR** QRGAGLGGGN
351 DEREQTINQL LTEMDGFAGN SGVIVLAATN RPDVLDSALL RPGRFDRQVT
401 VDRPDVAGRV KILEVHSRGK ALAKDVDFDK IARRTPGFTG ADLQNLMNEA
451 AILAARRDLK EISKDEISDA LERIIAGPEK KNAVVSEEKK RLVAYHEAGH
501 ALVGALMPEY DPVAKISIIP R**GQAGGLTFF** **APSEER**LESG LYSR**SYLENQ**
551 **MAVALGGRVA** **EEVIFGQDNV** **TTGASNDFMQ** **VSR**VARQMVE RFGFSKKIGQ
601 VAIGGPGGNP FLGQQMSSQK DYSMATADVV DAEVRELVEK AYSRARQIIT
651 THIDILHKLA QLLIEKETVD GEEFMSLFID GQAELFVA

Spot No.: **46**

Uniprot Protein Accession: **P00874|RBL_MAIZE**

Plant species: **Zea mays**

Protein name: **Ribulose bisphosphate carboxylase large chain**

Peptide sequences: **K.NHGMHFR.V; R.ACYECLR.G; K.DTDILAAFR.V; R.VALEACVQAR.N; R.DNGLLLHIHR.A; R.FVFCAEAIYK.A; R.EITLGFVDLLR.D; K.TFQGPPHGIQVER.D K.WSAELAAACEIWK.E**

PFF Mascot score: **[701]**   Sequence coverage %: **[18]**

Matched peptides No.: **[9]**   p value: **7e-066**

Calculated Mr:**53295**   Calculated pI: **6.33**

PFF Searched Score:

Protein score is -10*Log(P), where P is the probability that the observed match is a random event.
Protein scores greater than 62 are significant (p<0.05).
Protein scores are derived from ions scores as a non-probabilistic basis for ranking protein hits.

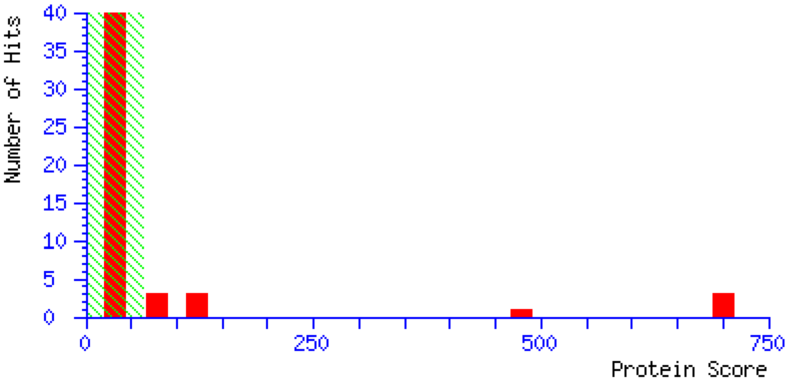

Matched peptide sequences shown in Bold Red

```
  1 MSPQTETKAS VGFKAGVKDY KLTYYTPEYE TKDTDILAAF RVTPQLGVPP
 51 EEAGAAVAAE SSTGTWTTVW TDGLTSLDRY KGRCYHIEPV PGDPDQYICY
101 VAYPLDLFEE GSVTNMFTSI VGNVFGFKAL RALRLEDLRI PPAYSKTFQG
151 PPHGIQVERD KLNKYGRPLL GCTIKPKLGL SAKNYGRACY ECLRGGLDFT
201 KDDENVNSQP FMRWRDRFVF CAEAIYKAQA ETGEIKGHYL NATAGTCEEM
251 IKRAVFAREL GVPIVMHDYL TGGFTANTTL SHYCRDNGLL LHIHRAMHAV
301 IDRQKNHGMH FRVLAKALRM SGGDHIHSGT VVGKLEGERE ITLGFVDLLR
351 DDFIEKDRSR GIFFTQDWVS MPGVIPVASG GIHVWHMPAL TEIFGDDSVL
401 QFGGGTLGHP WGNAPGAAAN RVALEACVQA RNEGRDLARE GNEIIKAACK
451 WSAELAAACE IWKEIKFDGF KAMDTI
```

Spot No.: **47**

Uniprot Protein Accession: **B4FLE1|B4FLE1_MAIZE**

Plant species: **Zea mays**

Protein name: **Uncharacterized protein**

Peptide sequences**: K.EEYPYFPGQEPTLR.G; K.GVGPYIQEIEELCR.D; K.AFSSFLSLSR.F**

PFF Mascot score: **[131]** Sequence coverage %: **[12]**

Matched peptides No.: **[3]** p value: **7e-009**

Calculated Mr: **33049** Calculated *p*I: **5.64**

PFF Searched Score:

Protein score is -10*Log(P), where P is the probability that the observed match is a random event.
Protein scores greater than 62 are significant (p<0.05).
Protein scores are derived from ions scores as a non-probabilistic basis for ranking protein hits.

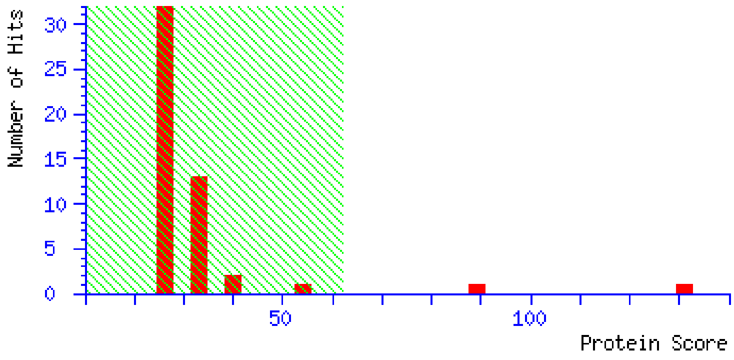

Matched peptide sequences shown in Bold Red

1 MAASSSCKVI DSHLHVWATP QQAK**EEYPYF PGQEPTLR**GD ADFLLECMSE
51 AGVDGAVIVQ PINHMFDHSL VTSVLKKYPS KFVGCCLANP ADDGTGIKQL
101 EHLIVQEKYR AVRFNPNLWP SGQKMTNEVG RSLFSKAGEL GVPLGIMTMK
151 **GVGPYIQEIE ELCR**DYPATT VILDHMAFCK PPTNDEEEK**A FSSFLSLSR**F
201 PQVYVKYSAL FRITREAYPY EDTAQLLSSV ISHYGASRVM WGSDFPYVVP
251 ECGYKGGREA ISHVASKIPV SQSDLEWILG KTVSQLFQGA WVTP

Spot No.: **48**

Uniprot Protein Accession: **A0A0B4J3F5|A0A0B4J3F5_MAIZE**

Plant species: **Zea mays**

Protein name: **Uncharacterized protein**

Peptide sequences: **R.YGEFAQATYDSFDYDR.F; K.TFFHDVGLGGIGYEVTR.Y; R.LEWVADLTANQIPLR.E; R.EQVLAEVR.K; R.VAPVCVFSFAGPR.V; R.GSGAGFEPR.G ; R.VLDDHPEDTDHHLQR.L**

PFF Mascot score: **[192]** Sequence coverage %: **[18]**

Matched peptides No.: **[7]** p value: **5.5e-015**

Calculated Mr: **56532** Calculated *p*I: **6.29**

PFF Searched Score:

Protein score is -10*Log(P), where P is the probability that the observed match is a random event.
Protein scores greater than 62 are significant (p<0.05).
Protein scores are derived from ions scores as a non-probabilistic basis for ranking protein hits.

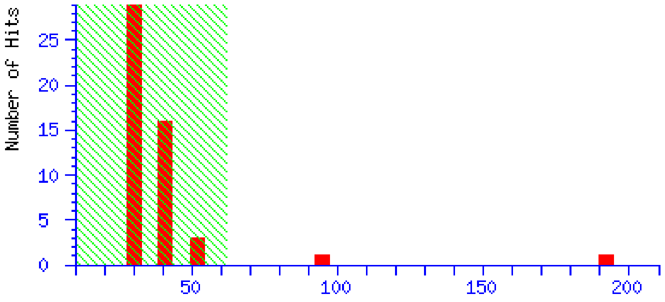

Matched peptide sequences shown in Bold Red

1 MAATVSSCLS LVPTVHHRAG GFPAVALTNT LQRRRRSRRY LVVVASATRA

51 EQEAAPVTIE DVDLSSHQAA PDDGELAARW PEIHGSNNWE GLLDPIDGVL

101 LQELIR**YGEF AQATYDSFDY DR**FSPYCGSC KYPAK**TFFHD VGLGGIGYEV**

151 **TR**YLYATCND LKFPNFGIKT AANAKMWSES GTFIGYVAVS TDEETARLGR

201 RDIAVAWRGT ITR**LEWVADL TANQIPLR**ET GVPCPDPDVK VERGFVALYT

251 DKGTGCRFCR YSAR**EQVLAE VR**KLVDLYHG RGEQVSVTVT GHSLGSALAM

301 LCAFDIAETR ANVSPGDR**VA PVCVFSFAGP R**VGNVAFRRR FERELGVRAL

351 RVVNVHDSVP KVPGVFFNES AFPELVLRAA DRLGLGGVYT HLGVLLQLDH

401 KVSPFLKETL DLSCYHNLEA HLHLLDGFR**G SGAGFEPR**GR DPALVNKSTD

451 FLREDHMVPP VWYQAENKGM VRTEDGRWVL PPRQR**VLDDH PEDTDHHLQR**

501 LGLTA

Spot No.:**49**

Uniprot Protein Accession: **B6TEW2|B6TEW2_MAIZE**

Plant species: **Zea mays**

Protein name： **Ferredoxin--NADP reductase, leaf isozyme**

Peptide sequences: **K.DPNATIIMLATGTGIAPFR.S**

PFF Mascot score: **[133]** Sequence coverage %: **[5]**

Matched peptides No.: **[1]** p value: **4.4e-009**

Calculated Mr:**37878** Calculated pI: **8.37**

PFF Searched Score:

Protein score is -10*Log(P), where P is the probability that the observed match is a random event.
Protein scores greater than 62 are significant (p<0.05).
Protein scores are derived from ions scores as a non-probabilistic basis for ranking protein hits.

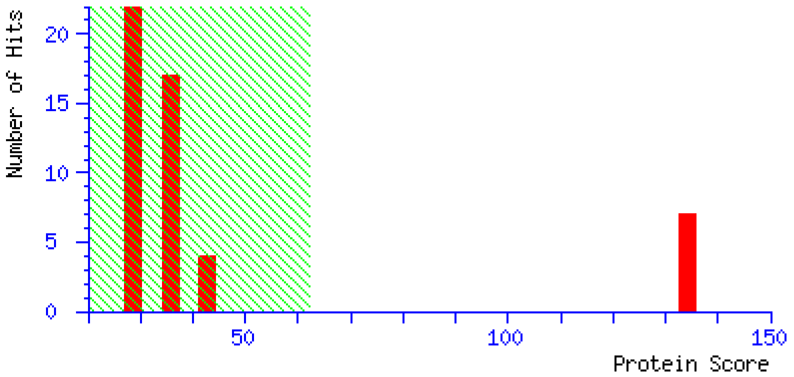

Matched peptide sequences shown in Bold Red

```
  1 MRPPPVPAAA RRPARGAAAR AGVHHRDRGG GAGXKKLEKV SKKQEEGLVT
 51 NKYKPKEPYV GRCLLNTRIT GDQAPGETWH MVFSTEGEVP YREGQSIGVI
101 ADGEDKNGKP HKLRLYSIAS SALGDFGDSK TVSLCVKRLV YTNDQGEVVK
151 GVCSNFLCDL KPGAEVKITG PVGKEMLMPK DPNATIIMLA TGTGIAPFRS
201 FLWKMFFEEH EDYKYTGLAW LFLGVPTSDT LLYKEELEKM KEMAPDNFRL
251 DFAVSREQTN AAGEKMYIQT RMAEYKEELW ELLKKDNTYV YMCGLKGMEK
301 GIDDIMLDLA AKDGINWLDY KKQLKKSEQW NVEVY
```

Spot No.: **50**

Uniprot Protein Accession: **B4FQW0|B4FQW0_MAIZE**

Plant species: **Zea mays**

Protein name: **Stem-specific protein TSJT1**

Peptide sequences: **K.DEVFCLFEGVLDNLGR.L**

PFF Mascot score: **[131]** Sequence coverage %: **[6]**

Matched peptides No.: **[1]** p value: **5.5e-009**

Calculated Mr:**25050** Calculated pI: **5.23**

PFF Searched Score:

Protein score is -10*Log(P), where P is the probability that the observed match is a random event.
Protein scores greater than 62 are significant (p<0.05).
Protein scores are derived from ions scores as a non-probabilistic basis for ranking protein hits.

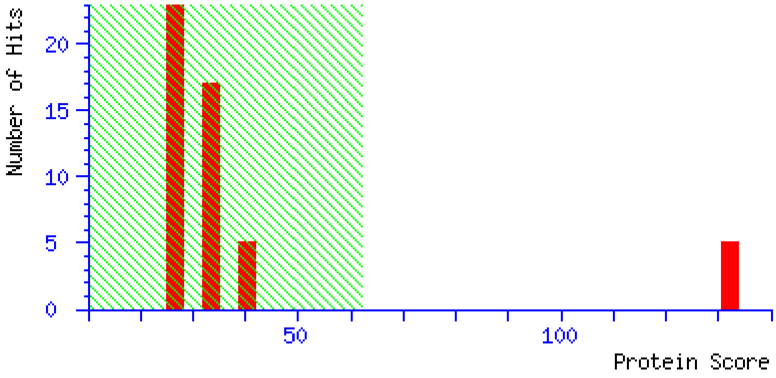

Matched peptide sequences shown in Bold Red

```
  1 MLAVFSGEVV EVPAELVAAG SRTPSPKTKA SELVARFLGT SCPAAVSVRL
 51 ADLGHLAYSH ANQALLRPRS FAAKDEVFCL FEGVLDNLGR LSQQYGLSKG
101 ANEVLLVIEA YKALRDRAPY PASLMLAQLA GAYAFVLFDA STNSLLVASG
151 GDVPLFWGVT ADGCVAFSDD IDVLKGSCGK SLAPFPQGCF YSNALGGLKC
201 YENPKNKVTA VPANEEEICG ATFQVEGATV LTALH
```

Spot No.: **51**

Uniprot Protein Accession: **A0A096QKN7|A0A096QKN7_MAIZE**

Plant species: **Zea mays**

Protein name: **Uncharacterized protein**

Peptide sequences: **R.EFPGQVLR.Y; K.QYYAVSVLTR.T; K.TIADYGSPEEFLSQVDYLLGR.Q**

PFF Mascot score: **[309]** Sequence coverage %: **[12]**

Matched peptides No.: **[3]** p value: **1.1e-026**

Calculated Mr:**32073** Calculated pI: **9.5**

PFF Searched Score:

Protein score is -10*Log(P), where P is the probability that the observed match is a random event.
Protein scores greater than 62 are significant ($p<0.05$).
Protein scores are derived from ions scores as a non-probabilistic basis for ranking protein hits.

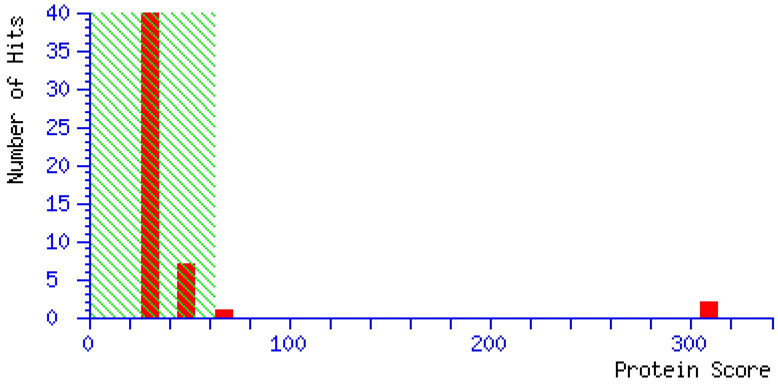

Matched peptide sequences shown in Bold Red

```
  1 MRPHGRLPSL TRRGLANAPA LARVVAPTHT LLPHESGPRR SQAMASTSCC
 51 LLHQSTARLG ASPRLAAAPP RSAQLLVCKA RKQEAAADQS DVSRRAALAL
101 LAGVTAAAGA KVGPAAAAYG EAANVFGKPK ASTEYIAYSG DGFKLLIPSK
151 WNPSKEREFP GQVLRYEDNF DANSNVSVII QPTSKKTIAD YGSPEEFLSQ
201 VDYLLGRQAY GGKTDSEGGF ETGAVATANV LESSTPVIDG KQYYAVSVLT
251 RTADGDEGGK HQLIAATVSD GKLYICKAQA GDKRWFKGAR KGVEKAASSF
301 SVA
```

Spot No.: **52**

Uniprot Protein Accession: **C0PD30|C0PD30_MAIZE**

Plant species: **Zea mays**

Protein name： **Fructose-bisphosphate aldolase**

Peptide sequences: **R.LASIGLENTEANR.Q; R.TLLVTAPGLGQYISGAILFEETLYQSAVDGR.K; K.GLVPLAGSNNESWCQGLDGLASR.E; R.EAAYYQQGAR.F; R.YAAISQDNGLVPIVEPEILLDGEHGIER.T; K.AAQDALLLR.A**

PFF Mascot score: **[322]** Sequence coverage %: **[32]**

Matched peptides No.: **[6]** p value: **5.5e-028**

Calculated Mr: **38408** Calculated *p*I: **6.37**

PFF Searched Score:

Protein score is -10*Log(P), where P is the probability that the observed match is a random event.
Protein scores greater than 62 are significant (p<0.05).
Protein scores are derived from ions scores as a non-probabilistic basis for ranking protein hits.

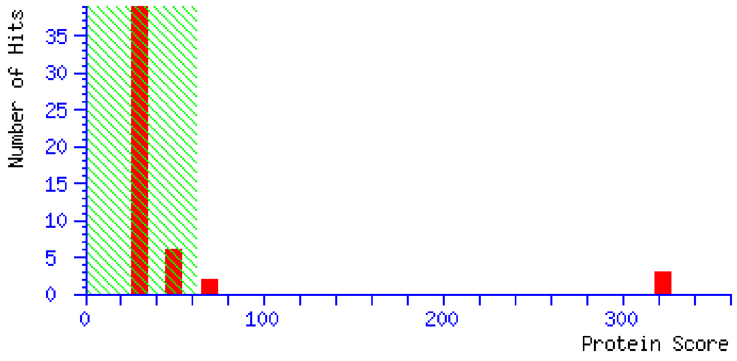

Matched peptide sequences shown in Bold Red

1 MLCIRTYVRM CGQKTIASPG RGILAMDESN ATCGKR**LASI** **GLENTEANR**Q
51 AYR**TLLVTAP** **GLGQYISGAI** **LFEETLYQSA** **VDGR**KIVDIL AEQGIVPGIK
101 VDK**GLVPLAG** **SNNESWCQGL** **DGLASREAAY** **YQQGAR**FAKW RTVVSIPNGP
151 SELAVKEAAW GLAR**YAAISQ** **DNGLVPIVEP** **EILLDGEHGI** **ER**TFEVAQKV
201 WAETFYAMAE NNVMFEGILL KPSMVTPGAE AKDRATPEQV AAYTLKLLHR
251 RIPPSVPGIM FLSGGQSEVE ATQNLNAMNQ GPNPWHVSFS YARALQNTCL
301 KTWGGQPDKV K**AAQDALLLR** AKANSLAQLG KYTSDGEAAE AKEGMFVKNY
351 SY

Spot No.: **53**

Uniprot Protein Accession: **Q6TM44|Q6TM44_MAIZE**

Plant species: **Zea mays**

Protein name: **Germin-like protein**

Peptide sequences: **K.SSVTANDFYFHGLAGQGK.I**

PFF Mascot score: **[66]** Sequence coverage %: **[8]**

Matched peptides No.: **[1]** p value: **0.02**

Calculated Mr:**22101** Calculated pI: **6.02**

PFF Searched Score:

Protein score is -10*Log(P), where P is the probability that the observed match is a random event.
Protein scores greater than 62 are significant (p<0.05).
Protein scores are derived from ions scores as a non-probabilistic basis for ranking protein hits.

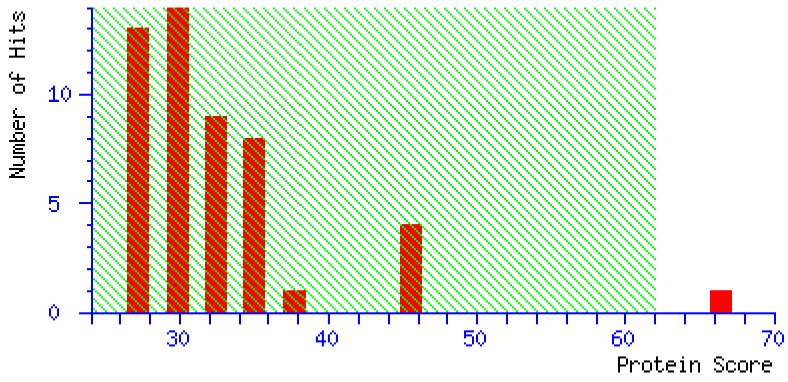

Matched peptide sequences shown in Bold Red

```
  1 MAKMVLLCVL VSFLLMPLAS LALTQDFCVA DLTCSDTPAG YPCKSSVTAN
 51 DFYFHGLAGQ GKINPLIKAA VTPAFVGQFP GVNGLGISAA RLDIEVGGVV
101 PLHTHPAGSE LLFVTQGTVA AGFISSGSNT VYTKTLYAGD IMVFPQGLLH
151 YQYNAGTGAA VGLVAFSSPN PGLQITDFAL FANNLPSAVV EKVTFLDDAQ
201 VKKLKSVLGG SG
```

Spot No.: 54

Uniprot Protein Accession: **B4FRZ2|B4FRZ2_MAIZE**

Plant species: **Zea mays**

Protein name: **Pyridoxin biosynthesis protein ER1**

Peptide sequences: **R.GGVIMDVVTPEQAR.L ; R.LAEEAGACAVMALER.V ; R.VPFVCGCR.D**

PFF Mascot score: **[110]** Sequence coverage %: **[11]**

Matched peptides No.: **[3]** p value: **8.8e-007**

Calculated Mr: **33832** Calculated *p*I: **6.12**

PFF Searched Score:

Protein score is -10*Log(P), where P is the probability that the observed match is a random event.
Protein scores greater than 62 are significant (p<0.05).
Protein scores are derived from ions scores as a non-probabilistic basis for ranking protein hits.

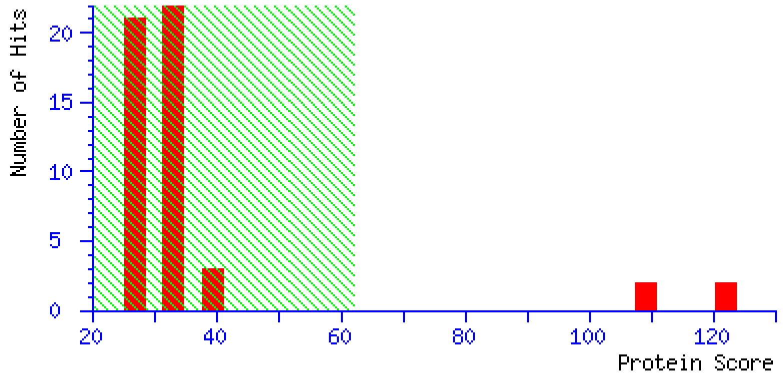

Matched peptide sequences shown in Bold Red

```
  1 MASDGSGVVT VVGSNGAELL EPSKQPKSAT FSVKVGLAQM LRGGVIMDVV
 51 TPEQARLAEE AGACAVMALE RVPADIRAQG GVARMSDPGL IRDIKRAVTI
101 PVMAKARIGH FVEAQILEAV GVDYVDESEV LTPADDAHHI NKHNFRVPFV
151 CGCRDLGEAL RRVREGAAMI RTKGEAGTGN VVEAVRHVRS VMGDVRALRS
201 MDDDEVFAYA KRIAAPYDLV MQTKQLGRLP VVQFAAGGVA TPADAALMMQ
251 LGCDGVFVGS GIFKSGDPAR RARAIVQAVT HYSDPTILAD VSTGLGEAMV
301 GINLNDPKVE RYAARSE
```

Spot No.: **55**

Uniprot Protein Accession: **B4FDW3|B4FDW3_MAIZE**

Plant species: **Zea mays**

Protein name: **Uncharacterized protein**

Peptide sequences: **K.ENGGMLGWVR.R;K.LQDPSFLEEAQLIDVR.E**

PFF Mascot score: **[62]** Sequence coverage %: **[8]**

Matched peptides No.: **[2]** p value: **0.057**

Calculated Mr:**33786** Calculated pI: **7.67**

PFF Searched Score:

Protein score is -10*Log(P), where P is the probability that the observed match is a random event.
Protein scores greater than 62 are significant (p<0.05).
Protein scores are derived from ions scores as a non-probabilistic basis for ranking protein hits.

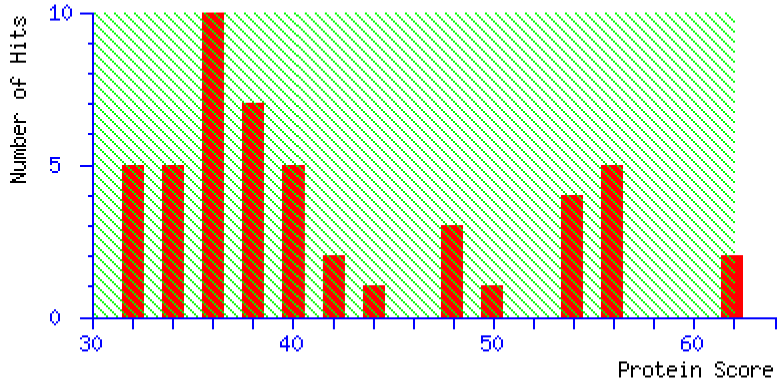

Matched peptide sequences shown in Bold Red

```
  1 MFGLRARAAA QLQRPTPSPS SASSASFSLA RLGCASPLAA LAPLASAPPS
 51 PSARETQPTS LSARWPAAPM RPWGTRGHPR PTTRVLCTAA GSVQREGKEL
101 LVQHLLVGEK DVRLLVDLEK NIIAEGADLS DLAVEHSLCP SKENGGMLGW
151 VRRGQMVPEF EEAAFSAPLN KVVRCKTKFG WHLVQVLAER DQCVLQDIDP
201 EELHAKLQDP SFLEEAQLID VREPDEVEKA SLPGFKVLPL RQFGTWGPVM
251 TDEFNPQKDT YVLCHHGMRS MQVAKWLQSQ GFKKVYNVAG GIHAYAVKAD
301 SSIPTY
```

Spot No.: **56**

Uniprot Protein Accession: **K7V496|K7V496_MAIZE**

Plant species: **Zea mays**

Protein name: **Sucrose-phosphatase1**

Peptide sequences: **K.IIHSNER.C; R.LFITQTGSDSWVGR.F**

PFF Mascot score: **[64]** Sequence coverage %: **[7]**

Matched peptides No.: **[2]** p value: **0.039**

Calculated Mr: **30032** Calculated *p*I: **7.04**

PFF Searched Score:

Protein score is -10*Log(P), where P is the probability that the observed match is a random event.
Protein scores greater than 62 are significant ($p<0.05$).
Protein scores are derived from ions scores as a non-probabilistic basis for ranking protein hits.

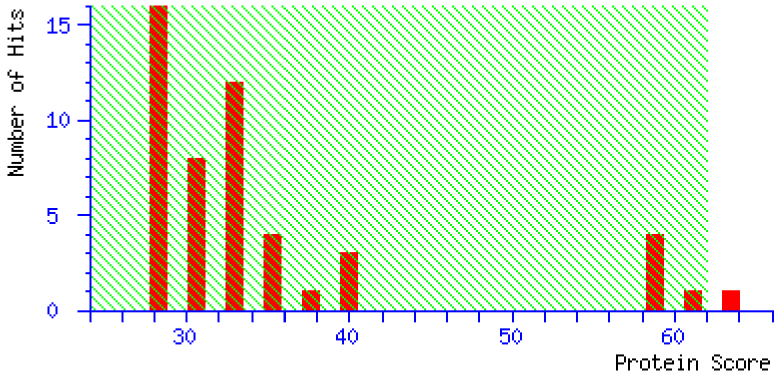

Matched peptide sequences shown in Bold Red

```
  1 MWDAKIIYSG GQDLDILPQG AGKGQALAYL LKKLSSCGKP PNNTLVCGDS
 51 GNDAELFSIP GVHGVMVSNA QEELLQWYTE NAKDNPKIIH SNERCAAGII
101 QAIGHFKLGP NISPRDLQFP YAKEASFKPT DAVVKFYVLY EKWRRAEVPK
151 SDSVIKYFKN ITHANGVTIH PAGLELSLHA SIDALGSCYG DKQGRKYRAW
201 VDRLFITQTG SDSWVGRFDL WESEGDVRAC SLSSLALILK AESPEGFVLT
251 HIQKTWLNGY SSGVEQAFKL
```

Spot No.: **57**

Uniprot Protein Accession: **C0PFV4|C0PFV4_MAIZE**

Plant species: **Zea mays**

Protein name: **Cytokinin inducible protease1**

Peptide sequences: **R.YELHHK.L; K.TAIAEGLAQR.I; R.VLELSLEEAR.Q; R.VVDEGYNPSYGAR.P; R.GELQCIGATTLDEYR.K; K.LIGSPPGYVGYTEGGQLTEAVR.R**

PFF Mascot score: **[160]** Sequence coverage %: **[8]**

Matched peptides No.: **[6]** p value: **8.8e-012**

Calculated Mr:**102149** Calculated *p*I: **6.24**

PFF Searched Score:

Protein score is -10*Log(P), where P is the probability that the observed match is a random event.
Protein scores greater than 62 are significant (p<0.05).
Protein scores are derived from ions scores as a non-probabilistic basis for ranking protein hits.

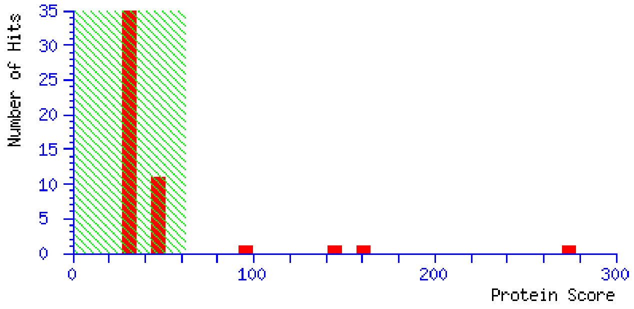

Matched peptide sequences shown in Bold Red

1 MAGTLLQSVA LGTPFGGRMR THCWRSHGTR RPASMLAVSL SRPVKMSAFV
51 GLRSVHSFSV TPTVSNSRSA VASYRSSRQT RRSRFVTQAM FERFTEKAIK
101 VIMLAQEEAR RLGHNFVGTE QILLGLIGEG TGIAAKVLKS MGINLKDARV
151 EVEKIIGRGN GFVAVEIPFT PRAKR**VLELS LEEAR**QLGHN YIGSEHLLLG
201 LLREGEGVAA RVLESLGADP SNIRTQVIRM IGETTEAVGA GVGGGSSGNK
251 MPTLEEYGTN LTKLAEEGKL DPVVGRQPQI ERVVQILGRR TKNNPCLIGE
301 PGVGK**TAIAE GLAQR**ISTGD VPETIEGKKV ITLDMGLLVA GTKYRGEFEE
351 RLKKLMEEIK QSDEIILFID EVHTLIGAGA AEGAIDAANI LKPALAR**GEL**
401 **QCIGATTLDE YR**KHIEKDPA LERRFQPVKV PEPTVDETIE ILRGLRER**YE**
451 **IHHK**LRYTDE ALIAAAKLSY QYISDRFLPD KAIDLIDEAG SRVRLQHAQV
501 PEEARELDKE LKQVTKQKNE AVRSQDFEKA GELRDREMEL KAQITALIDK
551 SKELSKAEEE SGETGPMVNE EDIQHIVSSW TGIPVEKVSS DESDKLLKME
601 ETLHKRVIGQ DEAVVAISRS IRRARVGLKN PNRPIASFIF AGPTGVGKSE
651 LAKALAAYYF GSEEAMIRLD MSEFMERHTV SK**LIGSPPGY VGYTEGGQLT**
701 **EAVR**RRPYTV VLFDEIEKAH PDVFNMMLQI LEDGRLTDSK GRTVDFKNTL
751 LIMTSNVGSS VIEKGGRKIG FDLDSDEKDS SYSRIKSLVI EEMKQYFRPE
801 FLNRLDEMIV FRQLTKLEVK EIADIMLQEV FDRLKAKDIN LQVTEKFKER
851 **VVDEGYNPSY GAR**PLRRAIM RLLEDSLAEK MLAGEVKEGD SAIVDVDSEG
901 KVVVLNGQGG IPELSTPAIT V

Supplementary Figure S3. Main pathways involved in transgenic maize.

Carbon fixation in photosynthetic organisms: 8 proteins

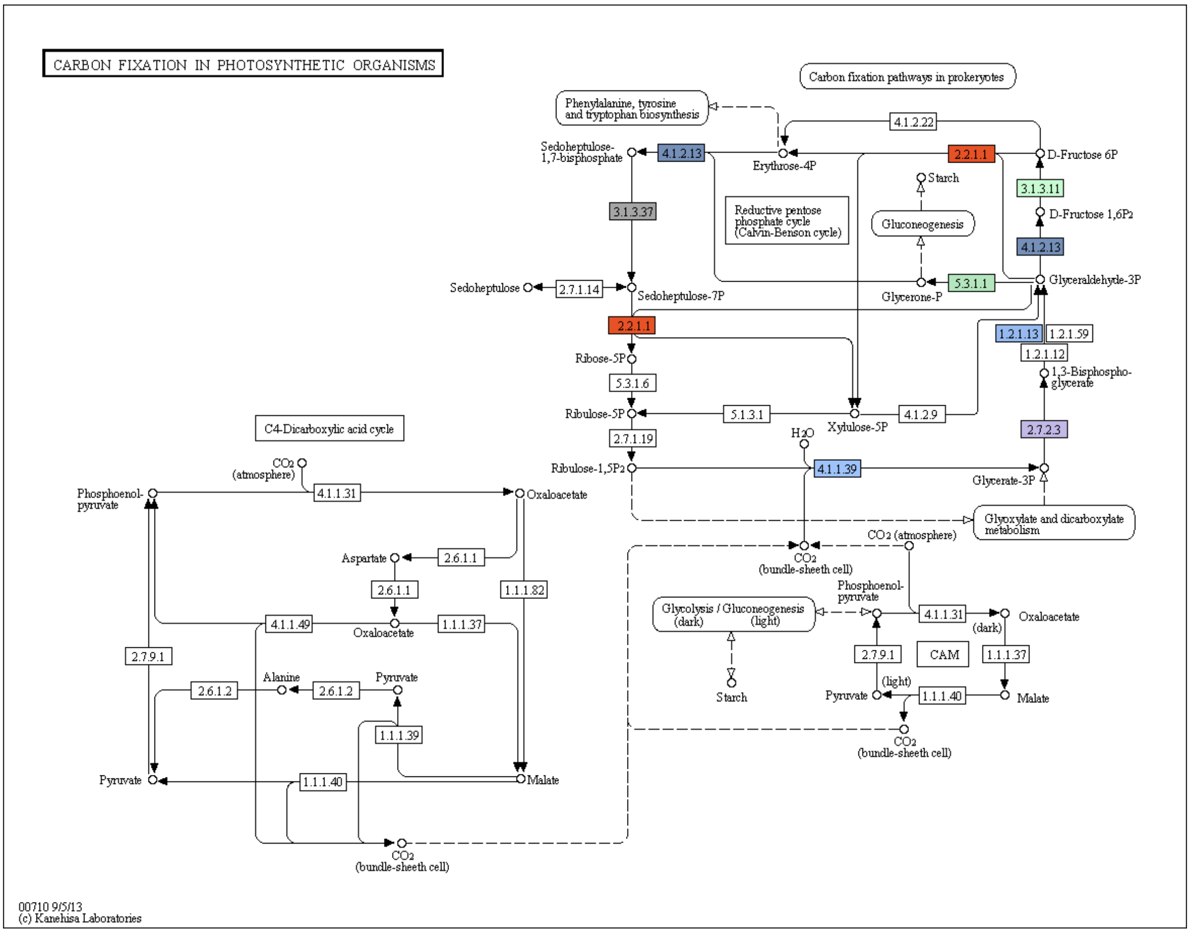

Glycolysis / Gluconeogenesis: 6 proteins

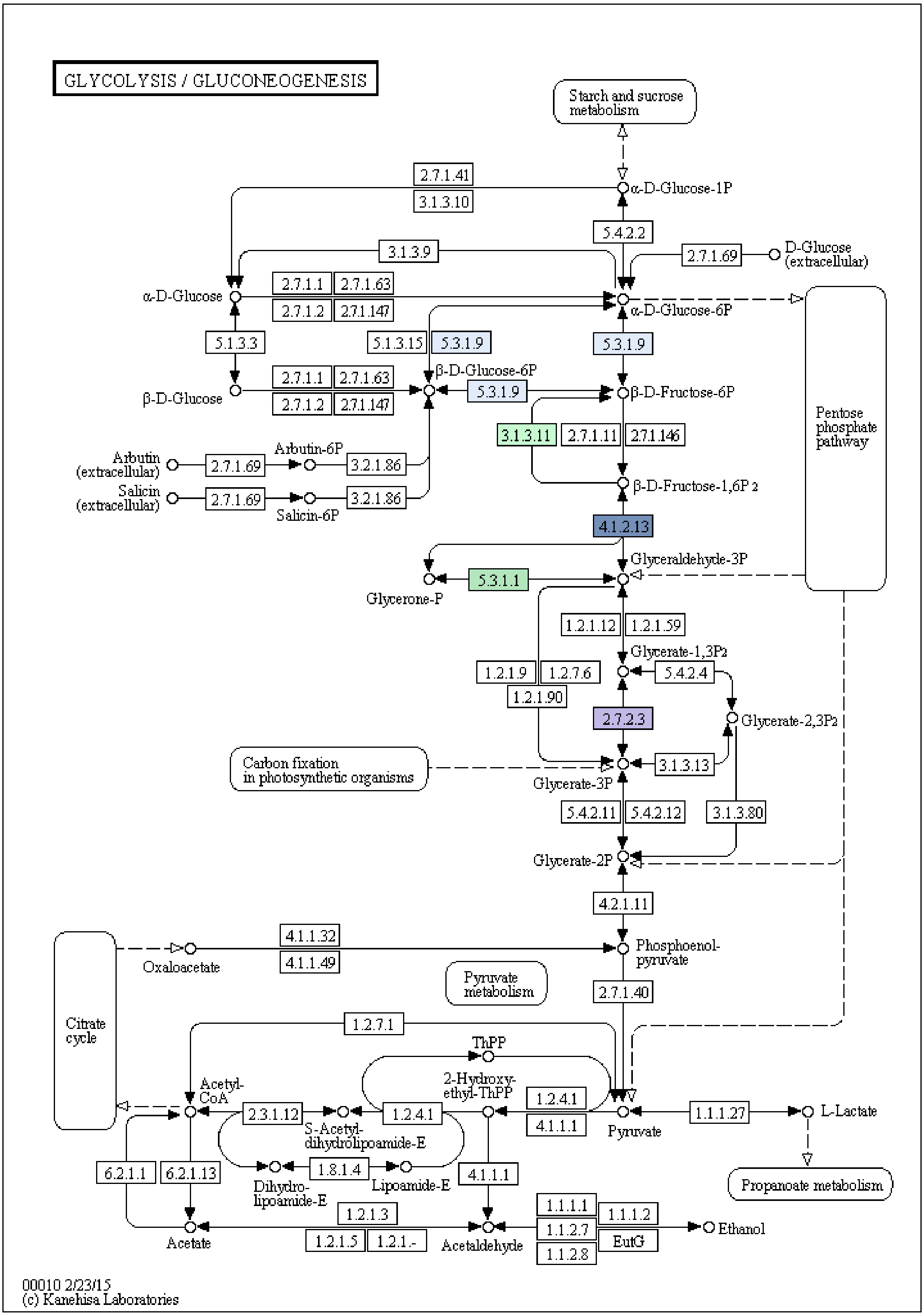

Pentose phosphate pathway: 4 proteins

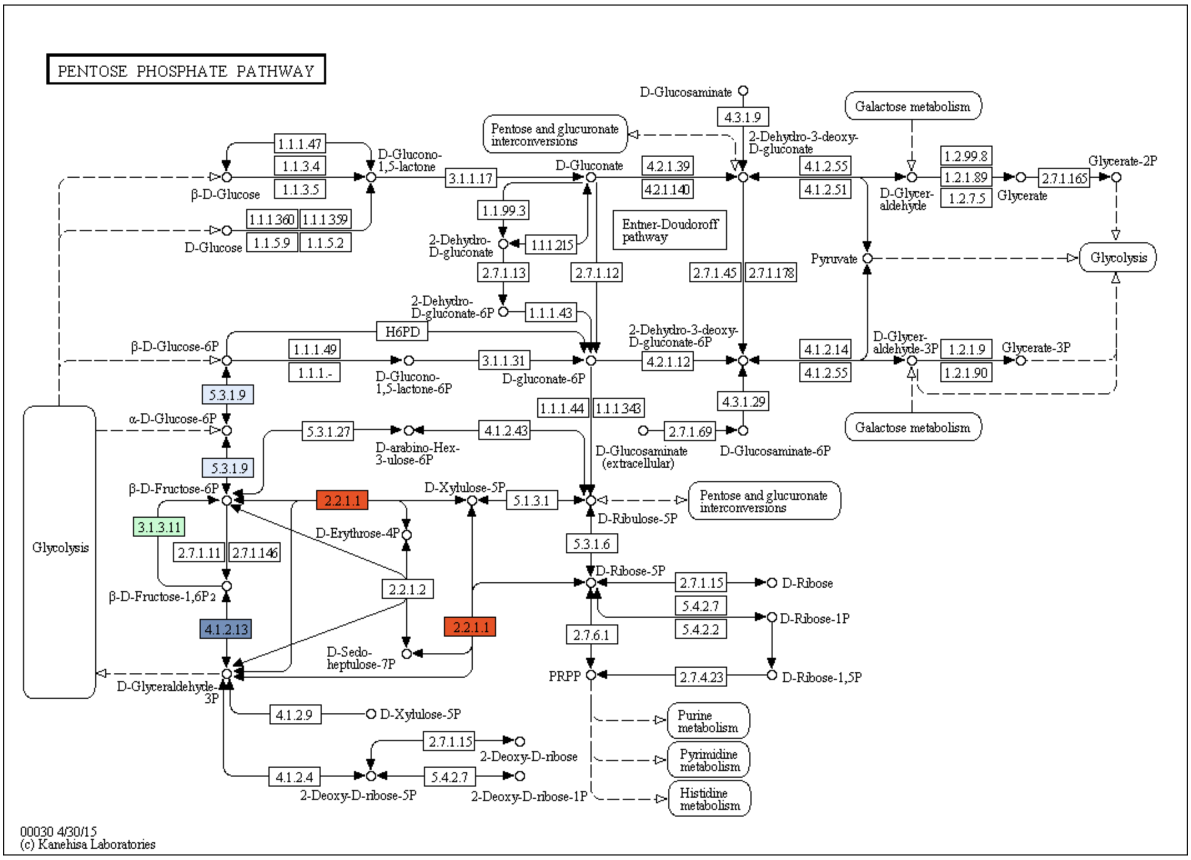

Purine metabolism: 4 proteins

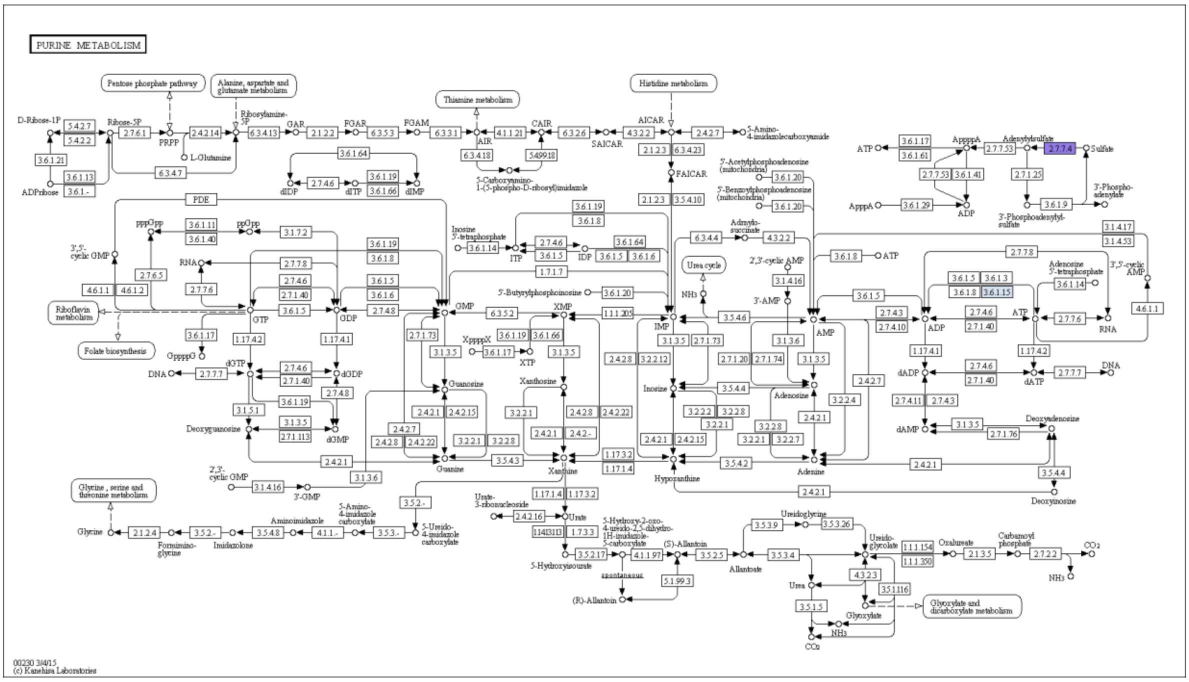

Fructose and mannose metabolism: 3 proteins

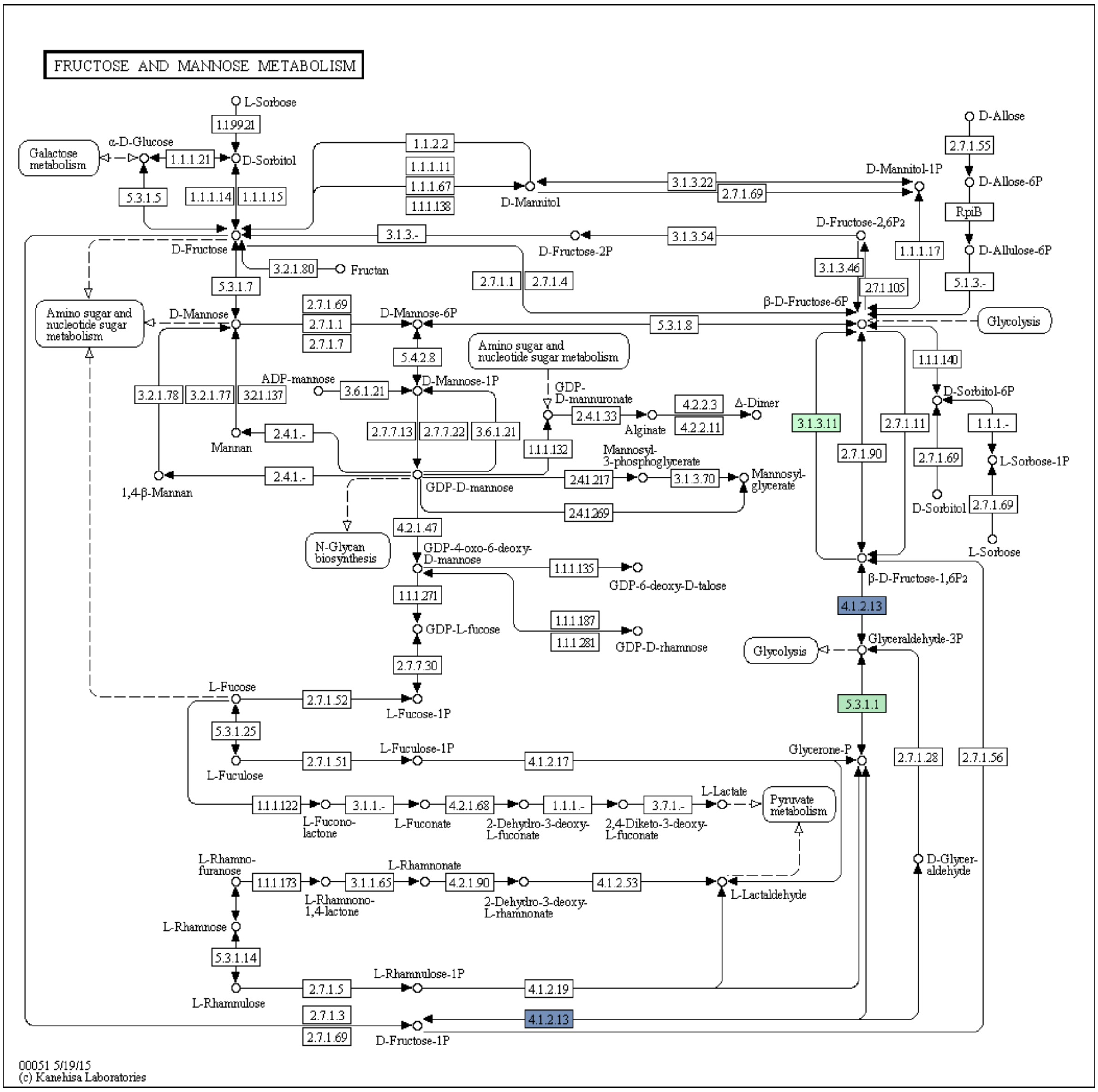

Thiamine metabolism: 3 proteins

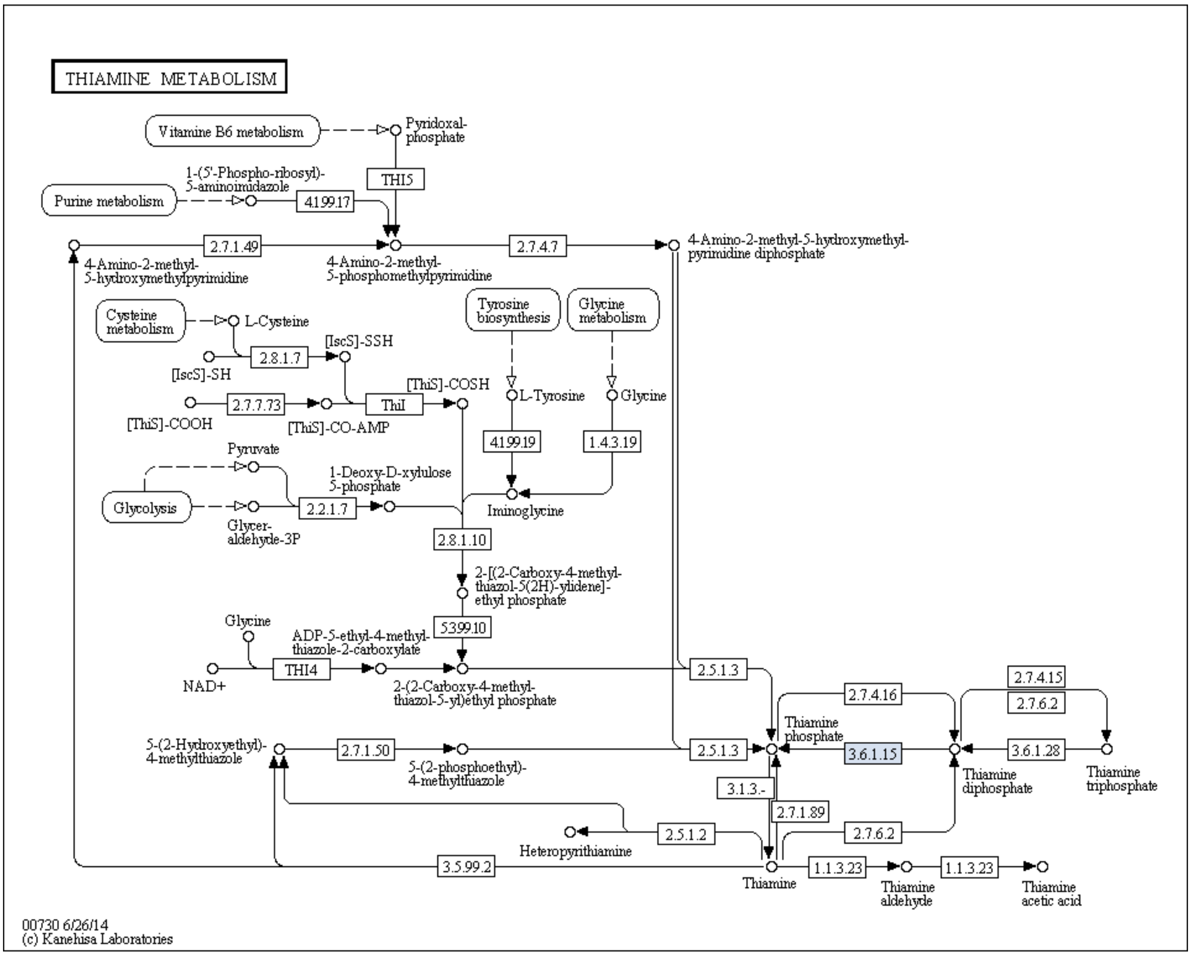

Phenylpropanoid biosynthesis: 3 proteins

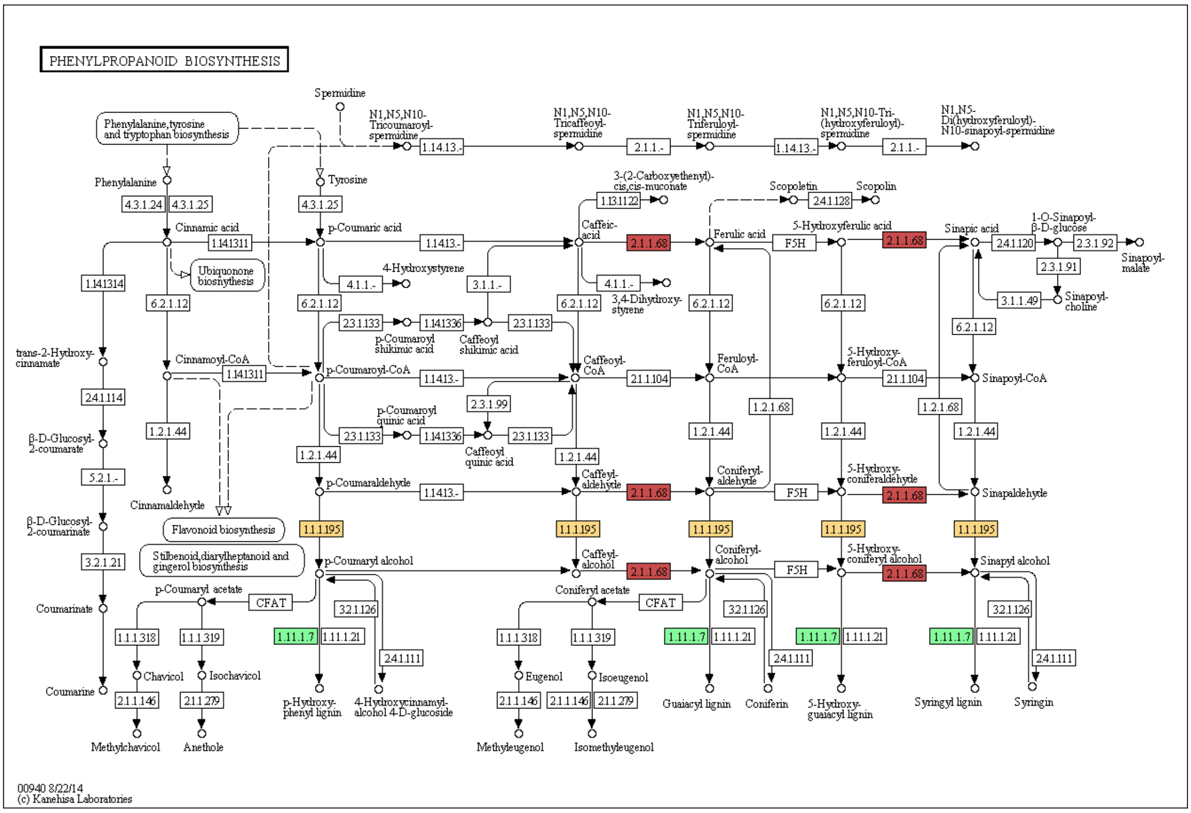

Phenylalanine metabolism: 2 proteins

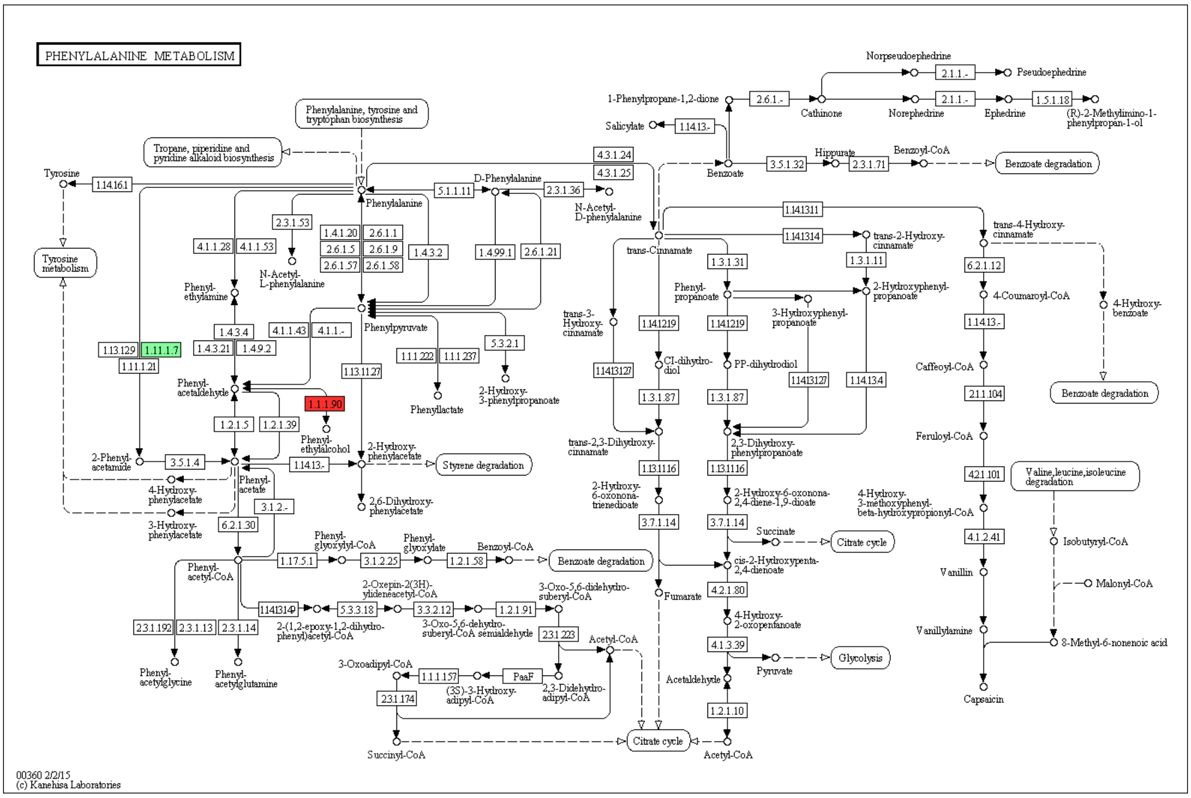

Starch and sucrose metabolism: 2 proteins

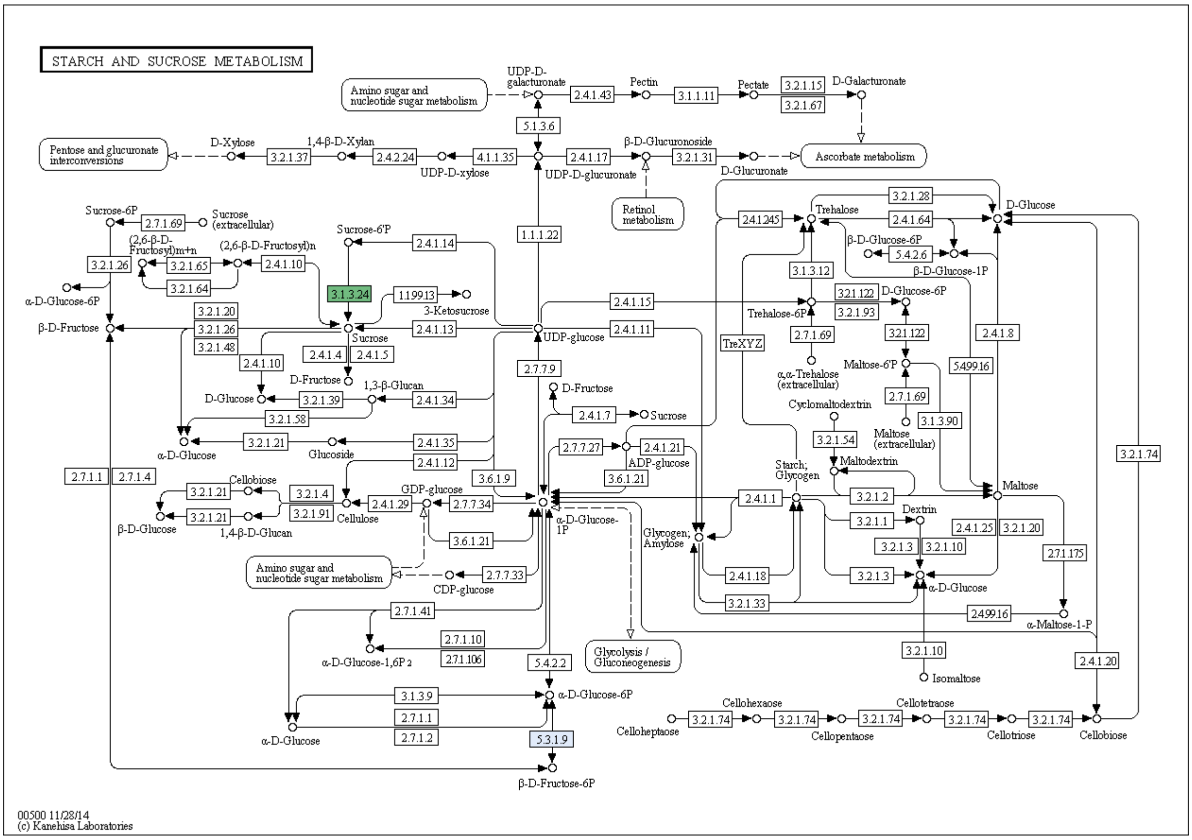

Methane metabolism: 2 proteins

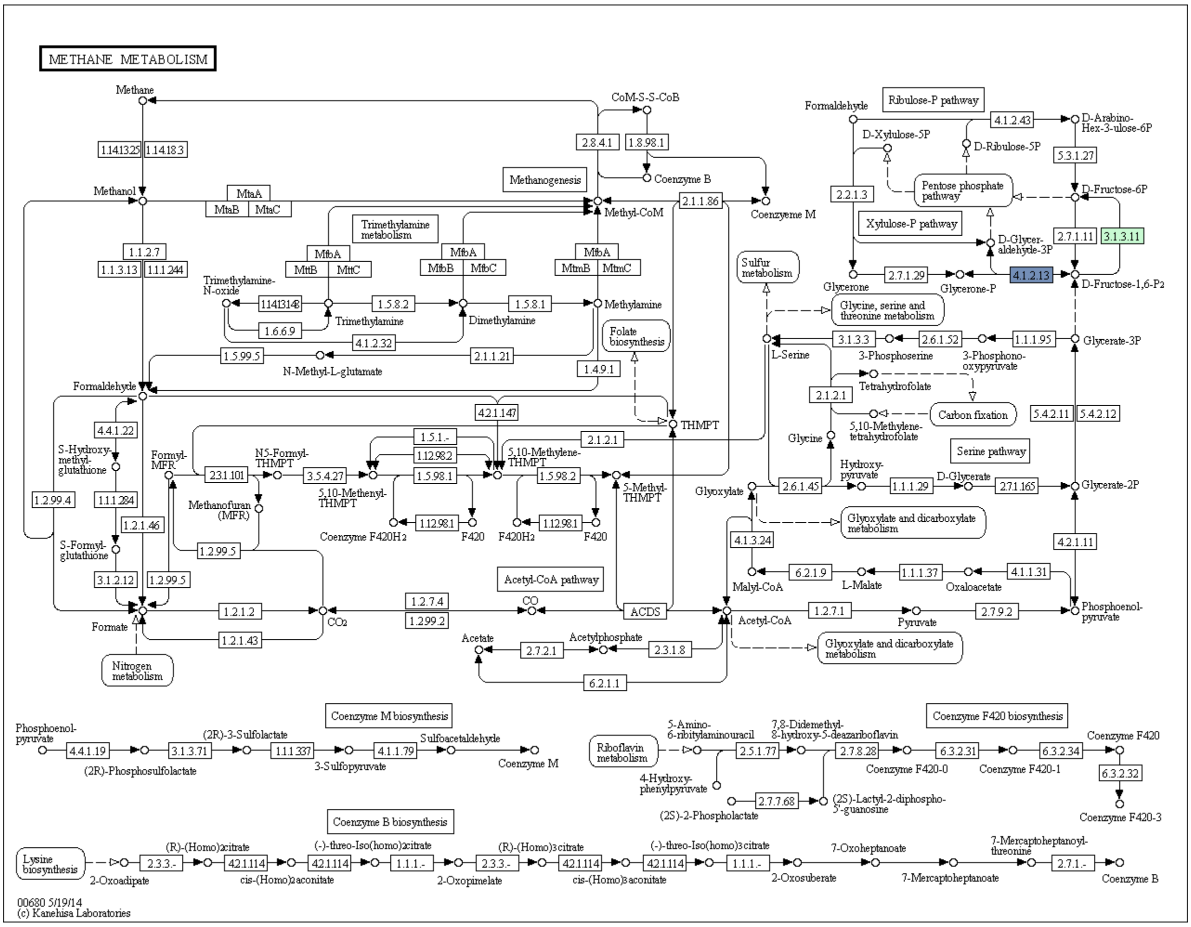

Supplementary Table S1. Classification of the 23 identified protein isoforms derived from 10 proteins.

Protein Accession[a]	Protein Description	Spot no[b]	p*I*[c]	*Mr*(Da)[d]
B6T2L2_MAIZE	Sedoheptulose-1,7-bisphosphatase	11	4.84	43753
		21	4.95	42674
		30	6.20	42563
C0PD30_MAIZE	Fructose-bisphosphate aldolase	52	4.53	38924
		22	5.48	38478
		25	5.62	38321
P00874 _MAIZE	Ribulose bisphosphate carboxylase large chain	14	6.93	53291
		43	6.11	55753
		46	6.95	53295
B6TEW2_MAIZE	Ferredoxin--NADP reductase, leaf isozyme	18	5.27	38437
		49	6.80	35351
A0A096SD19_MAIZE	Uncharacterized protein	3	4.95	83556
		32	4.99	83556
A0A059Q6M3_MAIZE	ATP synthase subunit alpha	26	5.35	59135
		6	5.90	59012
B4FRZ2_MAIZE	Pyridoxin biosynthesis protein ER1	54	6.59	37524
		7	6.60	37123
B6SRJ5_MAIZE	Bifunctional 3-phosphoadenosine 5-phosphosulfate synthetase 2	18	6.20	48531
		19	6.33	48658
Q6LBU9_MAIZE	Glyceraldehyde-3-phosphate dehydrogenase	33	6.75	41541
		41	6.88	41260
C0PDB0_MAIZE	Phosphoglycerate kinase	15	5.16	46149
		34	5.08	43237

a) Database accession numbers according to UNIProt.

b) Assigned spot number as indicated in Figure 2.

c, d) Experimental *p*I and mass (kDa) of identified proteins.

Supplementary Table S2. Primers used in qRT-PCR

Spot no	Primer pairs(5'--3')
1	1F: 5’-TAGGACCTGAACTGGCAACTACTCT-3’<br>1R: 5’-CTACCACCAACCCAATCAAACAT-3’
2	F: 5’- ACAGCCAGATTGTTCCAGCAT-3’<br>R: 5’- TGCCACAGCCTCAGTTCCTAT-3’
4	F: 5’- GCTGACCCCAGCAATATCCG-3’<br>R: 5’- TTCTTGGTTCGTCTGCCTAAAAT-3’
11	F: 5’- GCCCTCGCACGACCTACA-3’<br>R: 5’- AACATCTTCCCTTCCCCAATG-3’
12	F: 5’- GACGAGACGAAGCAGACCGA-3’<br>R: 5’- CCTTGATGTTTGGCAGGGTC-3’
13	F: 5’- AAGTTCGGCTCGTACTGGATTAA-3’<br>R: 5’- GAACCTTCACGATGACACGGA-3’
15	F: 5’- ACCAGAACATCACCGACGACA-3’<br>R: 5’- TTGCACCTGAATGCCAAGAA-3’
16	F: 5’- CATCGTCCGCACCTTATCCT-3’<br>R: 5’- CGTACTCATCGAGCACCACATT-3’
18	F: 5’- GGGGAGGTCGTCAAAGGAGT-3’<br>R: 5’- TGATTGTTGCGTTGGGGTCT-3’
19	F: 5’- CAGATCACGGGAAGAAGGTCC-3’<br>R: 5’- TCAGCTTGCAGGCTGTTGTAG-3’
27	F: 5’- CCCCGTTCCATTTCATTATCC-3’<br>R: 5’- TCGTGACATCGTCGTCTTTAGG-3’
36	F: 5’- GGACTTCGCCACCATTATCAAC-3’<br>R: 5’- GACCACATCACAATATCCCCAAC-3’
40	F: 5’- CCGATGAGACCCCTGAGTTG-3’<br>R: 5’- GGCTTGATGACATGCTCCTTC-3’
42	F: 5’- GCAGGGTCAGTTTTCTTGGG-3’<br>R: 5’- TGAGGAAAGAGTTCAGCGATACA-3’
44	F: 5’- ACGACGAGGTCATGCGCTAC-3’<br>R: 5’- TGGAGTCTCAAAGTTCTCAGGGTG-3’
52	F: 5’- ACACCTGCCTCAAGACCTGG-3’<br>R: 5’- TAATGGCTTCATGGGATGGG-3’
56	F: 5’- AATGCGAAGGATAACCCAAAGA-3’<br>R: 5’- AGTTCAAGCCCAGCAGGATG-3’
57	F: 5’- GCTGACCCCAGCAATATCCGTA-3’<br>R: 5’- TTCTTGGTTCGTCTGCCTAAAAT-3’
